# A novel lytic phage infecting MDR *Salmonella enterica* and its application as effective food biocontrol

**DOI:** 10.3389/fmicb.2024.1387830

**Published:** 2024-08-15

**Authors:** Anu Bala Jaglan, Ravikant Verma, Medhavi Vashisth, Nitin Virmani, B. C. Bera, R. K. Vaid, Taruna Anand

**Affiliations:** ^1^ICAR – National Research Centre on Equines, Hisar, India; ^2^Department of Zoology and Aquaculture, Chaudhary Charan Singh Haryana Agricultural University, Hisar, India

**Keywords:** *Salmonella enterica*, bacteriophage, *Caudoviricetes*, broad-host range, genome analysis, biofilm

## Abstract

*Salmonella enterica* is a foodborne pathogen associated with both typhoid and non-typhoid illness in humans and animals. This problem is further exacerbated by the emergence of antibiotic-resistant strains of *Salmonella enterica*. Therefore, to meet public health and safety, there is a need for an alternative strategy to tackle antibiotic-resistant bacteria. Bacteriophages or (bacterial viruses), due to their specificity, self-dosing, and antibiofilm activity, serve as a better approach to fighting against drug-resistant bacteria. In the current study, a broad-host range lytic phage phiSalP219 was isolated against multidrug-resistant *Salmonella enterica* serotypes Paratyphi from a pond water sample. *Salmonella* phage phiSalP219 was able to lyse 28/30 tested strains of *Salmonella enterica*. *Salmonella* phage phiSalP219 exhibits activity in acidic environments (pH3) and high temperatures (70°C). Electron microscopy and genome analysis revealed that phage phiSalP219 is a member of class *Caudoviricetes*. The genome of *Salmonella* phage phiSalP219 is 146Kb in size with 44.5% GC content. A total of 250 Coding Sequence (CDS) and 25 tRNAs were predicted in its genome. Predicted open reading frames (ORFs) were divided into five groups based on their annotation results: (1) nucleotide metabolism, (2) DNA replication and transcription, (3) structural proteins, (4) lysis protein, and (5) other proteins. The absence of lysogeny-related genes in their genome indicates that *Salmonella* phage phiSalP219 is lytic in nature. Phage phiSalP219 was also found to be microbiologically safe (due to the absence of toxin or virulence-related genes) in the control of *Salmonella enterica* serovar Typhimurium infections in the ready-to-eat meat and also able to eradicate biofilm formed by the same bacterium on the borosilicate glass surface.

## 1 Introduction

*Salmonella enterica* is a foodborne pathogen responsible for loss of appetite, poor growth, and diarrhea in poultry, enteric fever, and non-typhoidal gastrointestinal disorders in humans (CDC, 2019). *Salmonella enterica* serovar Typhimurium and Enteritidis are the leading agents of salmonellosis among the 2,600 serotypes of *Salmonella* that have been culpable for 25% of global diarrheal diseases. *Salmonella enterica* serovar Typhi and Paratyphi are associated with enteric fever related to travel, and they are responsible for nearly 220,000 deaths each year (Akhtar et al., 2015).

According to the latest report published by the European Food Safety Authority (EFSA), *Salmonella* spp. was the second most common cause of foodborne infections in humans (EFSA, 2019). Chicken is a widely consumed meat and has been found to be related to foodborne illness in humans (Desin et al., 2013). In addition, there have been 13,469 reported cases of meat-related foodborne outbreaks around the world from 1991 to 2021, causing 4,349 hospitalizations and 3,826 deaths primarily from *Salmonella* infections (Warmate and Onarinde, 2023). Overuse of antibiotics to control bacterial infections in different fields such as medicine, agriculture, and growth promotion in livestock has resulted in the emergence of multidrug-resistant bacteria. In recent years, the growing frequency of antibiotic-resistant *Salmonella* spp., especially fluoroquinolone resistance, has become a serious threat to public health as fluoroquinolones are a class of antibiotics most commonly used in treating *Salmonella* infections in both humans and animals due to their broad antibacterial activity (Pribul et al., 2016; Yu et al., 2021).

In addition, several studies have demonstrated the genetic relatedness between antibiotic-resistant bacteria isolated from farm animals and those found in humans. Noteworthy findings include the identification of the genetic relatedness of specific antibiotic-resistant genes. This molecular evidence substantiates the notion that antibiotic usage in animals contributes to the dissemination of resistance traits among human pathogens (Fasure et al., 2012; Sasaki et al., 2021). To address this problem, alternatives to antibiotics, such as bacteriophages, can become a promising solution.

Bacteriophages are the biological entities that were used in the pre-antibiotic era in the Western world, while in the Eastern world, mainly in Georgia, Poland, and the Soviet Union, they are continued in use along with antibiotics for the control of infectious diseases. In the past decade, phages have gained attention due to their successful use in the medical treatment of multidrug-resistant infections (Schooley et al., 2017; Patey et al., 2018). Lytic phages are generally recognized as safe (GRAS) for their use in the control of food pathogens such as *Listeria*, *Salmonella*, and *Escherichia coli*. However, temperate (lysogenic) phages were reported to be involved in the transfer of virulence and antibiotic-resistant genes in the bacterial population through horizontal gene transfer (Clokie et al., 2011; Touchon et al., 2016). Therefore, genome-wide characterizations of phages are very important before their application in therapy and biocontrol.

The main objective of this study was the isolation and characterization of *Salmonella* phage phiSalP219 to improve the microbiological safety of ready-to-eat meat products and as a biofilm eradication agent. For this, we isolated a lytic phage from a pond water sample, targeting non-typhoid and typhoid strains of *Salmonella enterica*. Through comprehensive whole-genome analysis, we determined the absence of virulence genes, antimicrobial resistance genes, and lysogeny-related genes within the phage genome. In addition, characteristics of *Salmonella* phage phiSalP219 such as stability at high temperatures and low pH, broad-host range, and antibiofilm activity make them a prime candidate for therapy and biocontrol of food pathogens.

## 2 Materials and methods

### 2.1 Bacterial strains and culture conditions

*Salmonella enterica* serovar Paratyphi MTCC735 was used to isolate the phage. Other bacterial strains used in this study to test the lytic spectrum and productivity of phage infection are listed in Table 1. All the isolates were propagated in nutrient broth (NB) at 37°C overnight with vigorous agitation at 180 rpm and preserved as glycerol stocks at −80°C. Antibiotic sensitivity test of all the bacterial isolates was conducted according to the Kirby-Bauer disk diffusion methods against 25 antibiotics [(Himedia, United States): ampicillin (AMP 10 μg), piperacillin (PI 100 μg), amoxicillin + clavulanate (AMC 20/10 μg), amikacin (AK 30 μg), gentamycin (GEN 10 μg), tetracycline (TE 30 μg), chloramphenicol (C 30 μg), aztreonam (AT 30 μg), cefoxitin (CX 30 μg), ceftazidime (CAZ 30 μg), cefepime (CPM 30 μg), cefpodoxime (CPD 10 μg), imipenem (IPM 10 μg), meropenem (MRP 10 μg), kanamycin (K 30 μg), tobramycin (TOB 10 μg), azithromycin (AZM 30 μg), ofloxacin (OF 5 μg), nalidixic acid (NA 30 μg), norfloxacin (NX 10 μg), ciprofloxacin (CIP 5 μg), fosfomycin (FO 50 μg), sulfafurazole (SF 300 μg), cotrimoxazole (COT 25 μg), and colistin (CL 10 μg)] as per Clinical and Laboratory Standards Institute (CLSI) guidelines (CLSI, 2016). Based on their susceptibilities against tested antibiotics, bacteria were categorized as multidrug-resistant (MDR) when they showed non-susceptibility to at least one agent in three or more antimicrobial classes (Magiorakos et al., 2012).

**Table 1:** Antibiotic-resistant profile of the *Salmonella* strains used for host range analysis and EOP studies.

Sr. No.	*Salmonella* strains	Species		Antibiotic-resistant profile	Lytic activity of phiSalP219	EOP	
						EOP value	Productivity
1	Fo55a	*Salmonella* spp.		CX, NA	+++	0.75	High
2	Fo55b	*Salmonella* spp.		NA	++	0.008	Inefficient
3	Fo55c	*Salmonella* spp.		NA	+++	0.8	High
4	Fo55d	*Salmonella* spp.		CX, NA	−	−	−
5	Fo55e	*Salmonella* spp.	MDR	CAZ, AT, MRP, K, AZM, TE, NA, CIP, COT, CL,	+	0.0005	Inefficient
6	VTCCBAA501	*S. typhimurium*	MDR	PI, AMC, CPM, CX, AK, CIP, CL	++	0. 25	Medium
7	VTCCBAA508	*S. typhimurium*		PI, CAZ	+	0.00006	Inefficient
8	VTCCBAA576	*S. enterica* Gallinarum	MDR	CAZ, CPM, AK, NA, CL	+	0.05	Low
9	VTCCBAA579	*S. enteritidis*	MDR	NA, CIP, CL	+	0.1	Medium
10	VTCCBAA582	*S. gallinarum*		OF, NA	++	0.06	Low
11	VTCCBAA583	*S. gallinarum*	MDR	CPD, MRP, NA	+++	0.22	Medium
12	VTCCBAA584	*S. gallinarum*		NA, NX	+++	0.24	Medium
13	VTCCBAA585	*S. gallinarum*		OF, CL	+++	0.3	Medium
14	VTCCBAA586	*S. gallinarum*	MDR	CPD, CL, NA	++	0.1	Medium
15	VTCCBAA587	*S. gallinarum*		NA, CIP	+++	0.22	Medium
16	VTCCBAA588	*S. gallinarum*		NA, NX, CIP	++	0.000003	Inefficient
17	VTCCBAA589	*S. gallinarum*	MDR	GEN, K, NA, NX, CIP, SF, COT	++	0.13	Medium
18	VTCCBAA590	*S. gallinarum*	MDR	CPD, GEN, K, NA, NX, CIP, SF, CL	+++	0.3	Medium
19	VTCCBAA591	*S. gallinarum*	MDR	CPD, GEN, NA, NX, CIP, COT, CL	+++	0.24	Medium
20	VTCCBAA592	*S. gallinarum*	MDR	CPD, NA, CIP, CL	++	0.1	Medium
21	VTCCBAA593	*S. gallinarum*		NA, CL	+++	0.3	Medium
22	VTCCBAA594	*S. gallinarum*		NA, CIP	+++	0.6	High
23	VTCCBAA595	*S. gallinarum*		NA	+++	1	High
24	VTCCBAA596	*S. enterica* Gallinarum		NA, CIP	+++	1	High
25	VTCCBAA702	*S. enteritidis*		−	+++	0.7	High
26	VTCCBAA714	*S. enteritidis*		IMP, MRP	++	0.000006	Inefficient
27	VTCCBAA722	*S. enteritidis*		−	++	0.09	Low
28	MTCC735	*S. paratyphi*	MDR	CL, AMC, FO	+++	1	High
29	MTCC3232	*S. typhimurium*	MDR	PI, MRP, CL	+	0	Inefficient
30	MTCC3223	*S. typhimurium*	MDR	CL, PI, CAZ, CPM, CX, IPM, MRP	−	−	−

### 2.2 Bacteriophage isolation and preservation

Pond water samples collected from Hisar City, India (N 29° 7′, 36.264″, E 75° 47′, 20.364″), were used for the isolation of bacteriophage. Sample enrichment, phage isolation, and purification were carried out by following the methods described by Anand et al. (2018) using the *Salmonella enterica* serovar Paratyphi MTCC735 as a host. In brief, 40 mL of sample water was mixed with log-phase bacterial culture (5 mL), 5X NB (5 mL), and 5 mM of CaCl_2_ (Calcium chloride) and incubated overnight at 37°C with shaking at 220 rpm. The phage-enriched lysate was centrifuged, and the supernatant was passed through a 0.22-μm pore-size polyvinylidene difluoride (PVDF) syringe filter (Millipore). Afterward, a total of 10 μL of the filtrate was spotted on the bacterial lawn prepared by pouring 3 mL of molten agar containing 300 μL of log-phase bacterial culture using the double-agar overlay (DLA) method, and plates were incubated at 37°C for 3–4 h under static conditions. The qualifying filtrate was used to purify phages by picking and resuspending a single, well-separated plaque. A single plaque was lifted from the agar plate and resuspended in 100 μL of saline magnesium (SM) buffer [NaCl: 5.8 g/L; MgSO4: 2.0 g/L; 1 M Tris (pH 7.5): 50 mL/L; and gelatin (2%): 5 mL/L], and four rounds of purifications were performed to obtain the purified and homogenous plaques. A large volume (50 mL) of log-phase host culture infected with purified phage was incubated overnight at 37°C with shaking at 220 rpm. Lysate obtained after centrifugation was precipitated with NaCl and polyethylene glycol (PEG) to obtain a high-titer phage suspension (Sambrook and Russell, 2006). The bacteriophage suspension was used for titer estimation and preserved as glycerol stocks at −80°C, followed by accession in the Bacteriophage Repository at the National Centre for Veterinary Type Culture, ICAR-NRCE, Hisar, Haryana.

### 2.3 Phage characterization

#### 2.3.1 Transmission electron microscopy (TEM) of the bacteriophage

A total of 10 μL of high-titer-purified phage suspension (1 × 10^11^ PFU/mL) was loaded onto carbon-coated nickel grids for 5 min to allow binding of bacteriophages for TEM analysis. The excess suspension was wiped out with Whatman filter paper, and the grids were washed with sterile distilled water. The dried grids were then negatively stained with 2% uranyl acetate. Phage was observed using a transmission electron microscope (JEOL JEM-1011, Jeol, United States) operating at 80 kV.

#### 2.3.2 Temperature and pH sensitivity

The stability of phage phiSalP219 at different temperatures was evaluated by incubating 100 μL of phage suspension (2×10^11^ PFU/mL) at 25, 37, 45, 55, 60, 65, 70, or 80°C for 60 min. For the pH stability test, phage suspension was mixed with pH buffers ranging from 2 to 10 in a 1:10 v/v ratio to a final titer of 0.5×10^11^ PFU/mL, and 100 uL of this mixture was incubated at room temperature for 60 min.

After treatment, the viability of phage in each treatment lot was tested by determining the number of infectious phage particles present per ml of treated suspension using a PFU assay. All these experiments were conducted in triplicate, and the results were recorded as the means of three replicates.

#### 2.3.3 Host range determination

*Salmonella* phage phiSalP219 was tested for its lytic activity on different *Salmonella* strains as indicated in Table 1. Host range was determined by spotting 10 μL of the concentrated phage suspension (0.5×10^11^ PFU/mL) on a lawn of log-phase culture of various strains using double-layer agar. The spotted area was observed for the zone of cell lysis, and the lytic activity of bacteriophage was assessed with a scaling system (Kutter, 2009; Fong et al., 2017), where (−) indicates a zone with complete turbidity (no infection), (+) indicates mild lysis, (++) indicates moderate lysis, and (+++) indicates strong lysis.

#### 2.3.4 Efficiency of plating

The efficiency of plating (EOP) was performed to measure the productivity of phage infection on various bacterial strains as described previously by Viazis et al. (2011). Only those bacterial strains that were found to be sensitive to phage phiSalP219 in spot assay were used to conduct the EOP test. For this, 100 μL of diluted (10^6^ to 10^9^ fold) phage lysate was plated with 300 μL of selected bacterial strains using double-layer agar assays and incubated overnight at 37°C. After incubation, the titer of phage on different strains was calculated, and EOP was subsequently determined using the formula: EOP = Phage titer on test bacteria/Phage titer on propagating host bacteria. Based on their EOP values, the productivity of phage infection on various strains was classified as follows: high (EOP 0.5 to 1.0), medium (EOP 0.1 to <0.5), low (EOP 0.001 to <0.1), and inefficient (EOP < 0.001) (Khan Mirzaei et al., 2015).

#### 2.3.5 One-step growth curve experiment

The growth curve of phage phiSalP219 was determined as explained earlier by Zhao et al. (2019), with some modifications. *Salmonella enterica* serovar Paratyphi MTCC 735 was challenged with phage phiSalP219 at an MOI of 0.01. For this, host bacteria were grown at 37°C to achieve the mid-log phase to achieve a CFU count of 10^7^ CFU/mL, and phage was taken at a concentration of 10^5^ PFU/mL. Next, 1 mL suspension of both bacteria and phage was incubated for 5 min to allow the phages to adsorb; then, it was centrifuged at 8,000×*g* for 5 min to remove unabsorbed phage particles in the suspension. The pellet was then washed twice, finally resuspended in 50 mL of sterile NB, and incubated at 37°C while shaking at 200 rpm. From this suspension, aliquots of 100 μL were collected every 5 min for 70 min after infection and were plated using the double-layer agar (DLA) method. Phage titer was calculated by counting the number of plaques from each plate. Burst size was calculated as the ratio of the final titer of liberated phage particles at the plateau to the initial number of phage particles during the latent period (Czajkowski et al., 2014).

### 2.4 Time-kill curve assay

The bactericidal activity of phage phiSalP219 against planktonic cells of *S. enterica* serovar Paratyphi MTCC 735 and *S. enterica* serovar Typhimurium VTCCBAA501 was assessed as described previously (Esmael et al., 2021) with some modifications. In brief, in a 96-well microtiter plate, phage phiSalP219 was mixed with MTCC 735 and VTCCBAA501 at different MOIs of 0.0001, 0.001, 0.01, 0.1, 1, 10, or 100, respectively. Positive control wells contained MTCC 735 and VTCCBAA501, while negative control wells contained nutrient broth only. The microtiter plate was incubated at 37°C in a microplate reader with mild agitation, and bacterial growth was monitored at an interval of 30 min for 6 h by measuring optical densities at 600 nm using a Multiskan GO Microplate Spectrophotometer, using SkanIt™ Software (Thermo Scientific, Waltham, MA, United States, ver. 1.01.12x).

### 2.5 Phage DNA extraction, library preparation, and whole-genome sequencing

Bacteriophage DNA was extracted using the protocol described previously by Anand et al. (2018), and the concentration was determined using a Qubit 4 Fluorometer and NanoDrop spectrophotometer. Whole-genome sequencing of bacteriophage was carried out using Illumina NovaSeq 6,000 platform (Illumina, San Diego, CA, USA) and Nanopore GridION X5 (Oxford Nanopore Technologies, Oxford, United Kingdom) technology. Sequencing libraries for Illumina were prepared according to the QIASeq FX DNA library preparation protocol using the QIASeq FX DNA kit. For nanopore sequencing library preparation, genomic DNA was end-primed by using the NEBnext ultra II end repair kit and cleaned up with AMPure beads. Native barcoding of end-primed samples was carried out with EXP-NBD104(ONT). Finally, the equimolar concentration of the barcode sample was ligated with a sequencing adaptor, purified with AMPure beads, and eluted in 15 μL of elution buffer. Raw reads obtained from Illumina and nanopore sequencing were de-multiplexed using Bcl2Fastq software v2.20^1^ and guppy-v2.3.4 (Wick et al., 2019), respectively. Trimgalore-v0.4.0^2^ tool and Commander v2.0.20^3^ were used in Illumina sequencing, and Porechop-v0.2.3^4^ and Nanofilt-v2.8.0^5^ were used in Nanopore sequencing for trimming the raw reads and to assess their quality. The long read obtained from Nanopore sequencing and the short read obtained from Illumina were assembled using Unicycler v0.4.8. A hybrid sequencing approach was carried out for *de novo* assembly. Detailed information on the raw reads statistics is given in Supplementary Table S1.

### 2.6 Genome annotation

The assembled genome of bacteriophage was analyzed by RASTtk (Brettin et al., 2015) pipeline for predicting and annotating open reading frames (ORFs). Functional prediction and validation of the ORFs were performed using BLASTp on the NCBI non-redundant protein (nr) sequences database. Predicted protein sequences were analyzed against InterProScan (Paysan-Lafosse et al., 2023) and CDD (Lu et al., 2020) for conservative domain identification. Putative tRNAs were identified using tRNAscan-SE by using bacterial sequence source and default parameter (Lowe and Chan, 2016). ARG genes were screened by using CARD (Alcock et al., 2020), and CG View software was used for genomic map visualization (Grant et al., 2023). The complete genome of *Salmonella* phage phiSalP219 was submitted to the GenBank database, and its accession number is PP595732.

### 2.7 Phylogenetic analysis

A phylogenetic analysis was conducted to investigate the relatedness of phage phiSalP219 with the known phages of the subfamily *Vequintavirinae*. Genome-BLAST Distance Phylogeny method in the Virus Classification and Tree Building Online Resource (VICTOR) was used for the whole-genome phylogenetic analysis and tree building (Meier-Kolthoff and Göker, 2017). The resulting intergenomic distances (including 100 pseudo-bootstrap replicates each) were used to infer a balanced minimum evolution tree with branch support via FASTME, including subtree pruning and regrafting postprocessing for the formula D0 (Farris, 1972). The tree was visualized using ggTree (Yu, 2020). For determining the phylogenetic positions and DNA packaging strategies, phage-conserved gene sequences (major capsid protein and terminase large subunits) were used. The phylogenetic tree of their amino acid sequence was constructed in MEGA X 10.2.6 software (Kumar et al., 2018) using the Clustal W alignment algorithm by neighbor joining method with 1,000 bootstrap replications (Thompson et al., 1994). Comparative genome alignment with four reference phages (*Salmonella* phages PVP-SE1, SSE-121, and NINP13076 and *Escherichia* phage 4MG) was performed using Mauve 2.4.0 (Darling et al., 2004).

### 2.8 Phage stability in food samples

To check the stability of *Salmonella* phage phiSalP219 in foods, phage stability experiments were conducted according to a previously described method (Guenther et al., 2012). In brief, packed chicken ham and salami were obtained from the local market, sliced into 1 g pieces using a surgical blade, and sterilized using UV radiation for 30 min. In total, 50 μL volume of phage suspension was added to reach a final titer of 1×10^9^ PFU/mL. Inoculated samples were incubated for 0, 6, 12, 24, and 48 h separately at 28°C. After each time interval, the respective sample was transferred into 5 mL of PBS and homogenized. Then, the phage titer was measured using the double-layer agar method and calculated as PFU/mL.

### 2.9 Biological control of *S. typhimurium* in food samples

Packaged/processed chicken ham and salami were obtained from a local supermarket and then sliced aseptically in the laboratory. The food samples were cut into pieces (1 g) using a sterile scalpel blade and then sterilized by exposing them to 30 min of UV light. After UV exposure, we checked the presence of bacterial contamination in the food sample, before starting the experiment and found that UV exposure sterilized the food sample. Then, the food sections were placed in the center of sterile Petri dishes and inoculated with *S. typhimurium* VTCCBAA501 (As *S. typhimurium* is the leading agent of foodborne infection, therefore to find out the biocontrol capability to phage phiSalP219 in the control of *S. typhimurium* strain in the food sample, this strain of *S. typhimurium* was used) to a final viable count of 4.3 log10 CFU/g and was allowed to adsorb for 15–20 min. Phage suspension was then applied to the sample surface with an MOI of 10^4^. These samples were incubated at 4°C or 28°C for 48 h. During incubation, the samples were taken at 0, 6, 12, 24, and 48 h from each group and transferred to 5 mL of PBS buffer. They were homogenized with a sterile handheld homogenizer and vortexed. The proportions of recoverable bacteria among the control group and the experimental group were determined using the direct spread plate method by plating 100 μL of successive serial dilutions of the homogenized tissue and calculating the CFU/mL by counting the number of colonies.

### 2.10 Antibiofilm activity of bacteriophage on borosilicate glass surface

For assessing the biofilm eradication capability of phage phiSalP219, all the bacterial strains as shown in Table 1 were tested for their biofilm-forming ability; out of these strains, VTCCBAA501 was chosen based on its good biofilm formation capacity. Overnight grown culture of VTCCBAA501 in Tryptic Soy Broth (TSB) was re-inoculated in a 12-well culture plate containing 2X TSB broth with 1% glucose. Borosilicate glass coverslips of size 18–22 mm were immersed in the wells containing inoculated bacterial culture and placed at 37°C in a static incubator for 72 h. After incubation, the media in the wells were removed, and the wells were washed twice with sterile PBS. Bacteriophage suspension (10^11^ PFU/mL) at a volume to submerge the coverslips completely was added in the wells and placed at 37°C for 24 h under static conditions. Following the treatment, the coverslips were removed and washed with PBS twice. The control group was treated with SM buffer without any phage in it. Then, the coverslips were washed with PBS and placed in a 2.5% glutaraldehyde solution for 1 h at 4°C to fix the biofilm. Thereafter, biofilm was dehydrated by placing coverslips in the ethanol gradient [25, 50, 75, 90, and 100% (2X)] (Nikara et al., 2020). Dehydrated coverslips were affixed to a copper stub, sputtered with gold for 1 min, and then viewed at an accelerating voltage of 5–10 kV in a JSM-7610F Plus Scanning Electron Microscope, Jeol, Japan.

### 2.11 Statistical analysis

The data analyses of all the experiments were performed using GraphPad Prism 8.0.2 (GraphPad Software, La Jolla, CA, United States). All experiments were conducted in triplicate. The two-way ANOVA with Bonferroni’s multiple comparisons was used to determine the significance among groups at a significance level of *p* < 0.05.

## 3 Results

### 3.1 Antibiotic sensitivity test

The outcomes of the antibiotic sensitivity test revealed that 13 *Salmonella* isolates belonging to the serovar Typhimurium, Enteritidis, Paratyphi, and Gallinarum were MDR (Refer to Table 1), while 73.33% of the *Salmonella* isolates displayed resistance to either one or two antibiotics falling within the fluoroquinolone class of antimicrobial agents.

### 3.2 Bacteriophage isolation and purification

**Figure 1:**
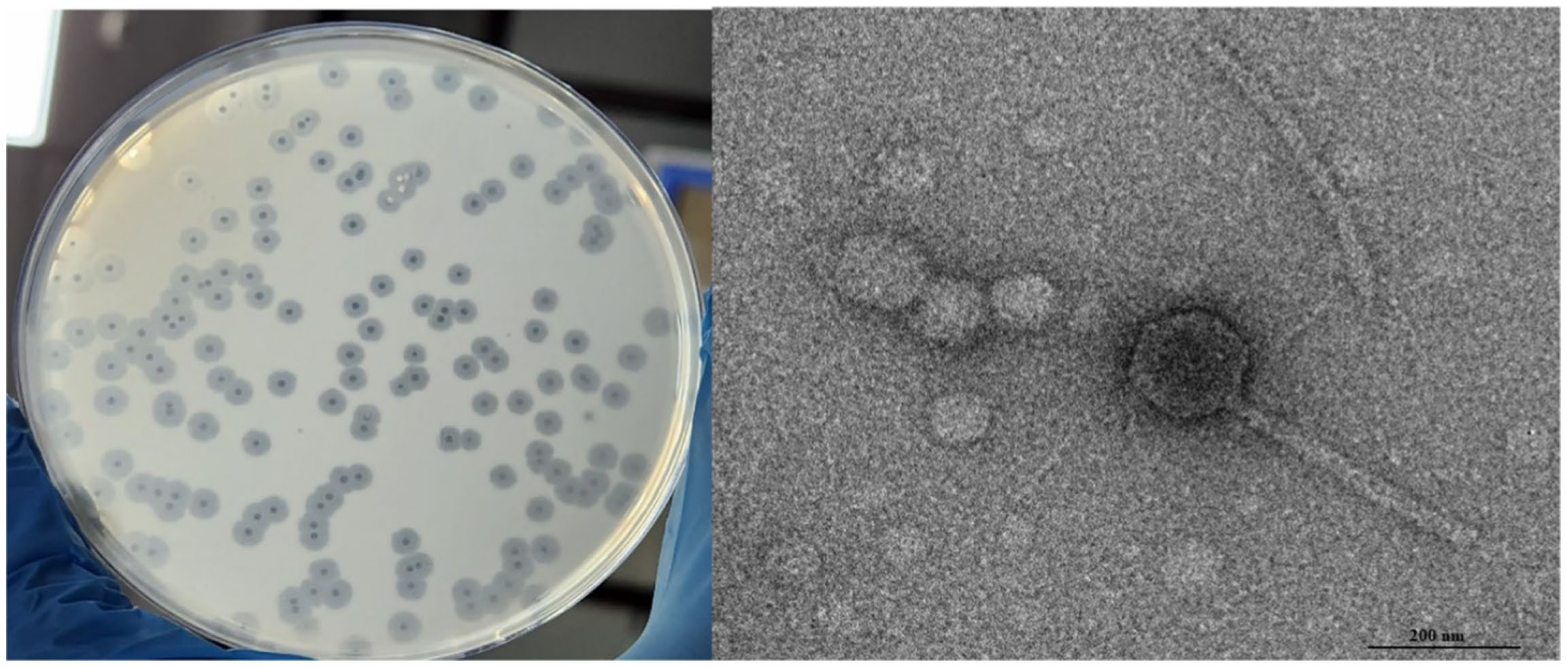
Plaque morphology and transmission electron micrograph of *Salmonella* phage phiSalP219.

*Salmonella* phage phiSalP219 was isolated from the pond water sample using *Salmonella enterica* serovar Paratyphi MTCC735 as a host and was named *Salmonella* phage phiSalP219 according to Adriaenssens and Brister (2017). Phage phiSalP219 was preserved as glycerol stocks in the Bacteriophage Repository of the National Centre on Veterinary Type Culture–National Research Centre on Equine NCVTC-NRCE, Hisar (see Figure 1).

### 3.3 Characterization of phage

**Figure 2:**
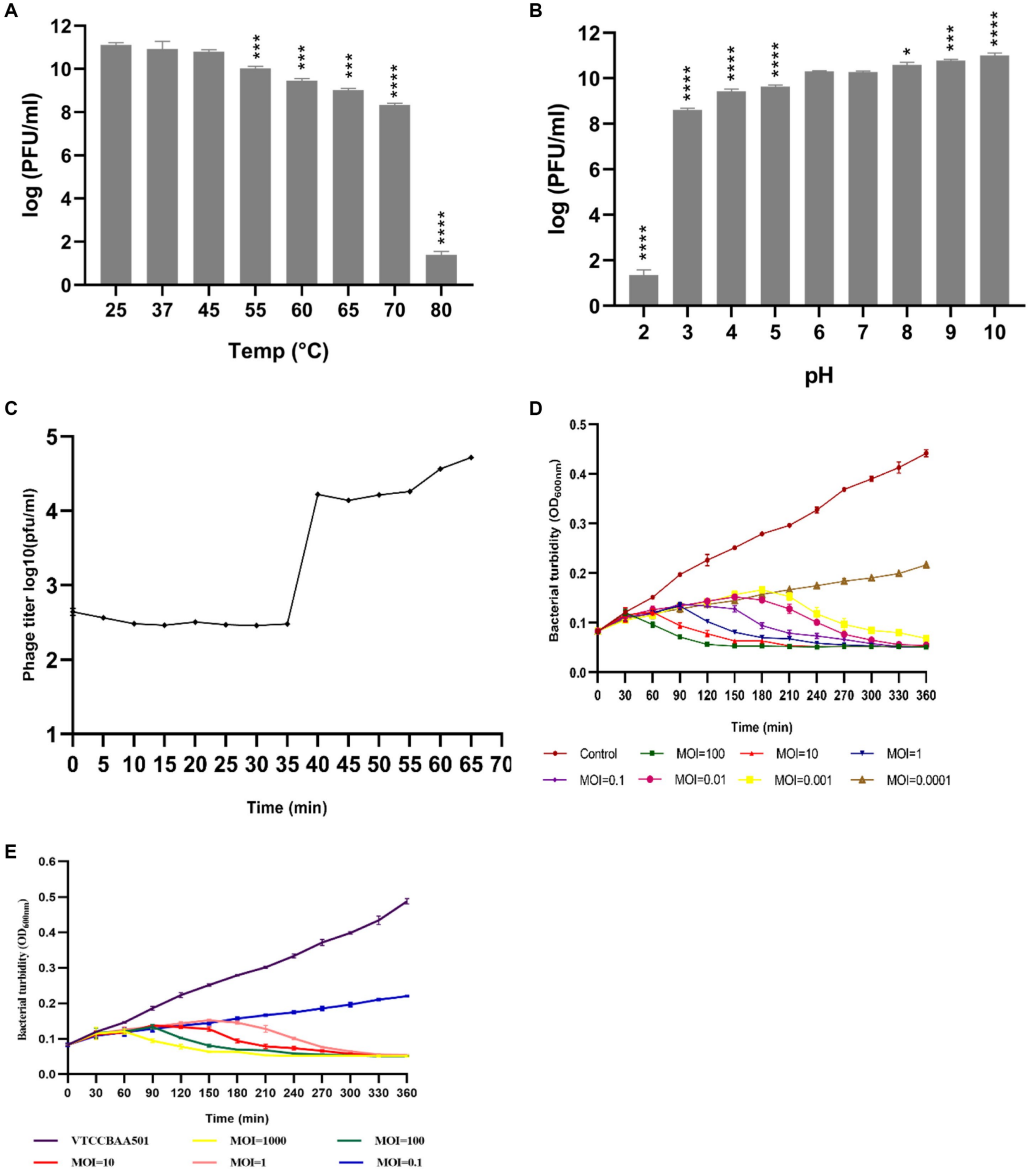
Sections **(A,B)** depict the temperature and pH stability of phage phiSalP219, and significance levels are depicted as ****p < 0.0001, ***p < 0.001, and *p < 0.01 calculated by ANOVA with Dunnett’s test. Section **(C)** shows the one-step growth curve, and Sections **(D,E)** show the time-kill curve of phage phiSalP219 with *Salmonella Paratyphi* and *Salmonella Typhimurium*, respectively. Error bars depict the standard deviation from the mean.

*Salmonella* phage phiSalP219 produced clear plaques with a surrounding halo. Phage head and diameter were measured by the software used for capturing TEM images only using the inbuilt scale. Phage dimensions are shown as the average of measurements taken from five phage particles of the same phage. TEM divulged that phage phiSalP219 belongs to the class *Caudoviricetes*, with an icosahedral head of 155.9 ± 7.2 diameter and a long tail of 365.6 ± 5.0.5 diameter (Figure 1). pH sensitivity analysis showed that phage phiSalP219 was stable without any significant change in their viable titer at pH values ranging from 6 to 10, while reductions in phage titer were detected at pH 2, pH 3, and pH 4 (Figure 2B). Similarly, the results obtained from the temperature stability assay demonstrated that phage remained stable at temperatures ranging from 25 to 45°C. Beyond 45°C up to 70°C, a gradual significant reduction in phage stability in terms of phage titer was observed (as shown in Figure 2A), while treatment at 80°C led to huge stability loss in phage titer (9.74 log reduction).

Host range evaluation by spot assay revealed that phage phiSalP219 was able to lyse 28/30 tested strains of *Salmonella* with varied strength of lysis (Table 1). Hence, it was characterized as a broad-host range phage, which was further supported by the results from the EOP study. In the EOP study, we found that phage phiSalP219 showed high productivity against 7/28, medium productivity against 12/28, low productivity against 3/28, and inefficient productivity against 6/28 tested strains of the *Salmonella enterica*.

The results from the one-step growth curve indicate that phage phiSalP219 required 65 min to complete its infection cycle with a latent period of 35 min, followed by a short rise period in the next 5 min, and the burst size was calculated to be 68 phage particles per infected bacterial cell (Figure 2C). To find out the best lytic efficiency of *Salmonella* phage phiSalP219 for carrying out the biocontrol of *S. typhimurium* (VTCCBAA501) in ready-to-use food samples and in the eradication of biofilm formed by *S. typhimurium* (VTCCBAA501), *Salmonella* phage phiSalP219 was tested against MTCC 735 and VTCCBAA501 at different Multiplicity of infection (MOIs), that is, 1,000, 100, 10, 1, 0.1, 0.01, 0.001, and 0.0001, and respective time-kill curves were obtained. In the case of MTCC 735, phage phiSalP219 inhibited bacterial growth up to MOI of 0.001, while in the case of *S. typhimurium* VTCCBAA501, phage phiSalP219 inhibited bacterial growth up to MOI of 1 (Figures 2D,E).

### 3.4 Bioinformatic analysis

The detailed genomic information of *Salmonella* phage phiSalP219 is given in Supplementary Tables S2, S3. The genome size of the phage phiSalP219 was 146,994 bp, and the overall G + C content was 44.5%. Most CDS of phage phiSalP219 (218/250) began with ATG, followed by GTG (17/250) and TTG (15/250). Among termination codons, TAA (144/250) was the most common, followed by TAG (84/250) and TGA (22/250).

**Figure 3:**
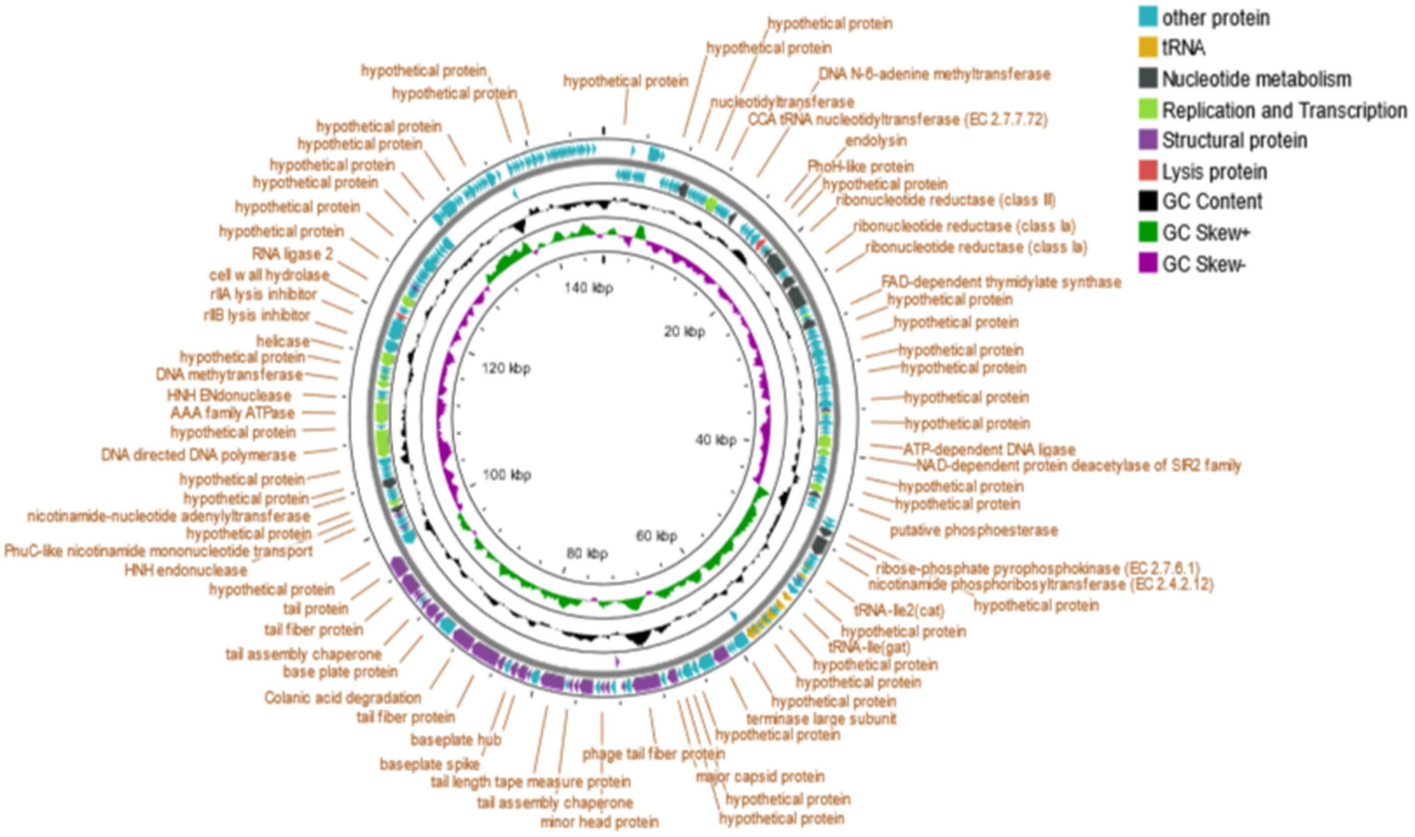
Genome map of *Salmonella* phage phiSalP219.

Bioinformatic analysis revealed that the genomes of phage phiSalP219 contained ~250 putative ORFs (including ~100 functional, ~ 19 uncharacterized, and ~ 131 hypothetical proteins) and 25 tRNAs. The predicted CDS were divided into five groups according to their functions: nucleotide metabolism, DNA replication and transcription, structural proteins, lysis proteins, and other proteins (Figure 3).

#### 3.4.1 Nucleotide metabolism

A total of 11 CDS were predicted to be related to the nucleotide metabolism, including nucleotidyltransferase (CDS21), ribonucleotide reductase (CDS45, 46, 49, 50), FAD-dependent thymidylate synthase (CDS55), putative phosphoesterase (CDS 86), ribose-phosphate pyrophosphokinase (CDS92), nicotinamide phosphoribosyltransferase (CDS93), PnuC-like nicotinamide mononucleotide transporter (ORF161), and nicotinamide-nucleotide adenylyltransferase (CDS167).

#### 3.4.2 Replication and transcription

Twelve CDS were predicted to be involved in DNA replication and transcription, including CCA tRNA nucleotidyltransferase (CDS29), DNA N-6-adenine methyltransferase (CDS36), HNH homing endonuclease (CDS53, 162, 177), NAD-dependent protein deacetylase of the SIR2 family (CDS79), DNA cytosine methyltransferase (CDS85), transcriptional regulator (CDS73), DNA ligase (CDS78, 180), RNA ligase 2 (CDS192), and helicase (CDS185).

#### 3.4.3 Structural proteins

Twenty-eight CDS were predicted to belong to be involved in packaging and morphogenesis, including major capsid protein (CDS122), minor head protein (CDS130), head fiber protein (CDS126), terminase large subunit (CDS117), tail fiber protein (CDS124, 142, 143, 147, 152, 153), tail completion or Neck1 protein (CDS129), tail sheath (CDS132), virion structural protein (CDS133, 138), tail assembly chaperone (CDS134, 148), tail length tape measure protein (CDS136), hypothetical membrane protein (CDS150, 156, 160), baseplate hub (CDS139), baseplate spike (CDS140), base plate protein (CDS146), and membrane protein (CDS72, 102, 127, 151, 197).

#### 3.4.4 Lysis protein

Two CDS were predicted to belong to this cassette, including endolysin (CDS42) and cell wall hydrolase (CDS189).

#### 3.4.5 Other proteins

The remaining CDS which were predicted to be hypothetical and other proteins are put together in this category.

### 3.5 Phylogenetic analysis

**Figure 4:**
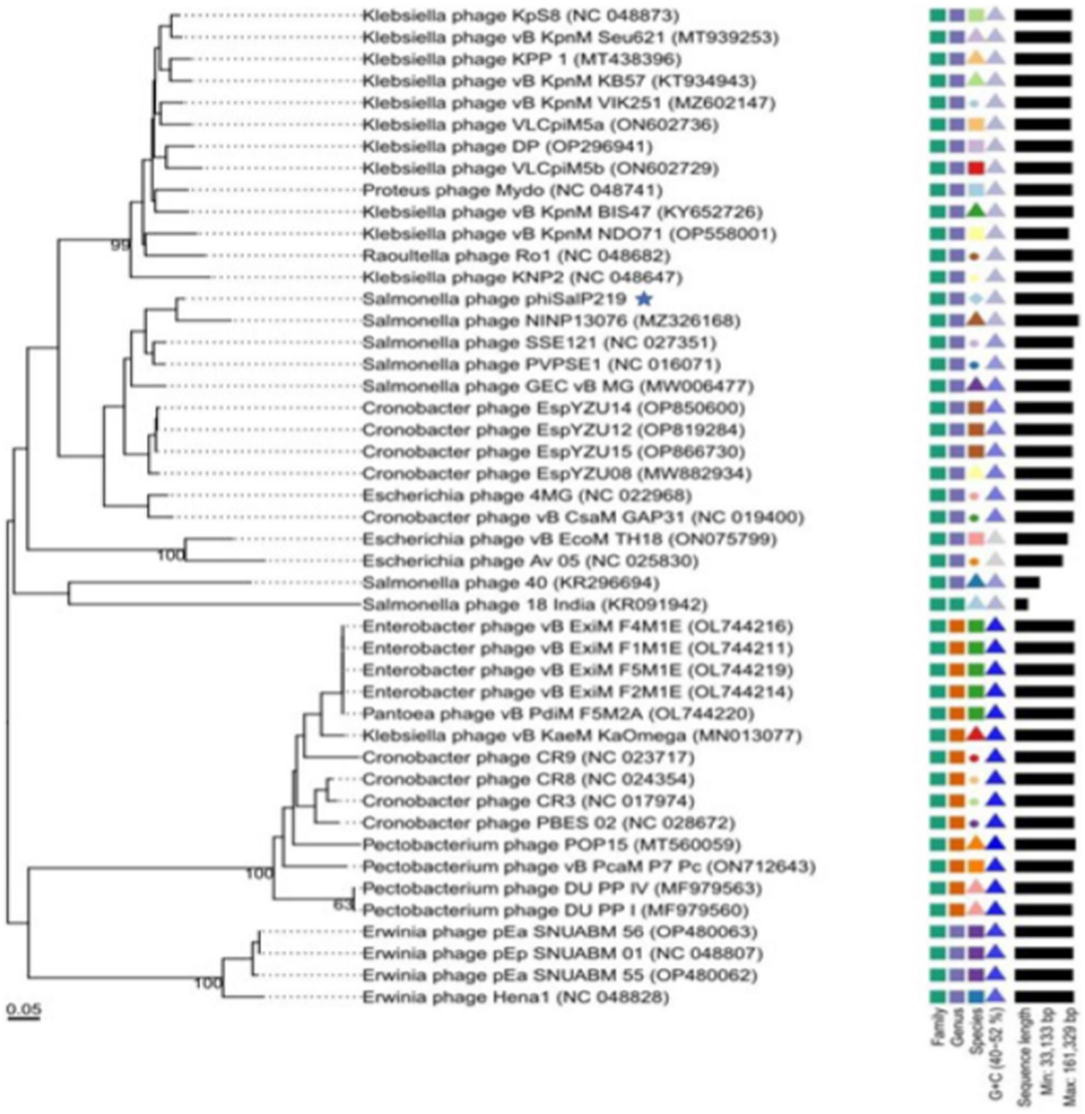
Comparative genomic analysis of *Salmonella* phage phiSalP219 using VICTOR.

**Figure 5:**
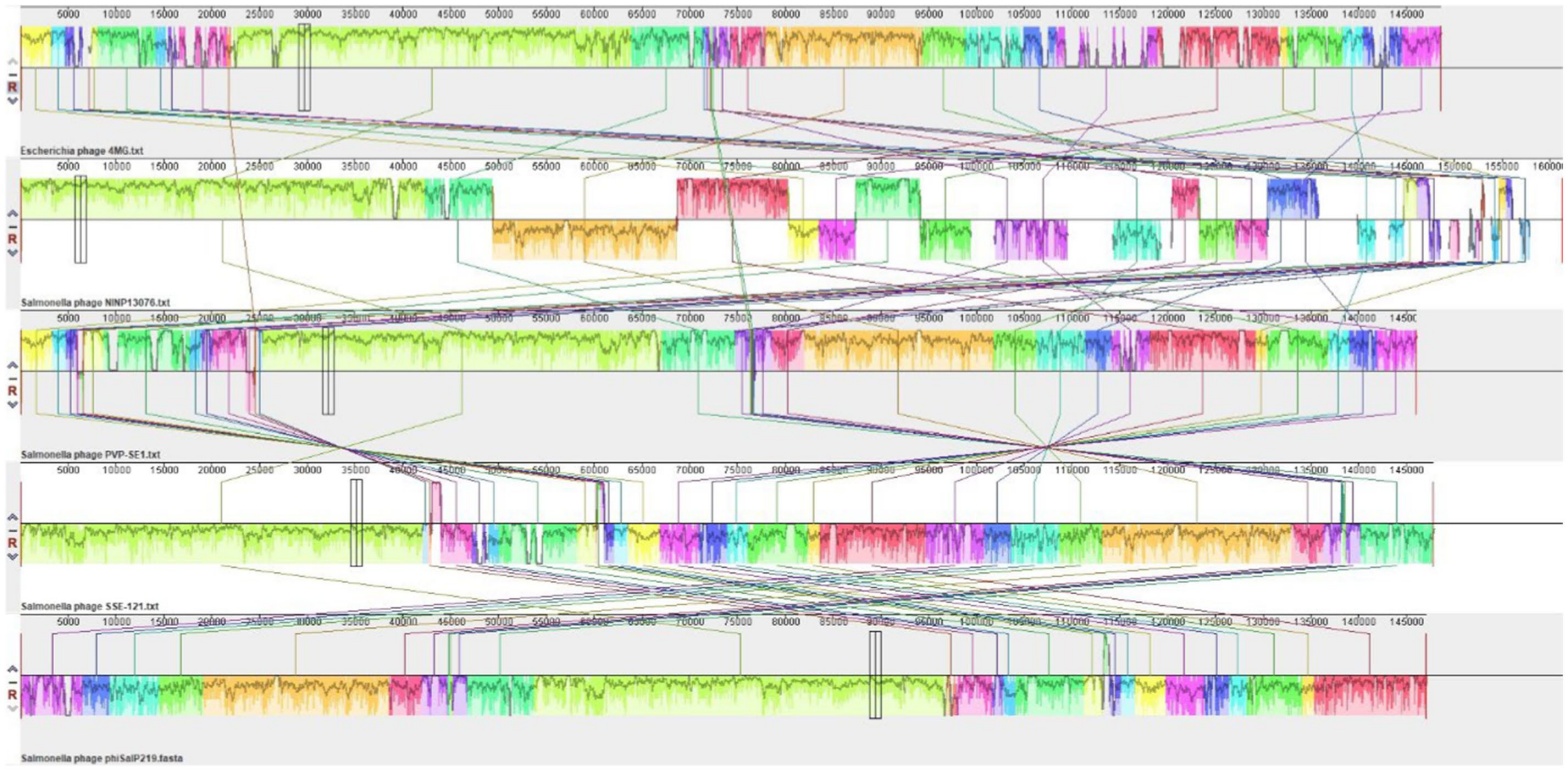
DNA level alignment of the genome of *Salmonella* phage phiSalP219 with the genomes of *Salmonella* phages PVP-SE1, SSE-121, NINP13076, and *Escherichia* phage 4MG.

**Figure 6:**
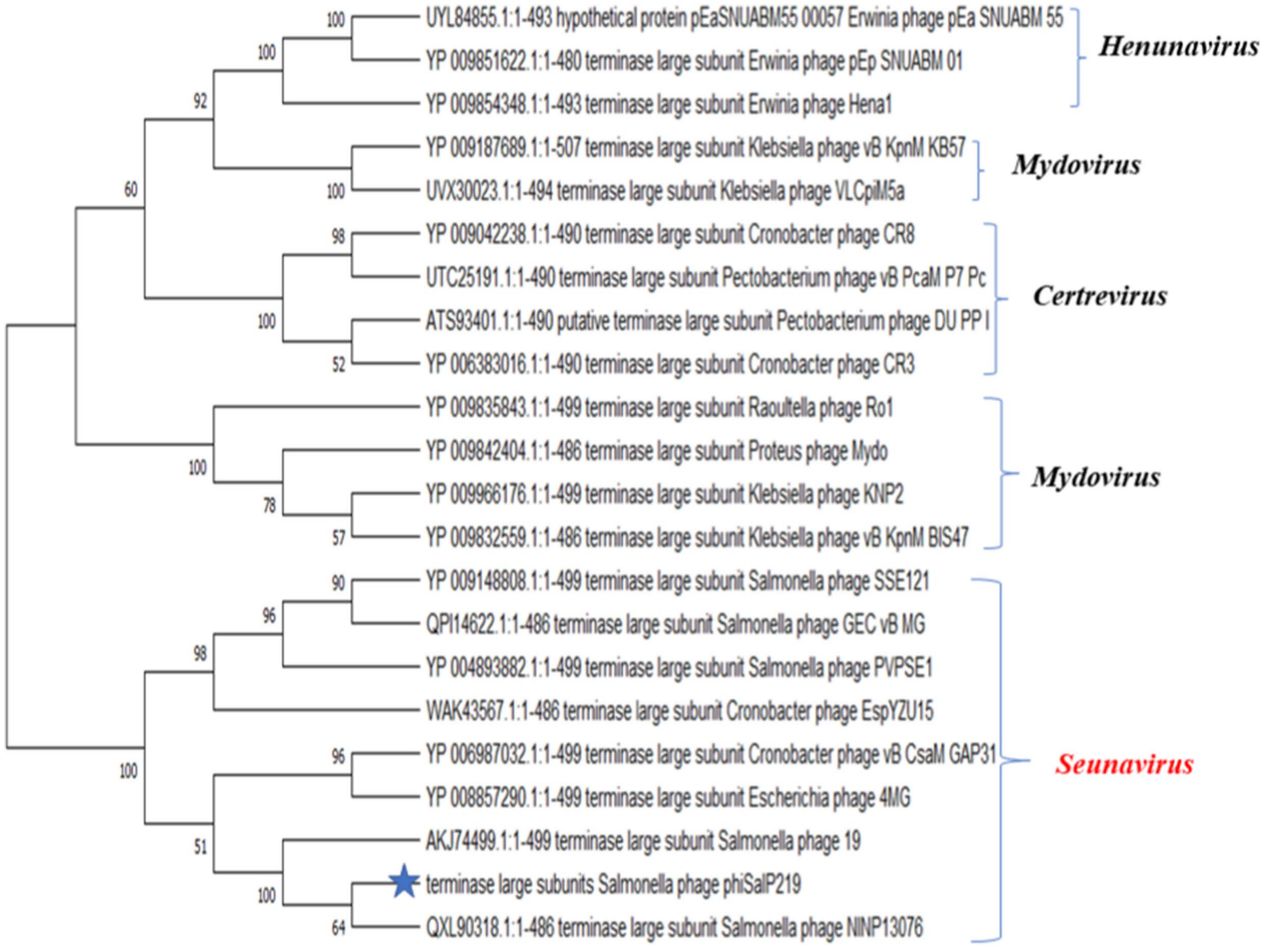
Phylogenetic relationship of *Salmonella* phage phiSalP219 to other phages based on terminase large subunit protein sequences.

**Figure 7:**
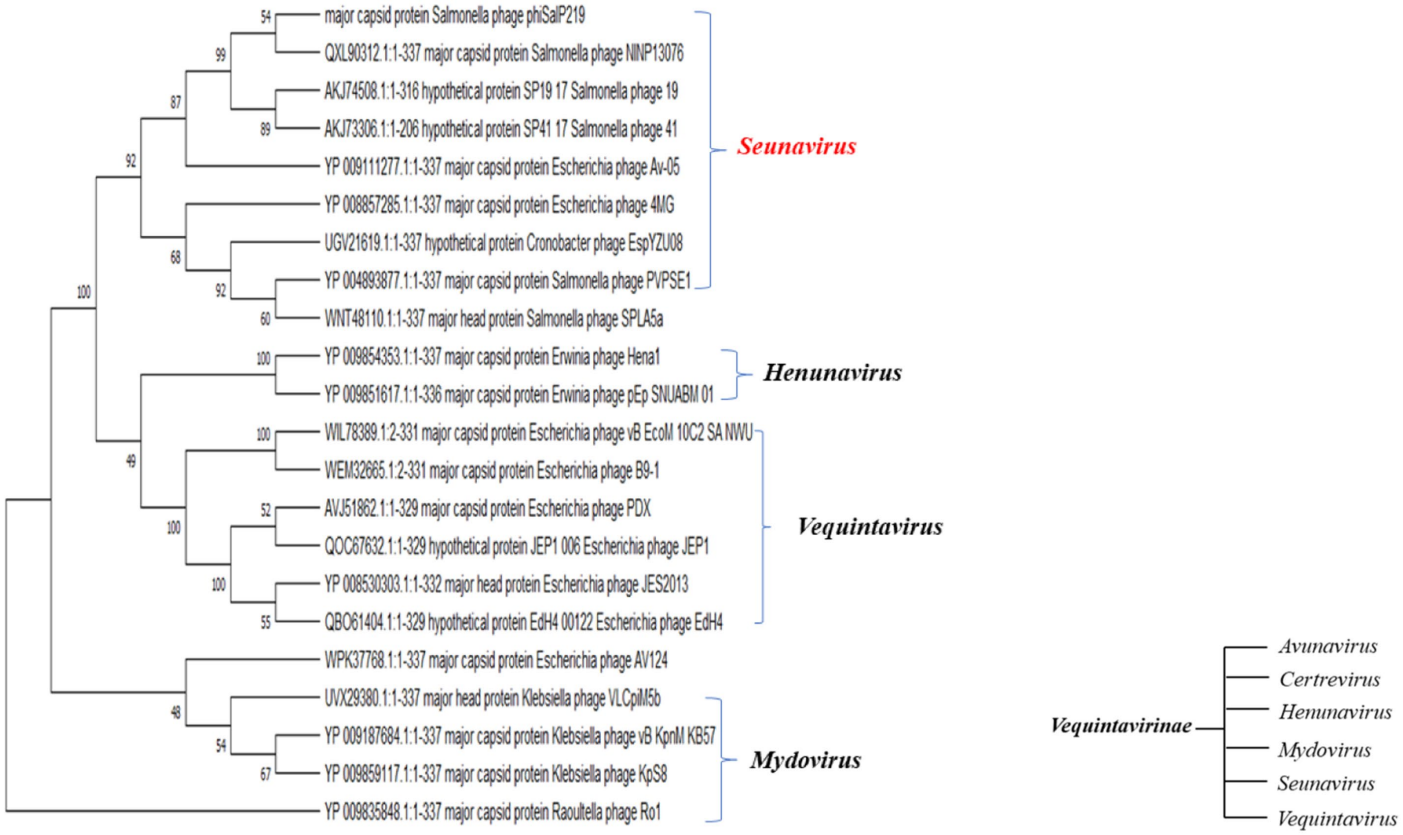
Phylogenetic relationship of *Salmonella* phage phiSalP219 to other phages based on major capsid protein sequences (in the side of this phylogenetic tree, we show the lower taxon of subfamily *Vequintavirinae*).

A BLAST search revealed that phage phiSalP219 showed >97% identity with *Salmonella* phages belonging to the subfamily *Vequintavirinae* (NCBI: txid1914851; *Vequintavirinae*, *Caudoviricetes*). To further investigate and visualize the genomic distance between *Salmonella* phage phiSalP219 and other *Vequintavirinae* phages, phylogeny was performed using whole-genome and phage-conserved gene sequences (major capsid protein and terminase large subunit). The whole-genome phylogeny constructed with the VICTOR server showed that *Salmonella* phage phiSalP219 was the new member of the *Vequintavirinae* subfamily, which was further supported by the results of the phylogenetic analysis of the major capsid protein and large terminase subunits (see Figures 4–7).

### 3.6 Phage stability and biological control of *Salmonella* in food samples

**Figure 8:**
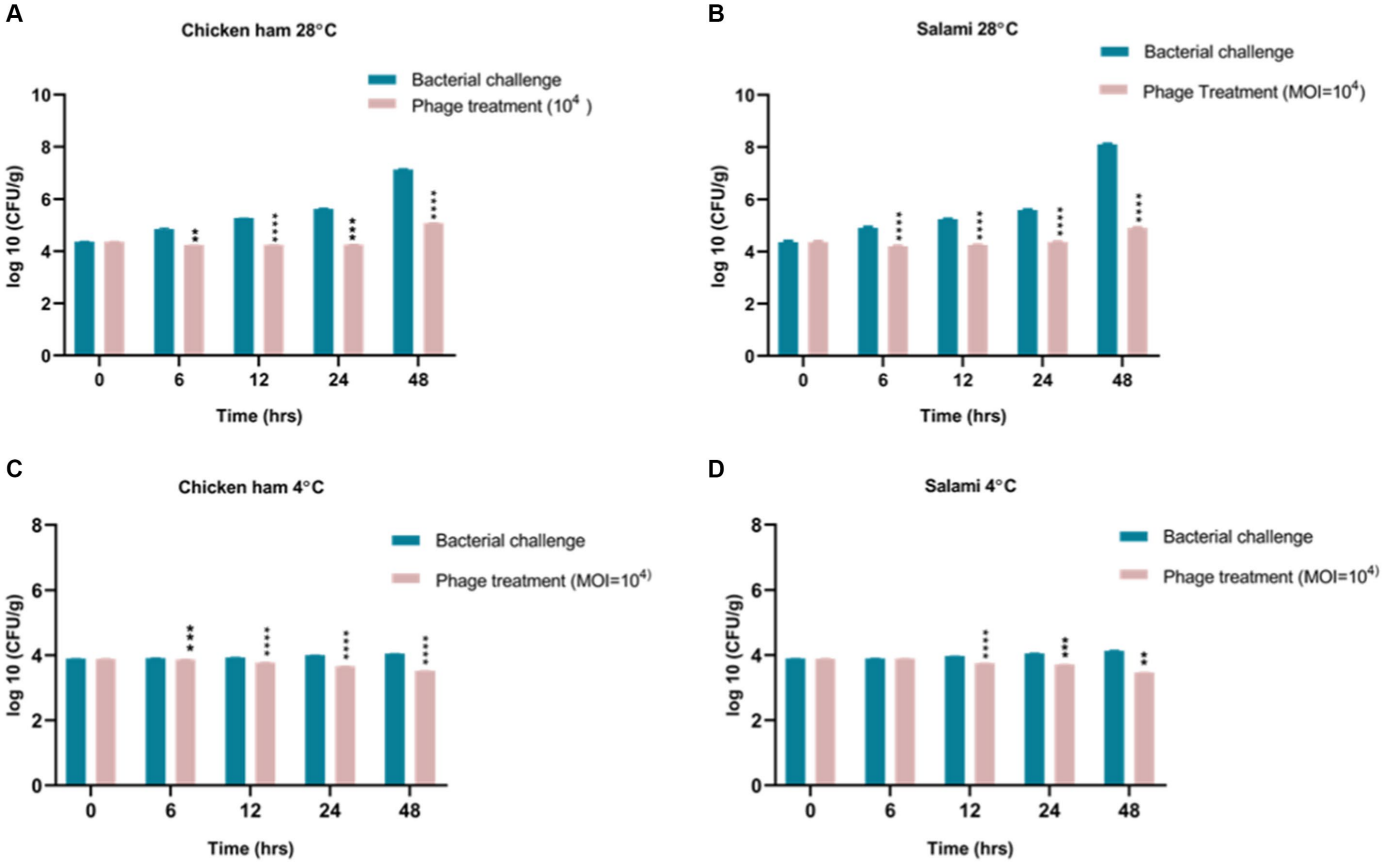
Biocontrol of *Salmonella* infection in ready-to-eat food. Panels **(A,C)** represent control of *Salmonella* infection in chicken ham with phage treatment at 4°C and 28°C. Panels **(B,D)** represent control of *Salmonella* infection in salami with phage treatment at 4°C and 28°C.

We observed that phage remained stable on the food matrices, and no significant change in phage concentrations was observed in the salami and chicken ham. Both the samples were inoculated with phage at 1×10^9^ PFU/mL final concentration, and after 48 h, we found 0.8×10^8^ PFU/mL concentration of phage in salami and 0.15×10^8^ PFU/mL of phage in chicken ham. We also found that *Salmonella* phage phiSalP219 had a significant antibacterial effect on *S. enterica* serovar Typhimurium VTCCBAA501 in different food matrices under different temperature conditions when applied at MOI = 10,000 (*p* < 0.05). For *Salmonella*-contaminated salami samples administered with phage phiSalP219 at an MOI of 10,000, the viable bacterial count was reduced by 0.661 log10 CFU/mL at 4°C and 3.191 log10 CFU/mL at 28°C compared to the control group after 48 h of incubation (Figures 8A,C), while for *Salmonella*-spiked chicken ham samples, phage phiSalP219 applied at an MOI of 10,000 reduced the viable *Salmonella* count by 0.529 log10 CFU/mL at 4°C and 2.046 log10 CFU/mL at 28°C relative to the control group after 48 h of incubation (Figures 8B,D). Hence, it is observed that the antibacterial effect of phage phiSalP219 was higher at 28°C than at 4°C.

### 3.7 Antibiofilm activity of *Salmonella* phage phiSalP219 on borosilicate glass surface

**Figure 9:**
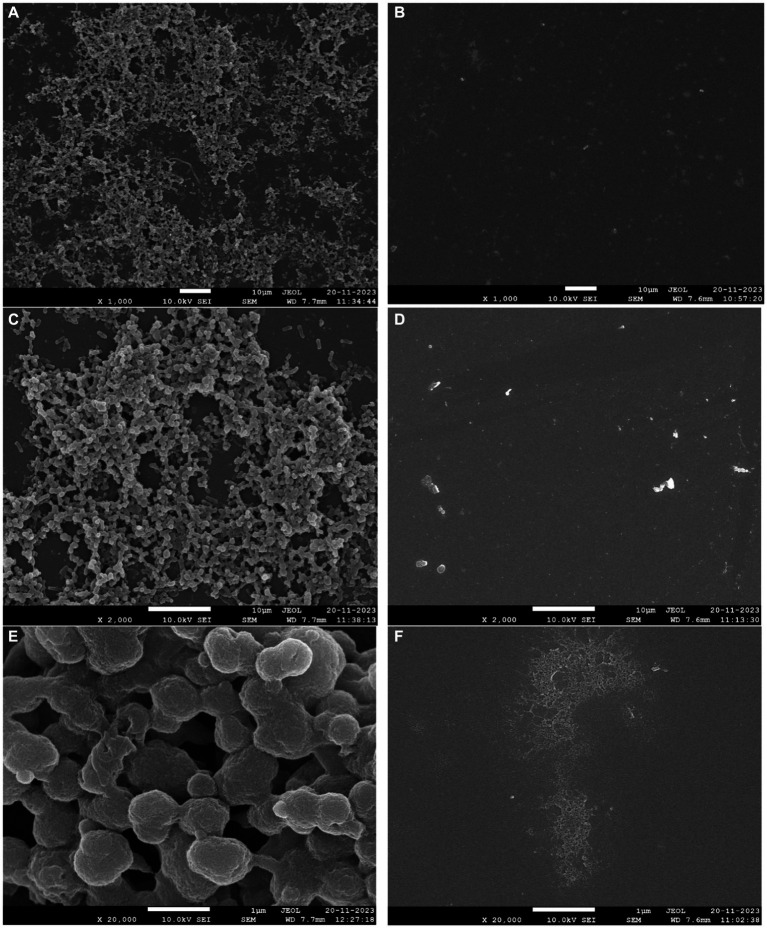
Biofilm eradication ability of phage phiSalP219. Panels **(A–C)** represent 3-day-old biofilm developed on the borosilicate glass coverslips. Multi-layer indicated by an arrow **(A,B)** and extra polysaccharides **(C)** observed in the matured biofilm of *S. typhimurium*. Panels **(D–F)** represent eradicated biofilm and cell debris indicated by an arrow after treatment with phage for 24 h.

The 72-h-old biofilms developed on the glass surface were used to demonstrate the process of phage-mediated eradication. The biofilms that formed on the coverslips have projections with many layers of different densities and heights. These come from the cell layers below (Figures 9A,B). Within the gelatinous extracellular polymeric substance (EPS) matrix, cells exhibited adhesion to both each other and the surfaces of the coverslip (Figures 9A–C). After the application of bacteriophage, the biofilm was clearly eliminated (as shown in Figures 9D–F), resulting in the presence of only cellular debris and the absence of any intact cellular structure.

## 4 Discussion

The application of bacteriophages, or phages, in the poultry and meat industry as a means to treat and control bacterial infections represents a viable solution to counteract the menace posed by emerging antibiotic-resistant bacteria. However, the presence of virulence, resistance, or lysogenic genes in the phage genomes reduces their therapeutic value. Therefore, along with their structural and physiological characterization, it becomes necessary to understand their genomic features before applying them as a therapeutic or biocontrol agent. In this study, we isolated the *Salmonella* phage phiSalP219 against the *Salmonella enterica* serovar Paratyphi, which significantly lysed multidrug-resistant (MDR) strains of *Salmonella enterica* and demonstrated a broad spectrum of lysis. It was able to lyse 28 out of 30 *Salmonella* strains tested, and most of the tested strains belonged to the serovar Typhimurium, Enteritidis, Paratyphi, and Gallinarum. Hence, it is a polyvalent lytic phage that lyses the important serovar of *Salmonella* as compared to other *Salmonella* phages such as *Salmonella* phage PVP-SE1 and *Salmonella* phage NINP13076 (Santos et al., 2010; Panpatil et al., 2022).

Their stability assay showed that phage could survive in an acidic environment having a pH of 3 and temperatures up to 70°C. Several studies demonstrate that most of the lytic phages were stable at pH ranges of 5–9 (Jung et al., 2017; Mondal et al., 2022). Hence, phage phiSalP219 can serve as a good candidate for biocontrol in varied conditions in the food processing industry. The one-step growth curve of phage phiSalP219 showed a latent (35 min) and rise period (5 min) and a larger burst size of 68 phage particles per infected bacterial cell than the reference phage PVP-SE1. Therefore, these parameters, such as the large burst size and short latent period, interpret the lytic capacity of phage (Santos et al., 2014).

However, before applying it to food production, we need to understand its genetic behavior; therefore, we analyzed its genome. The average GC content of the phage is 44.5%, which is lower than their host GC content (52%). The high genome sequence similarity to other *Salmonella* phages established via BLASTn and VICTOR analysis revealed that the *Salmonella* phage phiSalP219 could be considered to belong to the existing genus *Seunavirus* of the subfamily *Vequintavirinae*. The prevailing standard for delineating species boundaries among viruses is currently based on a threshold of 95% genome sequence similarity (Adriaenssens and Brister, 2017). This criterion stipulates that viruses belonging to the same species exhibit a nucleotide-level divergence of less than 5% (Adriaenssens and Brister, 2017). *Salmonella* phage phiSalP219 shows 98% genome sequence similarity with 94% query cover to *Salmonella* phage NINP13076, 97% identity with 85% query cover to *Salmonella* phage SSE-121, and 94% identity with 84% query cover to *Salmonella* phage PVP-SE1. Therefore, phage phiSalP219 belonged to the genus *Seunavirus*. For species classification in prokaryotic genomes, average nucleotide identity (ANI) is used as a golden parameter (Konstantinidis and Tiedje, 2005; Goris et al., 2007; Jain et al., 2018). Organisms showing more than 95% ANI are considered to belong to the same species. We used the tool: https://www.ezbiocloud.net/tools/ani (Yoon et al., 2017) to calculate ANI between phiSalP219 and similar genomes, and it was revealed that phiSalP219 has the highest ANI value of 98.83% with *Salmonella* phage NINP13076; based on their high ANI value, it seems that phage phiSalP219 belongs to the unclassified species same as that of NINP13076. After reporting a large gene rearrangement on their genome comparison and analysis of phage DNA and protein sequence using EMBOSS Stretcher,^6^ we concluded that phage phiSalP219 belongs to the different species (Adriaenssens and Brister, 2017). It has been further analyzed by VIRDIC (Supplementary Figure S1 and Supplementary Table S4) and found that phage phiSalP219 was classified as a new species different from that of other 21 phages.^7^ However, the genomic organization of lytic *Salmonella* phage NINP13076 (Kumar et al., 2022) and phage phiSalP219 differs in terms of the distribution of ORFs between the direct and complementary strands. In *Salmonella* phage NINP13076, a majority of the ORFs are located on the complementary strand, while in phage phiSalP219, the distribution of ORFs is nearly equal between the direct and complementary strands. In addition, *Salmonella* phage NINP13076 encodes 21 tRNAs which are situated in the complementary strand, whereas phage phiSalP219 encodes 25 tRNAs present in the direct strand. Phage phiSalP219 contains DNA methyltransferase, which protects phages from bacterial restriction modification systems, and this protein was not found in its close relative phage NINP13076, while it showed 90% similarity with the DNA N-6-adenine methyltransferase of the related *Salmonella* phage GEC_vB_MG.

Notable characteristics of *Salmonella* phage phiSalP219 are the presence of two CDS that encode rIIA lysis inhibitors and rIIB lysis inhibitors. The exact functions of these genes remain to be fully elucidated, but they are speculated to be involved in potentially other cellular processes, including interference within cellular metabolism (Paddison et al., 1998; Alves et al., 2016). In addition, phage phiSalP219 contained several tail proteins, which are hypothesized to be indicators of a broad-spectrum lytic phage (Hooton et al., 2011).

A significant portion of double-stranded DNA (dsDNA) phages utilize lysis cassettes consisting of both holins and endolysins. This ensemble functions by creating openings in the cytoplasmic membrane and dismantling the peptidoglycan layer of the bacterial cell wall. Within the phage phiSalP219, a specific gene, CDS42, has been identified through motif analysis as a member of the lysozyme-like superfamily (cl43803). A BLASTp search of this protein shows 93.64% similarity with the already-reported modular endolysin of *Salmonella* phage PVP-SE1. The gene coding for holins is generally found upstream of the endolysin (Santos et al., 2011). However, analysis of CDS40 and CDS41 failed to show any transmembrane domains, and within the genome, no other ORF showed homology with the holins. In addition, CDS116 shows 72% similarity with the Rz-like spanin of *Escherichia* phage 4MG and is situated far away from other lysin genes. Taking into consideration that these features are not common in dsDNA phages (Whichard et al., 2010; Li et al., 2020), CDS189 encodes cell wall hydrolase; conserved domain analysis of this protein shows that it belongs to the hydrolase 2 superfamily (cl38231).

*Salmonella* phage phiSalP219 contained 25 tRNAs. It has been postulated that the presence of specific tRNA genes confers benefits to phage replication by aligning with codons employed by the phage genome rather than the host genome (Bailly-Bechet et al., 2007). Consequently, the phages that share similar codon usage patterns with their host organisms do not require the retention of tRNA genes and instead rely on the host’s tRNA repertoire (Kunisawa, 2000). Moreover, the phage phiSalP219 genome did not contain any genes related to virulence, antibiotic resistance, or lysogeny. Therefore, this phage is considered to be a good candidate for therapy and biocontrol.

We further examined the efficiency of this phage to reduce *Salmonella* counts in different food matrices. Grygorcewicz et al. (2020) reported that lytic phage ECPS-6 inactivates or decreases the bacterial count of Shiga toxin-producing *E. coli* to an undetected level in the milk sample upon their application at MOI of 5 or 50, but in the experiment with salami and chicken ham, we observed that treatment of the phage phiSalP219 with the contaminated foods did not significantly reduce the viable *Salmonella* count at an MOI of 1,000 (Supplementary Figure S2) at 4°C. Therefore, we used a higher MOI of this phage for *Salmonella* biocontrol in the foods. The reason might be that phages can be very efficient at lower MOIs in liquid because the chances of interaction between bacterial and phage particles are higher than in the case of solids such as the surface of meat. Thus, a higher MOI was taken in the experiment involving meat surfaces. In contaminated salami, phage phiSalP219 administration at MOI = 10,000 reduced viable *Salmonella* counts by 0.661 log10 CFU/g at 4°C and 3.191 log10 CFU/g at 28°C after 48 h of incubation, while phage phiSalP219 application at MOI = 10,000 in chicken ham reduced the viable *Salmonella* count by 0.529 log10 CFU/g at 4°C and 2.046 log10 CFU/g at 28°C after 48 h of incubation. The findings of the present study were comparable to the previous study of Augustine and Bhat (2015), where they reported that phage Φ SP-1 and Φ SP-3 application at high MOI reduced the bacterial count by 3.99 and 3.46 log10 CFU/mL at 28°C compared to the control group on 3 days. They also found that at 4°C, the viable bacterial count dropped by 2.46 and 2.1 log10 CFU/mL at high MOI. In previous studies, it was also reported that on food surfaces, physical interaction between phages and bacteria is limited at low concentrations due to their small size (Guenther et al., 2009; Hagens and Loessner, 2010). However, higher phage concentrations significantly increase the likelihood of phage adsorption on the bacterial cells. Wang et al. (2017) reported that phage application at 10^4^ MOI to the contaminated duck meat reduced the *Salmonella* count by 1.14 log10cfu/cm^2^ at 4°C. Similarly, Thung et al. (2017) reported that phage administration at a high MOI (MOI = 107) reduced the viable *S. enterica* serovar Enteritidis count by 2.0 log10 CFU/g at 4°C. It has also been reported previously that in the final stage of phage infection, bacteria trigger a defense mechanism called abortive infection which prevents the spreading of infection from infected host cells to other host cells to commit suicide before the completion of the phage life cycle (Chaudhary, 2017). Based on our results, we found that higher concentrations of bacteriophage were effective in the biocontrol of *Salmonella* in foods. Phage phiSalP219 was also tested for its ability to eradicate biofilm formed on the borosilicate glass surface by MDR *S. enterica* serovar Typhimurium. Biofilm inhibits the action of antibiotics on the microbial communities by secreting exopolysaccharides a barrier to the action of antibiotics. Phages dissolve the exopolysaccharides and do their bactericidal activities by lysing the bacterial cells. Phage phiSalP219 successfully eradicates the 72-h-old biofilm formed on the glass coverslips, and the degraded biofilm could be seen under FE-SEM in the form of cell debris (Figures 9d–f).

## Data availability statement

The bacterial strains and bacteriophages isolated and used during this study are deposited and available at Bacteriophage Repository, National Centre for Veterinary Type Cultures, ICAR-National Research Centre on Equines, Hisar, Haryana, India (http://ncvtc.org.in/distribution-of-microbes/veterinary-microbes/). The genome of Salmonella phage phiSalP219 has been deposited in the GenBank NCBI database under accession number PP595732.

## Author contributions

AJ: Writing – original draft, Writing – review & editing. RV: Validation, Writing – original draft. MV: Data curation, Writing – review & editing. NV: Formal analysis, Writing – review & editing. BB: Formal analysis, Writing – review & editing. RKV: Formal analysis, Visualization, Writing – review & editing. TA: Conceptualization, Writing – review & editing.

## Footnotes

^1^ http://github.com/brwnj/bcl2fastq

^2^ http://www.bioinformatics.babraham.ac.uk/projects/trimglore/

^3^ http://www.genotypic.co.in/commander/

^4^ http://github.com/rrwick/Porechop

^5^ http://github.com/wdecoster/nanofilt

^6^ https://www.ebi.ac.uk/jdispatcher/psa/emboss_stretcher

^7^ https://rhea.icbm.uni-oldenburg.de/viridic/
