# Supplementary figures and images for: A novel lytic phage infecting MDR Salmonella enterica and its application as effective food biocontrol
Source: Front Microbiol. 2024 Aug 15;15:1387830. doi: 10.3389/fmicb.2024.1387830 (PMC11358711; doi:10.3389/fmicb.2024.1387830)

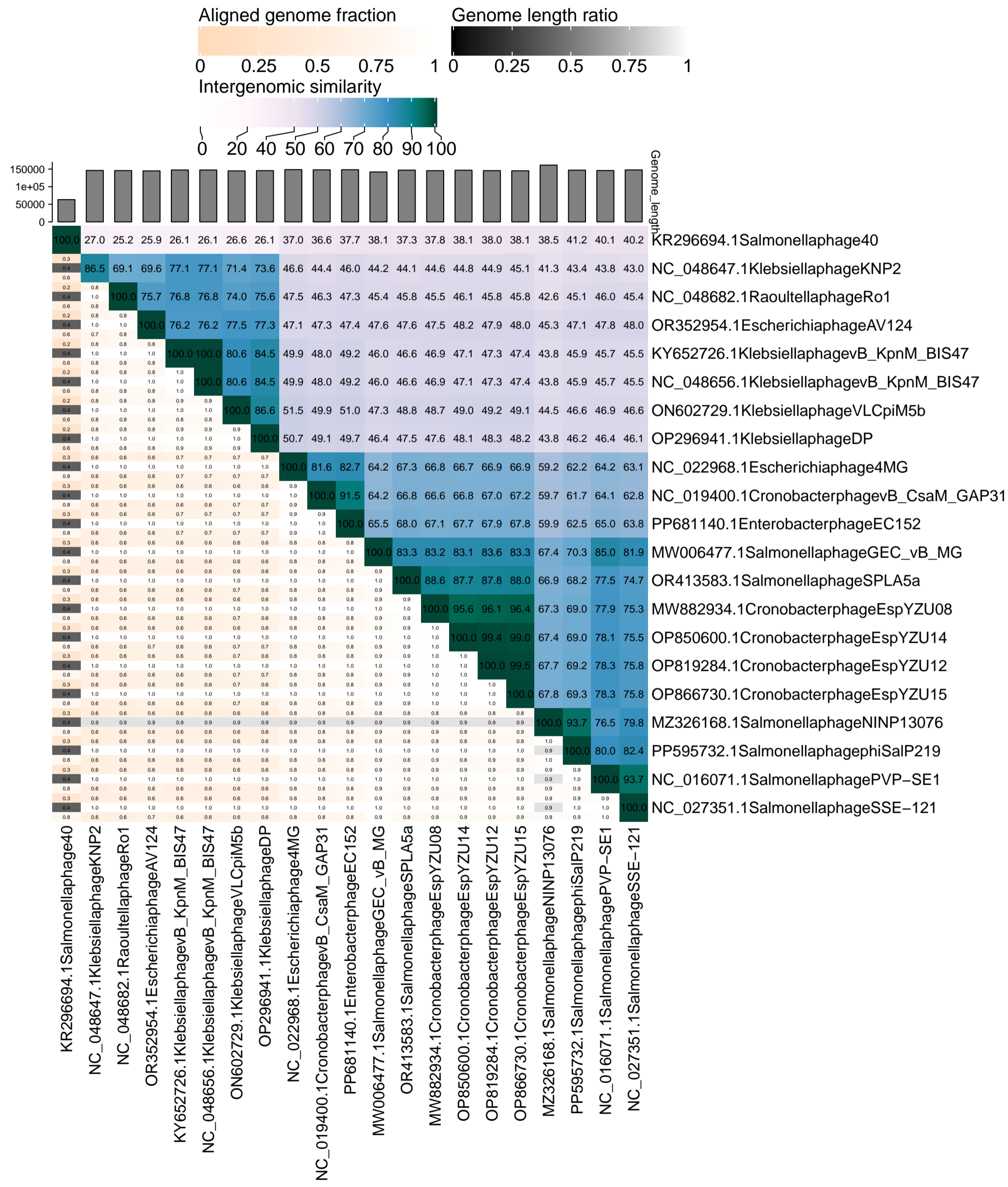

Supplement: Supplementary file 1 [file Image_1.pdf]

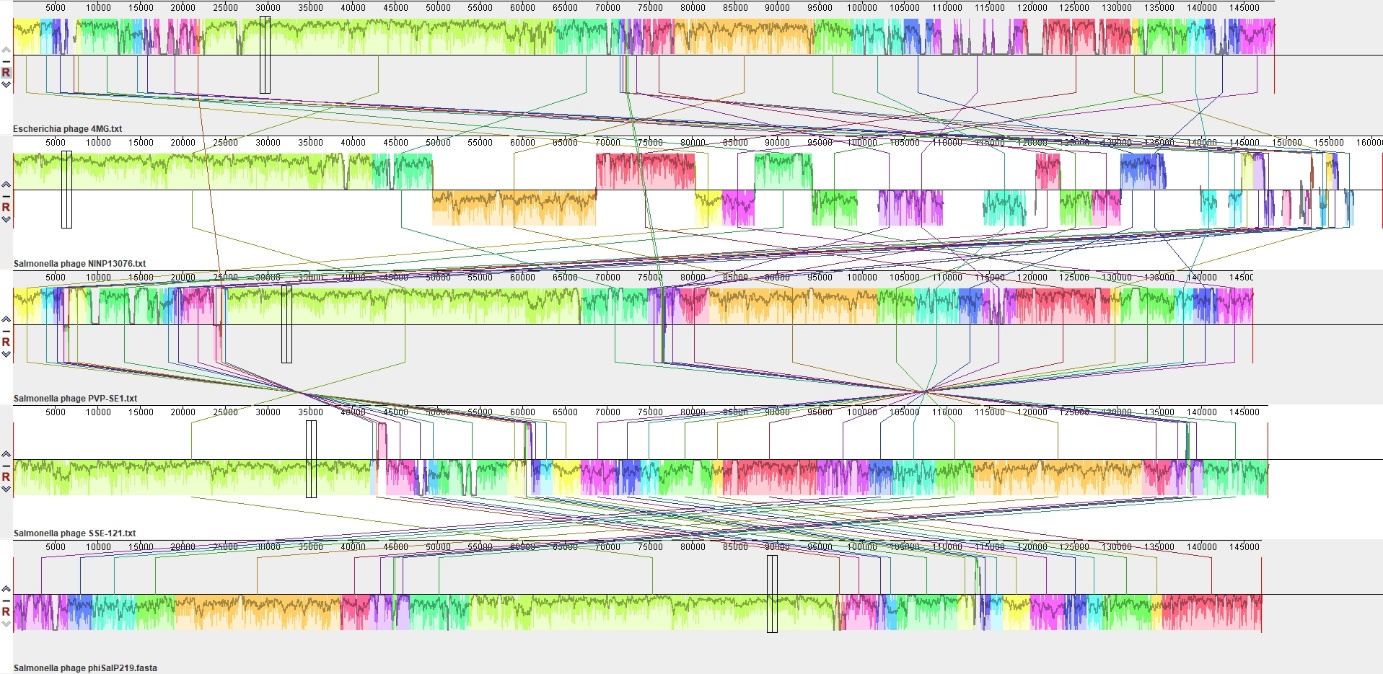

Supplement: Supplementary file 7 [file Table_5.DOCX]
