# Supplementary material for: A novel lytic phage infecting MDR Salmonella enterica and its application as effective food biocontrol
Source: Front Microbiol. 2024 Aug 15;15:1387830. doi: 10.3389/fmicb.2024.1387830 (PMC11358711; doi:10.3389/fmicb.2024.1387830)
Supplement: Supplementary file 2 [file Image_2.pdf]

Supplementary figure:2

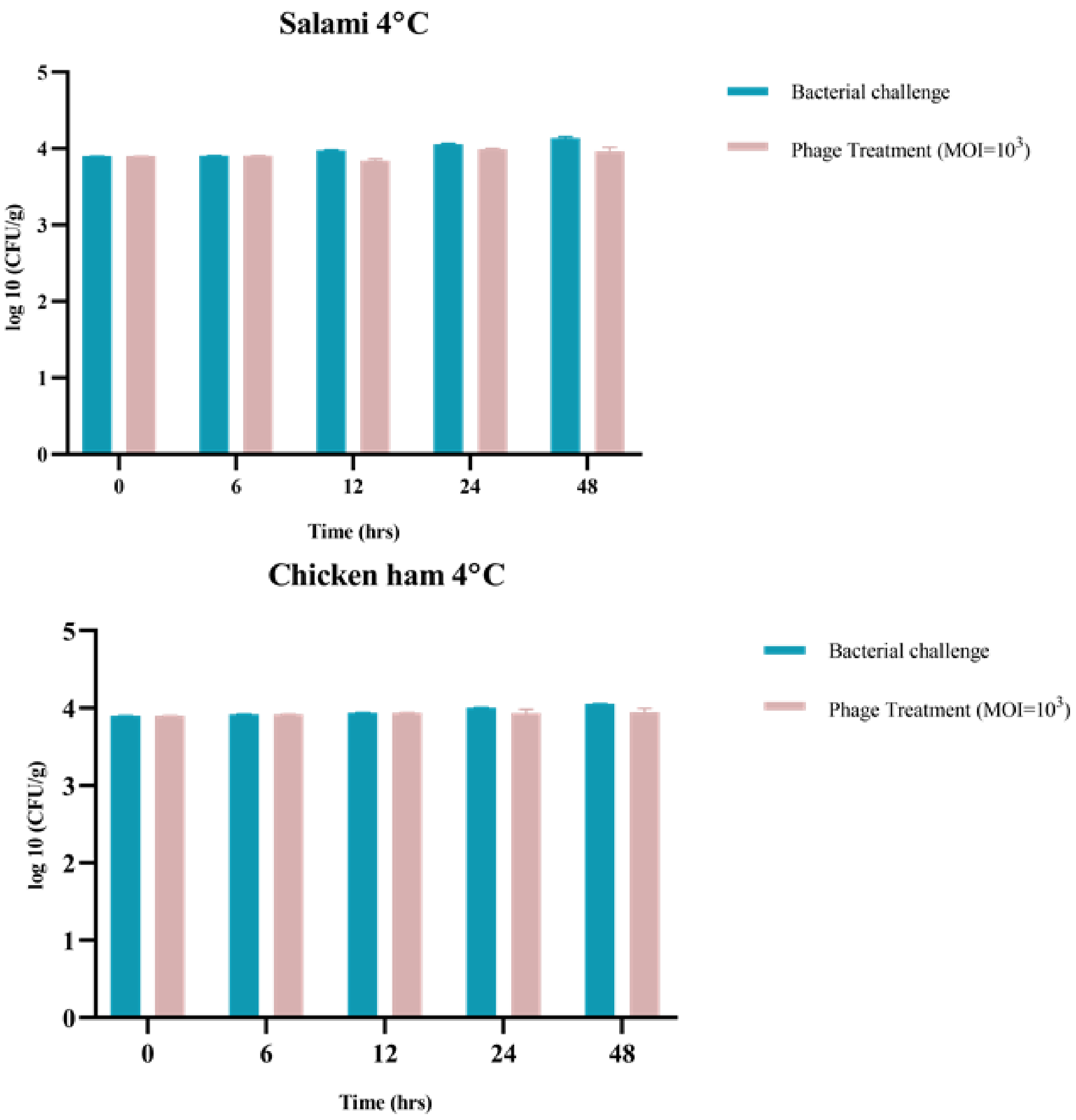

Supplementary figure:2 Biocontrol of *Salmonella* infection in ready to eat chicken ham and salami with phage treatment at 4°C
