# Supplementary material for: A novel lytic phage infecting MDR Salmonella enterica and its application as effective food biocontrol
Source: Front Microbiol. 2024 Aug 15;15:1387830. doi: 10.3389/fmicb.2024.1387830 (PMC11358711; doi:10.3389/fmicb.2024.1387830)
Supplement: Supplementary file 3 [file Table_1.docx]

**Supplementary Table 1: Primary read information of genome sequencing**

| **Illumina Read Statistics** | | | | | | |
| --- | --- | --- | --- | --- | --- | --- |
| **Sample Name** | **Total Raw Reads** | **Sequencing Coverage(X)** | | **Total Processed Reads** | **%Reads Retained** | |
| ***Salmonella* phage phiSalP219** | 5024189 | 10,766.12 | | 5003069 | 99.58 | |
|  | | | | | | |
| **Nanopore Raw Reads Statistics** | | |  | | |  |
| **Sample Name** | | | ***Salmonella* phage phiSalP219** | | |  |
| **number_of_reads** | | | 25656 | | |  |
| **number_of_bases** | | | 19552257 | | |  |
| **median_read_length** | | | 595 | | |  |
| **mean_read_length** | | | 762.1 | | |  |
| **read_length_stdev** | | | 1135.7 | | |  |
| **mean_qual** | | | 11.6 | | |  |
| **median_qual** | | | 11.7 | | |  |
| **Reads >Q7** | | | 25656 (100.0%) 19.6Mb | | |  |
| **Reads >Q10** | | | 21454 (83.6%) 16.5Mb | | |  |
| **Sequencing Coverage** | | | 139.66 | | |  |
