# Supplementary material for: A novel lytic phage infecting MDR Salmonella enterica and its application as effective food biocontrol
Source: Front Microbiol. 2024 Aug 15;15:1387830. doi: 10.3389/fmicb.2024.1387830 (PMC11358711; doi:10.3389/fmicb.2024.1387830)
Supplement: Supplementary file 4 [file Table_2.docx]

**Supplementary Table 2: Genomic feature of Salmonella phage phiSalP219**

| ORF | Strand | Genomic location | Predicted protein | | | | Start codon | Best match on  Blastp (NCBI) | | | | | Additional notes  (based on InterProscan, and CDD) |
| --- | --- | --- | --- | --- | --- | --- | --- | --- | --- | --- | --- | --- | --- |
|  |  |  | **Size (bp)** | **Size (aa)** | **MW**  **(kDa)** | **IP** |  | **Annotation (predicted function)** | **Identity (%)** | **Coverage (%)** | **E-value** | **Blastp hit** |  |
| 1 | - | 1,404..1,520 | 117 | 38 | 4.2 | 9.4 | ATG | Hypothetical protein | - | - | - | - | - |
| 2 | - | 1,659..2,023 | 381 | 126 | 14.8 | 7.0 | ATG | Hypothetical protein (Salmonella phage NINP13076) | 100 | 88 | 2e-77 | [QXL90469.1](https://www.ncbi.nlm.nih.gov/protein/QXL90469.1?report=genbank&log$=prottop&blast_rank=2&RID=AFVCVEH3013) | - |
| 3 | - | 2,032..2,172 | 141 | 46 | 5.6 | 5.1 | ATG | Hypothetical protein (Salmonella phage NINP13076) | 100 | 89 | 3e-21 | [QXL90496.1](https://www.ncbi.nlm.nih.gov/protein/QXL90469.1?report=genbank&log$=prottop&blast_rank=2&RID=AFS40A1C016) | - |
| 4 | - | 2,169..2,390 | 222 | 73 | 7.7 | 4.9 | ATG | Hypothetical protein (Salmonella phage NINP13076) | 100 | 100 | 1e-47 | [QXL90470.1](https://www.ncbi.nlm.nih.gov/protein/QXL90470.1?report=genbank&log$=prottop&blast_rank=1&RID=AFUC7FDY013) | - |
| 5 | - | 2,399..2,584 | 186 | 61 | 6.9 | 7.6 | ATG | Hypothetical protein (Salmonella phage NINP13076) | 100 | 100 | 4e-34 | [QXL90471.1](https://www.ncbi.nlm.nih.gov/protein/QXL90471.1?report=genbank&log$=prottop&blast_rank=1&RID=AFWM4U64013) | - |
| 6 | - | 2,581..2,808 | 228 | 75 | 8.8 | 5.6 | ATG | Hypothetical protein (Salmonella phage NINP13076) | 92 | 100 | 6e-43 | [QXL90493.1](https://www.ncbi.nlm.nih.gov/protein/QXL90493.1?report=genbank&log$=prottop&blast_rank=2&RID=AFXVG9JE016) | - |
| 7 | - | 2,805..2,918 | 114 | 37 | 4.1 | 9.2 | ATG | Hypothetical protein (Salmonella phage SSE121) | 87.88 | 89 | 2e-12 | [YP_009148943.1](https://www.ncbi.nlm.nih.gov/protein/YP_009148943.1?report=genbank&log$=prottop&blast_rank=1&RID=AFYG27ZP013) | - |
| 8 | - | 3,011..3,334 | 324 | 107 | 12.6 | 8.7 | ATG | Hypothetical protein (Salmonella phage NINP13076) | 99.07 | 100 | 1e-72 | [QXL90474.1](https://www.ncbi.nlm.nih.gov/protein/QXL90474.1?report=genbank&log$=prottop&blast_rank=1&RID=AFYSE3U2016) | - |
| 9 | - | 3,349..3,594 | 246 | 81 | 9.7 | 5.2 | ATG | Hypothetical protein (Salmonella phage NINP13076) | 98.77 | 100 | 3e-50 | [QXL90475.1](https://www.ncbi.nlm.nih.gov/protein/QXL90475.1?report=genbank&log$=prottop&blast_rank=2&RID=AFZ44YF9013) | - |
| 10 | - | 3,596..3,790 | 195 | 64 | 7.1 | 10.38 | ATG | Hypothetical protein (Salmonella phage SSE121) | 100 | 100 | 9e-51 | [YP_009148945.1](https://www.ncbi.nlm.nih.gov/protein/YP_009148945.1?report=genbank&log$=prottop&blast_rank=1&RID=AG0BXHWM013) | - |
| 11 | - | 3,787..4,023 | 237 | 78 | 9.05 | 7.8 | ATG | Hypothetical protein (Salmonella phage SSE121) | 100 | 100 | 2e-49 | [YP_009148947.1](https://www.ncbi.nlm.nih.gov/protein/YP_009148947.1?report=genbank&log$=prottop&blast_rank=1&RID=AG0WDZ8D01N) | - |
| 12 | - | 4,026..4,232 | 207 | 68 | 7.8 | 6.5 | ATG | Hypothetical protein (Salmonella phage SSE121) | 98.33 | 88 | 4e-36 | [YP_009148948.1](https://www.ncbi.nlm.nih.gov/protein/YP_009148948.1?report=genbank&log$=prottop&blast_rank=1&RID=AG11DWWS01N) | - |
| 13 | + | 4,329..5,564 | 1236 | 411 | 45.95 | 9.8 | TTG | Hypothetical protein (Klebsiella phage KPP-1) | 42.47 | 100 | 3e-64 | [QKE60599.1](https://www.ncbi.nlm.nih.gov/protein/QKE60599.1?report=genbank&log$=prottop&blast_rank=1&RID=AG1A0ZCW013) | - |
| 14 | + | 5,580..5,891 | 312 | 103 | 11.8 | 4.8 | ATG | Hypothetical protein (Salmonella phage NINP13076) | 100 | 100 | 9e-69 | [QXL90481.1](https://www.ncbi.nlm.nih.gov/protein/QXL90481.1?report=genbank&log$=prottop&blast_rank=1&RID=AG1RA38101N) | - |
| 15 | - | 5,923..6,417 | 495 | 164 | 18.3 | 6.8 | ATG | Hypothetical protein (Salmonella phage SSE121) | 100 | 100 | 2e-117 | [YP_009148951.1](https://www.ncbi.nlm.nih.gov/protein/YP_009148951.1?report=genbank&log$=prottop&blast_rank=1&RID=AG20M3PW013) | - |
| 16 | - | 6,402..6,797 | 396 | 131 | 15.7 | 9.75 | GTG | Hypothetical protein (Salmonella phage SSE121) | 100 | 93 | 6e-84 | [YP_009148952.1](https://www.ncbi.nlm.nih.gov/protein/YP_009148952.1?report=genbank&log$=prottop&blast_rank=1&RID=AGBV28C0016) | - |
| 17 | - | 6,807..7,364 | 558 | 185 | 21.8 | 8.81 | ATG | Hypothetical protein (Salmonella phage NINP13076) | 100 | 100 | 1e-134 | [QXL90542.1](https://www.ncbi.nlm.nih.gov/protein/QXL90542.1?report=genbank&log$=prottop&blast_rank=1&RID=AGCEHFWE016) | - |
| 18 | - | 7,377..7,607 | 231 | 76 | 8.04 | 8.14 | ATG | Hypothetical protein (Salmonella phage SSE121) | 100 | 100 | 7e-47 | [YP_009148954.1](https://www.ncbi.nlm.nih.gov/protein/YP_009148954.1?report=genbank&log$=prottop&blast_rank=1&RID=AGDHP7TX013) | - |
| 19 | - | 7,604..7,774 | 171 | 56 | 6.926 | 4.28 | ATG | Hypothetical protein (Salmonella phage SSE121) | 100 | 100 | 8e-31 | [YP_009148955.1](https://www.ncbi.nlm.nih.gov/protein/YP_009148955.1?report=genbank&log$=prottop&blast_rank=1&RID=AGDWH0RP013) | - |
| 20 | - | 7,764..7,973 | 210 | 69 | 7.9 | 7.91 | ATG | Hypothetical protein (Salmonella phage NINP13076) | 100 | 100 | 6e-43 | [QXL90539.1](https://www.ncbi.nlm.nih.gov/protein/QXL90539.1?report=genbank&log$=prottop&blast_rank=1&RID=AGEK51UA016) | - |
| 21 | - | 7,966..9,027 | 1062 | 353 | 41.4 | 5.42 | ATG | Nucleotidyltransferase (Salmonella phage PVPSE1) | 93.47 | 99 | 0 | [YP_004893973.1](https://www.ncbi.nlm.nih.gov/protein/YP_004893973.1?report=genbank&log$=prottop&blast_rank=2&RID=AHHZ44SM013) | InterPro hit: IPR018775: RNA repair pathway DNA polymerase beta family (PF10127), Nuc-transf super family (c10417) |
| 22 | - | 9,036..9,398 | 363 | 120 | 14.03 | 6.9 | GTG | Hypothetical protein (Salmonella phage PVPSE1) | 87.5 | 100 | 7e-73 | [YP_004893972.1](https://www.ncbi.nlm.nih.gov/protein/YP_004893972.1?report=genbank&log$=prottop&blast_rank=2&RID=AHK1FFU7016) | -- |
| 23 | - | 9,400..9735 | 336 | 111 | 12.2 | 9.9 | ATG | Hypothetical protein (Salmonella phage NINP13076) | 100 | 100 | 6e-72 | [QXL90498.1](https://www.ncbi.nlm.nih.gov/protein/QXL90498.1?report=genbank&log$=prottop&blast_rank=1&RID=AHKECVPA01N) | - |
| 24 | - | 9,735..9917 | 183 | 60 | 6.8 | 4.8 | ATG | Hypothetical protein (Salmonella phage NINP13076) | 100 | 100 | 2e-34 | [QXL90499.1](https://www.ncbi.nlm.nih.gov/protein/QXL90499.1?report=genbank&log$=prottop&blast_rank=1&RID=AHKNW51D013) | - |
| 25 | - | 9,919..10,410 | 492 | 163 | 18.6 | 5.7 | ATG | Hypothetical protein (Salmonella phage NINP13076) | 100 | 100 | 2e-118 | [QXL90500.1](https://www.ncbi.nlm.nih.gov/protein/QXL90500.1?report=genbank&log$=prottop&blast_rank=1&RID=AHM2Z9ER016) | - |
| 26 | - | 10,407..10,628 | 222 | 73 | 8.3 | 7.5 | ATG | Hypothetical protein (Salmonella phage NINP13076) | 100 | 100 | 8e-44 | [QXL90501.1](https://www.ncbi.nlm.nih.gov/protein/QXL90501.1?report=genbank&log$=prottop&blast_rank=1&RID=AHM7X2AC013) | - |
| 27 | - | 10,628..10,843 | 216 | 71 | 8.1 | 9.3 | ATG | Hypothetical protein (Salmonella phage NINP13076) | 100 | 100 | 7e-43 | [QXL90502.1](https://www.ncbi.nlm.nih.gov/protein/QXL90502.1?report=genbank&log$=prottop&blast_rank=1&RID=AHV7JYSN01N) | - |
| 28 | - | 10,852..11058 | 207 | 68 | 8.1 | 9.2 | ATG | Hypothetical protein (Salmonella phage NINP13076) | 100 | 100 | 6e-43 | [QXL90503.1](https://www.ncbi.nlm.nih.gov/protein/QXL90503.1?report=genbank&log$=prottop&blast_rank=1&RID=AHVT9D20013) | - |
| 29 | - | 11,068..12,321 | 1254 | 417 | 46.5 | 6.1 | ATG | CCA tRNA nucleotidyltransferase (Salmonella phage NINP13076) | 1001 | 100 | 0 | [QXL90504.1](https://www.ncbi.nlm.nih.gov/protein/QXL90504.1?report=genbank&log$=prottop&blast_rank=1&RID=AHX2EGEM013) | tRNA nucleotidyltransferase, proteobacteria (IPR012006), Poly A polymerase HD domain (PF01743), tRNA nucleotidyltransferase/ poly(A) polymerase, RNA and SrmB-binding domain (IPR032828), CCA superfamily ([cl42923](https://www.ncbi.nlm.nih.gov/Structure/cdd/cddsrv.cgi?ascbin=8&maxaln=10&seltype=2&uid=cl42923)) |
| 30 | - | 12,331..12,597 | 267 | 88 | 10.3 | 6.5 | ATG | Hypothetical protein (Salmonella phage NINP13076) | 100 | 100 | 2e-57 | [QXL90505.1](https://www.ncbi.nlm.nih.gov/protein/QXL90505.1?report=genbank&log$=prottop&blast_rank=1&RID=AHZW9WPX013) | - |
| 31 | - | 12,594..  12,902 | 309 | 102 | 11.08 | 4.9 | ATG | Hypothetical protein (Salmonella phage NINP13076) | 100 | 100 | 2e-68 | [QXL90506.1](https://www.ncbi.nlm.nih.gov/protein/QXL90506.1?report=genbank&log$=prottop&blast_rank=1&RID=AJ6X19K4016) | - |
| 32 | - | 12,892..  13,143 | 252 | 83 | 9.2 | 5 | ATG | Hypothetical protein (Salmonella phage NINP13076) | 100 | 100 | 1e-53 | [QXL90507.1](https://www.ncbi.nlm.nih.gov/protein/QXL90507.1?report=genbank&log$=prottop&blast_rank=1&RID=AJ76BKCD016) | - |
| 33 | - | 13,140..  13,292 | 153 | 50 | 6.07 | 4.9 | ATG | Hypothetical protein (Salmonella phage NINP13076) | 100 | 100 | 9e-29 | [QXL90508.1](https://www.ncbi.nlm.nih.gov/protein/QXL90508.1?report=genbank&log$=prottop&blast_rank=1&RID=AJ7D3BGR013) | - |
| 34 | - | 13,289..  13,501 | 213 | 70 | 8.2 | 4.8 | ATG | Hypothetical protein (Salmonella phage NINP13076) | 100 | 100 | 8e-44 | [QXL90509.1](https://www.ncbi.nlm.nih.gov/protein/QXL90509.1?report=genbank&log$=prottop&blast_rank=1&RID=AJ7KN22801N) | PS51257 Prokaryotic membrane lipoprotein lipid attachment site profile |
| 35 | - | 13,503..  13,772 | 270 | 89 | 10.8 | 9.1 | ATG | Hypothetical protein (Salmonella phage NINP13076) | 100 | 100 | 2e-58 | [QXL90510.1](https://www.ncbi.nlm.nih.gov/protein/QXL90510.1?report=genbank&log$=prottop&blast_rank=1&RID=AJ82HZDW013) | - |
| 36 | - | 13,973..  14,653 | 681 | 226 | 25.8 | 6.02 | ATG | DNA N-6-adenine methyltransferase (Salmonella phage GEC_vB_MG) | 90.27 | 100 | 3e-152 | [QPI14708.1](https://www.ncbi.nlm.nih.gov/protein/QPI14708.1?report=genbank&log$=prottop&blast_rank=1&RID=AJ8MCGX6013) | - |
| 37 | - | 14,653..  15,573 | 921 | 306 | 34 | 4.71 | ATG | ATP dependent protease (Escherichia phage 4MG) | 80.46 | 99 | 0 | [YP_008857360.1](https://www.ncbi.nlm.nih.gov/protein/YP_008857360.1?report=genbank&log$=prottop&blast_rank=1&RID=AJ936KU0016) | [ClpP/crotonase-like domain superfamily](https://www.ebi.ac.uk/interpro/entry/InterPro/IPR029045/) (IPR029045), crotonase-like super family ([cl23717](https://www.ncbi.nlm.nih.gov/Structure/cdd/cddsrv.cgi?ascbin=8&maxaln=10&seltype=2&uid=cl23717)) |
| 38 | - | 15,575..  16,057 | 483 | 160 | 18.7 | 5.4 | ATG | Hypothetical protein (Salmonella phage NINP13076) | 100 | 100 | 7e-115 | [QXL90523.1](https://www.ncbi.nlm.nih.gov/protein/QXL90523.1?report=genbank&log$=prottop&blast_rank=1&RID=AJA9X24F013) | - |
| 39 | - | 16,065..  16331 | 267 | 88 | 18.7 | 5.4 | GTG | Hypothetical protein (Salmonella phage NINP13076) | 100 | 100 | 9e-56 | [QXL90524.1](https://www.ncbi.nlm.nih.gov/protein/QXL90524.1?report=genbank&log$=prottop&blast_rank=1&RID=AJAMCZDW016) | - |
| 40 | - | 16,360..  16,992 | 633 | 210 | 23.9 | 6.54 | ATG | Hypothetical protein (Salmonella phage NINP13076) | 100 | 99.05 | 7e-153 | [QXL90525.1](https://www.ncbi.nlm.nih.gov/protein/QXL90525.1?report=genbank&log$=prottop&blast_rank=1&RID=AJB53AVU013) | - |
| 41 | - | 17,003..  17,791 | 789 | 262 | 29.2 | 8.7 | ATG | PhoH-like protein (Salmonella phage NINP13076) | 100 | 100 | 0 | [QXL90526.1](https://www.ncbi.nlm.nih.gov/protein/QXL90526.1?report=genbank&log$=prottop&blast_rank=1&RID=AJDRWP6V013) | PhoH-like protein (IPR003714),  P-loop_NTPase super family ([cl38936](https://www.ncbi.nlm.nih.gov/Structure/cdd/cddsrv.cgi?ascbin=8&maxaln=10&seltype=2&uid=cl38936)) |
| 42 | - | 17,803..  18,513 | 711 | 236 | 25.2 | 9.1 | ATG | Endolysin (Salmonella phage NINP13076) | 100 | 100 | 1e-177 | [QXL90527.1](https://www.ncbi.nlm.nih.gov/protein/QXL90527.1?report=genbank&log$=prottop&blast_rank=1&RID=AJERW25J016) | Lysozyme-like domain superfamily (IPR023346), Glycoside hydrolase, family 19, catalytic (IPR000726), COG3179 super family ([cl43803](https://www.ncbi.nlm.nih.gov/Structure/cdd/cddsrv.cgi?ascbin=8&maxaln=10&seltype=2&uid=cl43803)) |
| 43 | - | 18,532..  18,957 | 426 | 141 | 16 | 4.8 | ATG | Hypothetical protein (Salmonella phage NINP13076) | 100 | 100 | 3e-96 | [QXL90528.1](https://www.ncbi.nlm.nih.gov/protein/QXL90528.1?report=genbank&log$=prottop&blast_rank=1&RID=AJFBGPVE013) | - |
| 44 | - | 18,966..  19,106 | 141 | 46 | 5.3 | 10.1 | ATG | Hypothetical protein (Escherichia phage 4MG) | 95.65 | 100 | 9e-21 | [YP_008857353.1](https://www.ncbi.nlm.nih.gov/protein/YP_008857353.1?report=genbank&log$=prottop&blast_rank=1&RID=AJFD1D5C013) | - |
| 45 | - | 19,107..  19,580 | 474 | 157 | 18 | 5.7 | ATG | Ribonucleotide reductase of class III (anaerobic), activating protein (Salmonella phage NINP13076) | 100 | 100 | 5e-112 | [QXL90351.1](https://www.ncbi.nlm.nih.gov/protein/QXL90351.1?report=genbank&log$=prottop&blast_rank=1&RID=AJFPNK71013) | NrdG super family ([cl37123](https://www.ncbi.nlm.nih.gov/Structure/cdd/cddsrv.cgi?ascbin=8&maxaln=10&seltype=2&uid=cl37123)), [Ribonucleoside-triphosphate reductase activating, anaerobic](https://www.ebi.ac.uk/interpro/entry/interpro/IPR012837)(IPR012837) |
| 46 | - | 19,577..  21,682 | 2106 | 701 | 78.5 | 6.2 | GTG | Ribonucleotide reductase of class III (anaerobic), large subunit (Salmonella phage NINP13076) | 99.86 | 100 | 0 | [QXL90352.1](https://www.ncbi.nlm.nih.gov/protein/QXL90352.1?report=genbank&log$=prottop&blast_rank=1&RID=AJFSWJ6C013) | [Ribonucleoside-triphosphate reductase, anaerobic](https://www.ebi.ac.uk/interpro/entry/interpro/IPR012833)(IPR012833), Anaerobic ribonucleoside-triphosphate reductase (PF13597), Ribonucleotide reductase and Pyruvate formate lyase ([cl38938](https://www.ncbi.nlm.nih.gov/Structure/cdd/cddsrv.cgi?ascbin=8&maxaln=10&seltype=2&uid=cl38938)) |
| 47 | - | 21,695..  21,958 | 264 | 87 | 10 | 7.6 | ATG | Hypothetical protein (Salmonella phage NINP13076) | 100 | 100 | 2e-57 | [QXL90353.1](https://www.ncbi.nlm.nih.gov/protein/QXL90353.1?report=genbank&log$=prottop&blast_rank=1&RID=AJGU2EBE016) | [Thioredoxin-like superfamily](https://www.ebi.ac.uk/interpro/entry/InterPro/IPR036249/) (IPR036249), Glutaredoxin (PF00462), Thioredoxin_like super family ([cl00388](https://www.ncbi.nlm.nih.gov/Structure/cdd/cddsrv.cgi?ascbin=8&maxaln=10&seltype=2&uid=cl00388)) |
| 48 | - | 21,958..  22,299 | 342 | 113 | 13 | 7.8 | ATG | Hypothetical protein (Salmonella phage NINP13076) | 100 | 100 | 7e-77 | [QXL90354.1](https://www.ncbi.nlm.nih.gov/protein/QXL90354.1?report=genbank&log$=prottop&blast_rank=1&RID=AJHDGN6N013) | - |
| 49 | - | 22,308..  23,396 | 1089 | 362 | 41.7 | 41.7 | ATG | Ribonucleotide reductase of class Ia (aerobic), beta subunit (Salmonella phage NINP13076) | 100 | 100 | 0 | [QXL90355.1](https://www.ncbi.nlm.nih.gov/protein/QXL90355.1?report=genbank&log$=prottop&blast_rank=1&RID=AJHTREVU013) | Ribonucleotide Reductase, R2/beta subunit, ferritin-like diiron-binding domain ([cd01049](https://www.ncbi.nlm.nih.gov/Structure/cdd/cddsrv.cgi?ascbin=8&maxaln=10&seltype=2&uid=cd01049)), [Ribonucleotide reductase small subunit family](https://www.ebi.ac.uk/interpro/entry/interpro/IPR000358)(IPR000358), Ferittin like superfamily (IPR009078) |
| 50 | - | 23,434..  25,731 | 2298 | 765 | 86.6 | 5.6 | TTG | Ribonucleotide reductase of class Ia (aerobic), beta subunit (Salmonella phage NINP13076) | 99.87 | 100 | 0 | [QXL90356.1](https://www.ncbi.nlm.nih.gov/protein/QXL90356.1?report=genbank&log$=prottop&blast_rank=1&RID=AJJGBPV5013) | Ribonucleoside-diphosphate reductase large subunit (IPR039718), nrdA super family ([cl33688](https://www.ncbi.nlm.nih.gov/Structure/cdd/cddsrv.cgi?ascbin=8&maxaln=10&seltype=2&uid=cl33688)) |
| 51 | - | 25,741..  25,971 | 231 | 76 | 9.1 | 4.4 | ATG | Hypothetical protein (Salmonella phage NINP13076) | 100 | 100 | 4e-48 | [QXL90357.1](https://www.ncbi.nlm.nih.gov/protein/QXL90357.1?report=genbank&log$=prottop&blast_rank=1&RID=AM5XP0D1013) | - |
| 52 | - | 25,968..  26,447 | 480 | 159 | 18 | 5.2 | ATG | Hypothetical protein (Salmonella phage NINP13076) | 100 | 100 | 1e-112 | [QXL90358.1](https://www.ncbi.nlm.nih.gov/protein/QXL90358.1?report=genbank&log$=prottop&blast_rank=1&RID=AM68MGMX01N) | - |
| 53 | - | 26,542..  26,751 | 210 | 69 | 7.9 | 5.2 | TTG | HNH homing endonuclease (Salmonella phage GEC_vB_MG) | 98.55 | 100 | 8e-42 | [QPI14691.1](https://www.ncbi.nlm.nih.gov/protein/QPI14691.1?report=genbank&log$=prottop&blast_rank=1&RID=AM6KEMUU013) | HNHc super family ([cl00083](https://www.ncbi.nlm.nih.gov/Structure/cdd/cddsrv.cgi?ascbin=8&maxaln=10&seltype=2&uid=cl00083)), HNH nuclease (IPR003615), HNH endonuclease (PF13392) |
| 54 | - | 26,802..  26,921 | 120 | 39 | 4.3 | 10 | ATG | Hypothetical protein | - | - | - | - | - |
| 55 | - | 26,960..  27937 | 978 | 325 | 36.9 | 5.7 | ATG | FAD-dependent thymidylate synthase (Salmonella phage PVPSE1) | 100 | 100 | 0 | [YP_004893940.1](https://www.ncbi.nlm.nih.gov/protein/YP_004893940.1?report=genbank&log$=prottop&blast_rank=1&RID=AM844XAG016) | [Thymidylate synthase ThyX](https://www.ebi.ac.uk/interpro/entry/interpro/IPR003669)(IPR003669), Thymidylate synthase complementing protein (PF02511), ThyX super family ([cl42216](https://www.ncbi.nlm.nih.gov/Structure/cdd/cddsrv.cgi?ascbin=8&maxaln=10&seltype=2&uid=cl42216)) |
| 56 | - | 27,937..  28,257 | 321 | 106 | 11.9 | 4.6 | ATG | Hypothetical protein (Salmonella phage PVPSE1) | 100 | 100 | 4e-68 | [YP_004893939.1](https://www.ncbi.nlm.nih.gov/protein/YP_004893939.1?report=genbank&log$=prottop&blast_rank=1&RID=AM8M5FZ6013) | - |
| 57 | - | 28,254..  28,448 | 195 | 64 | 7.4 | 9.1 | ATG | Hypothetical protein (Salmonella phage NINP13076) | 100 | 100 | 2e-39 | [QXL90362.1](https://www.ncbi.nlm.nih.gov/protein/QXL90362.1?report=genbank&log$=prottop&blast_rank=1&RID=AM8VAU4K013) | - |
| 58 | - | 28,448..  29050 | 603 | 200 | 22.9 | 5.3 | ATG | Hypothetical protein (Salmonella phage NINP13076) | 99 | 100 | 9e-54 | [QXL90363.1](https://www.ncbi.nlm.nih.gov/protein/QXL90363.1?report=genbank&log$=prottop&blast_rank=1&RID=AM7NT4DK01N) | YorC super family, 5'(3')-deoxyribonucleotidase ([cl42549](https://www.ncbi.nlm.nih.gov/Structure/cdd/cddsrv.cgi?ascbin=8&maxaln=10&seltype=2&uid=cl42549)), HAD superfamily (IPR023214) |
| 59 | - | 29,043..  29,648 | 606 | 201 | 22.9 | 5.0 | ATG | Hypothetical protein (Salmonella phage NINP13076) | 99.5 | 100 | 3e-146 | [QXL90364.1](https://www.ncbi.nlm.nih.gov/protein/QXL90364.1?report=genbank&log$=prottop&blast_rank=1&RID=AM91HWDG013) | - |
| 60 | - | 29,648..  29,833 | 186 | 61 | 7.1 | 4.2 | ATG | Hypothetical protein (Salmonella phage NINP13076) | 100 | 100 | 2e-35 | [QXL90365.1](https://www.ncbi.nlm.nih.gov/protein/QXL90365.1?report=genbank&log$=prottop&blast_rank=1&RID=AM98RCWS016) | - |
| 61 | - | 29,865..  30,839 | 975 | 324 | 37.6 | 6.3 | ATG | Hypothetical protein (Salmonella phage NINP13076) | 99.38 | 100 | 0 | [QXL90366.1](https://www.ncbi.nlm.nih.gov/protein/QXL90366.1?report=genbank&log$=prottop&blast_rank=1&RID=ASAS9WUH01N) | Ribonuclease H superfamily (IPR036397), DnaQ_like_exo super family  ([cl10012](https://www.ncbi.nlm.nih.gov/Structure/cdd/cddsrv.cgi?ascbin=8&maxaln=10&seltype=2&uid=cl10012)) |
| 62 | - | 30,839..  30,976 | 138 | 45 | 5.4 | 4.3 | ATG | Hypothetical protein (Salmonella phage SSE121) | 95.36 | 100 | 9e-23 | [YP_009148996.1](https://www.ncbi.nlm.nih.gov/protein/YP_009148996.1?report=genbank&log$=prottop&blast_rank=1&RID=ASBB5301013) | - |
| 63 | - | 30,996..  31,574 | 579 | 192 | 21.9 | 8.3 | GTG | Hypothetical protein (Salmonella phage 39) | 98.96 | 100 | 5e-139 | [AKJ73579.1](https://www.ncbi.nlm.nih.gov/protein/AKJ73579.1?report=genbank&log$=prottop&blast_rank=1&RID=ASC0KN0U013) | 49 super family; recombination endonuclease VII ([cl40766](https://www.ncbi.nlm.nih.gov/Structure/cdd/cddsrv.cgi?ascbin=8&maxaln=10&seltype=2&uid=cl40766)), [Recombination endonuclease VII](https://www.ebi.ac.uk/interpro/entry/InterPro/IPR004211/) (IPR004211) |
| 64 | - | 31,571..  32,185 | 615 | 204 | 22.6 | 8.6 | ATG | Hypothetical protein (Salmonella phage NINP13076) | 100 | 100 | 3e-48 | [QXL90369.1](https://www.ncbi.nlm.nih.gov/protein/QXL90369.1?report=genbank&log$=prottop&blast_rank=1&RID=ASCD9S4J016) | Ribonuclease H superfamily (IPR03697) |
| 65 | - | 32,166..  32,483 | 318 | 105 | 11.6 | 4.0 | ATG | Hypothetical protein (Salmonella phage NINP13076) | 98.95 | 90 | 3e-60 | [QXL90370.1](https://www.ncbi.nlm.nih.gov/protein/QXL90370.1?report=genbank&log$=prottop&blast_rank=1&RID=ATFXZUZ4013) | - |
| 66 | - | 32,480..  33,601 | 1122 | 373 | 42.6 | 6.6 | GTG | Hypothetical protein (Salmonella phage NINP13076) | 99.46 | 100 | 0 | [QXL90371.1](https://www.ncbi.nlm.nih.gov/protein/QXL90371.1?report=genbank&log$=prottop&blast_rank=1&RID=AUCAEFRD013) | PIN-like domain superfamily (IPR029060), PHA00439 super family ([cl33674](https://www.ncbi.nlm.nih.gov/Structure/cdd/cddsrv.cgi?ascbin=8&maxaln=10&seltype=2&uid=cl33674)) |
| 67 | - | 33,610..  34,047 | 438 | 145 | 16.6 | 8.8 | TTG | Hypothetical protein (Salmonella phage NINP13076) | 98.62 | 100 | 2e-102 | [QXL90372.1](https://www.ncbi.nlm.nih.gov/protein/QXL90372.1?report=genbank&log$=prottop&blast_rank=1&RID=AUENSVTY013) | 5-methylcytosine-specific restriction endonuclease McrA [Defense mechanisms] [(cl43413](https://www.ncbi.nlm.nih.gov/Structure/cdd/cddsrv.cgi?ascbin=8&maxaln=10&seltype=2&uid=cl43413)), HNH nuclease (IPR003615) |
| 68 | - | 34,044..  34,556 | 513 | 170 | 19.7 | 9 | ATG | Hypothetical protein (Salmonella phage NINP13076) | 99.41 | 100 | 5e-123 | [QXL90373.1](https://www.ncbi.nlm.nih.gov/protein/QXL90373.1?report=genbank&log$=prottop&blast_rank=1&RID=AUTT5F0D013) | NADAR super family ([cl21532](https://www.ncbi.nlm.nih.gov/Structure/cdd/cddsrv.cgi?ascbin=8&maxaln=10&seltype=2&uid=cl21532)) |
| 69 | - | 34,565..  34,759 | 195 | 64 | 6.9 | 7.7 | ATG | Hypothetical protein (Salmonella phage NINP13076) | 98.44 | 100 | 3e-38 | [QXL90374.1](https://www.ncbi.nlm.nih.gov/protein/QXL90374.1?report=genbank&log$=prottop&blast_rank=1&RID=AUU3EAPX016) | - |
| 70 | - | 34,823..  35,713 | 891 | 296 | 32.6 | 8.3 | ATG | Hypothetical protein (Salmonella phage NINP13076) | 99.66 | 100 | 0 | [QXL90375.1](https://www.ncbi.nlm.nih.gov/protein/QXL90375.1?report=genbank&log$=prottop&blast_rank=1&RID=AUUCAK0B013) | SPFH_like super family ([cl19107](https://www.ncbi.nlm.nih.gov/Structure/cdd/cddsrv.cgi?ascbin=8&maxaln=10&seltype=2&uid=cl19107)), Band 7 domain (IPR001107 |
| 71 | - | 35,706..  35,870 | 165 | 54 | 6.2 | 8.9 | ATG | Hypothetical protein (Salmonella phage NINP13076) | 100 | 100 | 4e-291 | [QXL90376.1](https://www.ncbi.nlm.nih.gov/protein/QXL90376.1?report=genbank&log$=prottop&blast_rank=1&RID=AUS60TCK013) | - |
| 72 | - | 35,854..  36,057 | 204 | 67 | 7.4 | 10 | ATG | Membrane protein (Salmonella phage PVPSE1) | 1001 | 100 | 2e-38 | [YP_004893922.1](https://www.ncbi.nlm.nih.gov/protein/YP_004893922.1?report=genbank&log$=prottop&blast_rank=1&RID=AUUWG6W6016) | - |
| 73 | - | 36,057..  36,371 | 315 | 104 | 11.9 | 7.9 | ATG | Transcriptional regulator (Salmonella phage PVPSE1) | 100 | 100 | 9e-69 | [YP_004893921.1](https://www.ncbi.nlm.nih.gov/protein/YP_004893921.1?report=genbank&log$=prottop&blast_rank=1&RID=AUVR9PN9016) | - |
| 74 | - | 36,440..  36,670 | 231 | 76 | 8.8 | 9.6 | GTG | Hypothetical protein (Salmonella phage NINP13076) | 98.68 | 100 | 4e-48 | [QXL90379.1](https://www.ncbi.nlm.nih.gov/protein/QXL90379.1?report=genbank&log$=prottop&blast_rank=1&RID=AUVZD8AR013) | - |
| 75 | - | 36,725..  37,417 | 693 | 230 | 26.1 | 5.8 | ATG | Hypothetical protein (Salmonella phage NINP13076) | 98.26 | 100 | 1e-167 | [QXL90380.1](https://www.ncbi.nlm.nih.gov/protein/QXL90380.1?report=genbank&log$=prottop&blast_rank=1&RID=AUW5W0DN016) | NTP-PPase super family ([cl16941](https://www.ncbi.nlm.nih.gov/Structure/cdd/cddsrv.cgi?ascbin=8&maxaln=10&seltype=2&uid=cl16941)) |
| 76 | - | 37,427..  37,798 | 372 | 123 | 13.8 | 9.1 | ATG | Hypothetical protein (Salmonella phage NINP13076) | 100 | 100 | 2e-38 | [YP_004893918.1](https://www.ncbi.nlm.nih.gov/protein/YP_004893918.1?report=genbank&log$=prottop&blast_rank=1&RID=AUX1U6RG016) | - |
| 77 | - | 37,798..  38,244 | 447 | 148 | 16.6 | 4.8 | ATG | Hypothetical protein (Salmonella phage PVPSE1) | 100 | 100 | 6e-104 | [YP_004893917.1](https://www.ncbi.nlm.nih.gov/protein/YP_004893917.1?report=genbank&log$=prottop&blast_rank=1&RID=AUXA4WJ8016) | - |
| 78 | - | 38,244..  39,545 | 1302 | 433 | 49.6 | 5.4 | ATG | ATP-dependent DNA ligase (Salmonella phage PVPSE1) | 99.08 | 100 | 0 | [YP_004893916.1](https://www.ncbi.nlm.nih.gov/protein/YP_004893916.1?report=genbank&log$=prottop&blast_rank=1&RID=AUXM8S9T016) | ATP-dependent, central (IPR012310), PRK09125 super family ([cl32352](https://www.ncbi.nlm.nih.gov/Structure/cdd/cddsrv.cgi?ascbin=8&maxaln=10&seltype=2&uid=cl32352)) |
| 79 | - | 39,545..  40,321 | 777 | 258 | 29.7 | 5.5 | GTG | NAD dependent protein deacetylase of SIR2 family (Salmonella phage NINP13076) | 99.611 | 100 | 0 | [QXL90519.1](https://www.ncbi.nlm.nih.gov/protein/QXL90519.1?report=genbank&log$=prottop&blast_rank=1&RID=AUYCXTN5013) | Sirtuin family (IPR003000) |
| 80 | - | 40,331..  40,735 | 405 | 134 | 15.1 | 5.7 | ATG | Hypothetical protein (Salmonella phage NINP13076) | 100 | 100 | 2E-95 | [QXL90518.1](https://www.ncbi.nlm.nih.gov/protein/QXL90518.1?report=genbank&log$=prottop&blast_rank=1&RID=AUYXXGTS013) | AAA_33 super family ([cl38431](https://www.ncbi.nlm.nih.gov/Structure/cdd/cddsrv.cgi?ascbin=8&maxaln=10&seltype=2&uid=cl38431)) |
| 81 | - | 40,745..  41,089 | 345 | 114 | 13.3 | 6.4 | ATG | Hypothetical protein (Salmonella phage NINP13076) | 98.25 | 100 | 5e-79 | [QXL90517.1](https://www.ncbi.nlm.nih.gov/protein/QXL90517.1?report=genbank&log$=prottop&blast_rank=1&RID=AUZP6UGP013) | Cyclic-phosphate processing, Receiver domain (IPR046909) |
| 82 | - | 41,086..  42,021 | 936 | 311 | 35.9 | 6.5 | ATG | Hypothetical protein (Salmonella phage NINP13076) | 98.71 | 100 | 0 | [QXL90516.1](https://www.ncbi.nlm.nih.gov/protein/QXL90516.1?report=genbank&log$=prottop&blast_rank=1&RID=AUZYK9N6016) | T4 RNA ligase 1 (IPR019031), RNA_lig_T4_1 super family ([cl09743](https://www.ncbi.nlm.nih.gov/Structure/cdd/cddsrv.cgi?ascbin=8&maxaln=10&seltype=2&uid=cl09743)) |
| 83 | - | 42,006..  42,602 | 597 | 198 | 23.1 | 8.4 | ATG | Hypothetical protein (Salmonella phage NINP13076) | 100 | 100 | 5e-146 | [QXL90465.1](https://www.ncbi.nlm.nih.gov/protein/QXL90465.1?report=genbank&log$=prottop&blast_rank=1&RID=AV0BVMK2016) | - |
| 84 | - | 42,586..  42,942 | 357 | 118 | 13.6 | 6.7 | ATG | Hypothetical protein (Salmonella phage NINP13076) | 100 | 100 | 2e-82 | [QXL90466.1](https://www.ncbi.nlm.nih.gov/protein/QXL90466.1?report=genbank&log$=prottop&blast_rank=1&RID=AV0GG5NW016) | - |
| 85 | - | 42,944..  43,795 | 852 | 283 | 31.4 | 6,1 | ATG | Putative type II 5-methy cytosine DNA methyltransferase (Salmonella phage NINP13076) | 100 | 100 | 0 | [QXL90467.1](https://www.ncbi.nlm.nih.gov/protein/QXL90467.1?report=genbank&log$=prottop&blast_rank=1&RID=AV0NFG9D013) | Dcm super family ([cl43082](https://www.ncbi.nlm.nih.gov/Structure/cdd/cddsrv.cgi?ascbin=8&maxaln=10&seltype=2&uid=cl43082)), [C-5 cytosine methyltransferase](https://www.ebi.ac.uk/interpro/entry/interpro/IPR001525)(IPR001525) |
| 86 | - | 43,803..  44,399 | 597 | 198 | 22.5 | 7.3 | ATG | Putative phosphoesterase (Salmonella phage NINP13076) | 98.99 | 100 | 7e-143 | [QXL90468.1](https://www.ncbi.nlm.nih.gov/protein/QXL90468.1?report=genbank&log$=prottop&blast_rank=1&RID=AV15PK7S013) | COG4186 super family ([cl42653](https://www.ncbi.nlm.nih.gov/Structure/cdd/cddsrv.cgi?ascbin=8&maxaln=10&seltype=2&uid=cl42653)), Calcineurin-like phosphoesterase domain, lpxH-type (IPR024654) |
| 87 | - | 44,396..  44,683 | 288 | 95 | 11.2 | 9.2 | ATG | Transposase like protein (Salmonella phage PVPSE1) | 98.95 | 100 | 3e-62 | [YP_004893908.1](https://www.ncbi.nlm.nih.gov/protein/YP_004893908.1?report=genbank&log$=prottop&blast_rank=1&RID=AV1P3RCS013) | - |
| 88 | - | 44,680..  44,964 | 285 | 94 | 10.8 | 6.7 | TTG | Hypothetical protein (Salmonella phage PVPSE1) | 80.85 | 100 | 9e-48 | [YP_004893907.1](https://www.ncbi.nlm.nih.gov/protein/YP_004893907.1?report=genbank&log$=prottop&blast_rank=1&RID=AV1UHZMZ016) | - |
| 89 | - | 45,076..  45,399 | 324 | 107 | 12.1 | 6.8 | ATG | Hypothetical protein (Salmonella phage PVPSE1) | 89.72 | 100 | 1e-63 | [YP_004893906.1](https://www.ncbi.nlm.nih.gov/protein/YP_004893906.1?report=genbank&log$=prottop&blast_rank=1&RID=AV222BGZ013) | - |
| 90 | + | 45,734..  46,090 | 357 | 118 | 13.8 | 9.3 | GTG | Hypothetical protein (Salmonella phage GEC-vB-MG) | 99.15 | 100 | 1e-80 | [QPI14651.1](https://www.ncbi.nlm.nih.gov/protein/QPI14651.1?report=genbank&log$=prottop&blast_rank=1&RID=AV2M1KGB013) | HTH super family ([cl21459](https://www.ncbi.nlm.nih.gov/Structure/cdd/cddsrv.cgi?ascbin=8&maxaln=10&seltype=2&uid=cl21459)) |
| 91 | + | 46,099..  46,659 | 561 | 186 | 20.9 | 7.6 | ATG | Hypothetical protein (Salmonella phage PVPSE1) | 98.39 | 100 | 5e-132 | [YP_004893904.1](https://www.ncbi.nlm.nih.gov/protein/YP_004893904.1?report=genbank&log$=prottop&blast_rank=1&RID=AW4PX00D016) | Winged helix-like DNA-binding domain superfamily (IPR036388) |
| 92 | + | 46,661..  47,464 | 804 | 267 | 29.3 | 6 | ATG | Ribose-phosphate pyrophosphokinase family protein (Salmonella phage NINP13076) | 98.50 | 100 | 0 | [QXL90350.1](https://www.ncbi.nlm.nih.gov/protein/QXL90350.1?report=genbank&log$=prottop&blast_rank=1&RID=AW56MWNR013) | PrsA super family ([cl33889](https://www.ncbi.nlm.nih.gov/Structure/cdd/cddsrv.cgi?ascbin=8&maxaln=10&seltype=2&uid=cl33889)), [Ribose-phosphate pyrophosphokinase](https://www.ebi.ac.uk/interpro/entry/interpro/IPR005946)(IPR005946) |
| 93 | + | 47,532..  49,226 | 1695 | 564 | 64.3 | 5.1 | ATG | Nicotinamide phosphoribosyltransferase (Salmonella phage PVPSE1) | 95.04 | 100 | 0 | [YP_004893902.1](https://www.ncbi.nlm.nih.gov/protein/YP_004893902.1?report=genbank&log$=prottop&blast_rank=1&RID=AW5P2VV3016) | PRK09198 super family ([cl30369](https://www.ncbi.nlm.nih.gov/Structure/cdd/cddsrv.cgi?ascbin=8&maxaln=10&seltype=2&uid=cl30369)), [Nicotinamide phosphoribosyl transferase](https://www.ebi.ac.uk/interpro/entry/interpro/IPR016471)(IPR016471) |
| 94 | + | 49,362..  49,550 | 189 | 62 | 6.9 | 5.1 | ATG | Hypothetical protein (Salmonella phage NINP13076) | 96.77 | 100 | 3e-37 | [QXL90348.1](https://www.ncbi.nlm.nih.gov/protein/QXL90348.1?report=genbank&log$=prottop&blast_rank=1&RID=AW5X21PJ013) | - |
| 95 | + | 49,560..  49,766 | 207 | 68 | 7.7 | 4.5 | GTG | Hypothetical protein (Salmonella phage NINP13076) | 98.53 | 100 | 2e-41 | [QXL90347.1](https://www.ncbi.nlm.nih.gov/protein/QXL90347.1?report=genbank&log$=prottop&blast_rank=1&RID=AW63CDC101N) | - |
| 96 | + | 49,848..  50,093 | 246 | 81 | 9,3 | 4.7 | ATG | Hypothetical protein (Salmonella phage NINP13076) | 100 | 100 | 2e-51 | [QXL90346.1](https://www.ncbi.nlm.nih.gov/protein/QXL90346.1?report=genbank&log$=prottop&blast_rank=1&RID=AW6846VH01N) | - |
| 97 | + | 50,157..  50,276 | 120 | 39 | 4.4 | 3.9 | ATG | Hypothetical protein (Salmonella phage NINP13076) | 100 | 100 | 2e-17 | [QXL90345.1](https://www.ncbi.nlm.nih.gov/protein/QXL90345.1?report=genbank&log$=prottop&blast_rank=1&RID=AW6DNXGP016) | - |
| 98 | + | 50,279..  50,449 | 171 | 56 | 6.5 | 8.3 | ATG | Hypothetical protein (Salmonella phage NINP13076) | 100 | 100 | 1e-33 | [QXL90344.1](https://www.ncbi.nlm.nih.gov/protein/QXL90344.1?report=genbank&log$=prottop&blast_rank=1&RID=AW6P5UKU016) | - |
| 99 | + | 50,564..  50,719 | 156 | 51 | 6.1 | 9.6 | ATG | Hypothetical protein (Salmonella phage NINP13076) | 100 | 100 | 8e-30 | [QXL90343.1](https://www.ncbi.nlm.nih.gov/protein/QXL90343.1?report=genbank&log$=prottop&blast_rank=1&RID=AW6ZT9HU013) | - |
| 100 | + | 50,804..  51,133 | 330 | 109 | 12.0 | 10.4 | ATG | Hypothetical protein (Salmonella phage 41) | 93.58 | 100 | 3e-66 | [AKJ73432.1](https://www.ncbi.nlm.nih.gov/protein/AKJ73432.1?report=genbank&log$=prottop&blast_rank=1&RID=AW74N15J016) | - |
| 101 | + | 51575..51763 | 189 | 62 | 6.3 | 3.9 | ATG | Hypothetical protein (Escherichia phage 4MG) | 95.16 | 100 | 8e-32 | [YP_008857299.1](https://www.ncbi.nlm.nih.gov/protein/YP_008857299.1?report=genbank&log$=prottop&blast_rank=1&RID=AXGWF41M013) | - |
| 102 | + | 51760..51897 | 138 | 45 | 5.2 | 4.8 | ATG | Membrane protein (Salmonella phage PVPSE1) | 97.78 | 100 | 8e-24 | [YP_004893891.1](https://www.ncbi.nlm.nih.gov/protein/YP_004893891.1?report=genbank&log$=prottop&blast_rank=1&RID=AXKKE0Y1013) | - |
| 103 | + | 51939..52550 | 612 | 203 | 21.8 | 4.9 | ATG | Hypothetical protein (Salmonella phage NINP13076) | 96.55 | 100 | 2e-132 | [QXL90339.1](https://www.ncbi.nlm.nih.gov/protein/QXL90339.1?report=genbank&log$=prottop&blast_rank=1&RID=AXKSYV01013) | PRK12472 super family ([cl39155](https://www.ncbi.nlm.nih.gov/Structure/cdd/cddsrv.cgi?ascbin=8&maxaln=10&seltype=2&uid=cl39155)) |
| 104 | + | 52624..53283 | 660 | 219 | 24.1 | 5.5 | ATG | Hypothetical protein (Salmonella phage NINP13076) | 98.17 | 100 | 2e-154 | [QXL90338.1](https://www.ncbi.nlm.nih.gov/protein/QXL90338.1?report=genbank&log$=prottop&blast_rank=1&RID=AXM0EMTE013) | chemoreceptor_sensor super family ([cl00144](https://www.ncbi.nlm.nih.gov/Structure/cdd/cddsrv.cgi?ascbin=8&maxaln=10&seltype=2&uid=cl00144)) |
| 105 | + | 55063..55428 | 366 | 121 | 13.8 | 5.5 | ATG | Hypothetical protein (Salmonella phage NINP13076) | 100 | 100 | 2e-82 | [QXL90333.1](https://www.ncbi.nlm.nih.gov/protein/QXL90333.1?report=genbank&log$=prottop&blast_rank=1&RID=AXM702KW016) | - |
| 106 | + | 55458..55622 | 165 | 54 | 5.8 | 10.8 | ATG | Hypothetical protein (Salmonella phage PVPSE1) | 100 | 100 | 2e-26 | [YP_004893887.1](https://www.ncbi.nlm.nih.gov/protein/YP_004893887.1?report=genbank&log$=prottop&blast_rank=1&RID=AXMDRWKY016) | [Protein of unknown function DUF1328](https://www.ebi.ac.uk/interpro/entry/interpro/IPR009760)(IPR009760), DUF1328 super family ([cl26923](https://www.ncbi.nlm.nih.gov/Structure/cdd/cddsrv.cgi?ascbin=8&maxaln=10&seltype=2&uid=cl26923)) |
| 107 | + | 56347..56511 | 165 | 54 | 6.2 | 9.4 | ATG | Hypothetical protein (Salmonella phage SSE121) | 100 | 100 | 5e-30 | [YP_009148801.1](https://www.ncbi.nlm.nih.gov/protein/YP_009148801.1?report=genbank&log$=prottop&blast_rank=1&RID=AXMSZDYY016) | - |
| 108 | + | 56508..56636 | 129 | 42 | 4.7 | 4.7 | ATG | Hypothetical protein (Salmonella phage SSE121) | 100 | 100 | 2e-19 | [YP_009148802.1](https://www.ncbi.nlm.nih.gov/protein/YP_009148802.1?report=genbank&log$=prottop&blast_rank=1&RID=AXMXRP27013) | - |
| 109 | + | 57,074..57,475 | 402 | 133 | 15.4 | 9.4 | ATG | Hypothetical protein (Salmonella phage NINP13076) | 100 | 100 | 1e-94 | [QXL90327.1](https://www.ncbi.nlm.nih.gov/protein/QXL90327.1?report=genbank&log$=prottop&blast_rank=1&RID=AXN1ZSU3013) | - |
| 110 | + | 57,829..57,981 | 153 | 50 | 5.9 | 4.2 | ATG | Hypothetical protein (Salmonella phage 19) | 100 | 100 | 5e-29 | [AKJ74684.1](https://www.ncbi.nlm.nih.gov/protein/AKJ74684.1?report=genbank&log$=prottop&blast_rank=1&RID=AXN9W5VU013) | - |
| 111 | + | 58,086..58,235 | 150 | 49 | 5.5 | 6.5 | ATG | Hypothetical protein (Salmonella phage vB_SenS-3) | 79.41 | 69 | 6e-11 | [QIN93415.1](https://www.ncbi.nlm.nih.gov/protein/QIN93415.1?report=genbank&log$=prottop&blast_rank=1&RID=AYM79ZV4013) | - |
| 112 | - | 58620..58736 | 117 | 38 | 4.3 | 7.9 | ATG | Hypothetical protein (Salmonella phage 19) | 97.37 | 100 | 2e-18 | [AKJ74685.1](https://www.ncbi.nlm.nih.gov/protein/AKJ74685.1?report=genbank&log$=prottop&blast_rank=1&RID=AYMJRW9101N) | - |
| 113 | - | 58,736..58,954 | 219 | 72 | 8.5 | 9.9 | ATG | Hypothetical protein (Salmonella phage NINP13076) | 90 | 96.92 | 2e-39 | [QXL90323.1](https://www.ncbi.nlm.nih.gov/protein/QXL90323.1?report=genbank&log$=prottop&blast_rank=1&RID=AXNFY2W8016) | - |
| 114 | + | 58,953..60,380 | 1428 | 475 | 54.1 | 5.6 | ATG | Hypothetical protein (Salmonella phage NINP13076) | 96.84 | 100 | 0 | [QXL90322.1](https://www.ncbi.nlm.nih.gov/protein/QXL90322.1?report=genbank&log$=prottop&blast_rank=1&RID=AXNR08UY016) | - |
| 115 | + | 60,377..60,781 | 405 | 134 | 15.2 | 7.7 | ATG | Hypothetical protein (Salmonella phage NINP13076) | 97.01 | 100 | 4e-87 | [QXL90321.1](https://www.ncbi.nlm.nih.gov/protein/QXL90321.1?report=genbank&log$=prottop&blast_rank=1&RID=AXNWZU4701N) | - |
| 116 | + | 60,778..61,065 | 288 | 95 | 10.4 | 8.8 | ATG | Hypothetical protein (Salmonella phage NINP13076) | 90.53 | 100 | 2e-55 | [QXL90320.1](https://www.ncbi.nlm.nih.gov/protein/QXL90320.1?report=genbank&log$=prottop&blast_rank=1&RID=AYJKFBCZ01N) | - |
| 117 | + | 61,164..62,663 | 1500 | 499 | 56.9 | 5.7 | TTG | Terminase large subnit (Salmonella phage 19) | 99.60 | 100 | 0 | [AKJ74499.1](https://www.ncbi.nlm.nih.gov/protein/AKJ74499.1?report=genbank&log$=prottop&blast_rank=1&RID=AYJXZYVK013) | Terminase_6 super family  ([cl40838](https://www.ncbi.nlm.nih.gov/Structure/cdd/cddsrv.cgi?ascbin=8&maxaln=10&seltype=2&uid=cl40838)), Terminase, large subunit gp17-like, C-terminal (IPR035421) |
| 118 | + | 62,679..64,229 | 1551 | 516 | 56.6 | 5.2 | GTG | Hypothetical protein (Salmonella phage PVPSE1) | 93.8 | 100 | 0 | [YP_004893881.1](https://www.ncbi.nlm.nih.gov/protein/YP_004893881.1?report=genbank&log$=prottop&blast_rank=1&RID=AYKN9CSD013) | - |
| 119 | + | 64,299..64,925 | 627 | 208 | 22.7 | 4.5 | ATG | Hypothetical protein (Salmonella phage NINP13076) | 100 | 100 | 1e-146 | [QXL90315.1](https://www.ncbi.nlm.nih.gov/protein/QXL90315.1?report=genbank&log$=prottop&blast_rank=1&RID=AYPCFA2X01N) | Phage-like element PBSX protein, XkdF (IPR027924), Peptidase_S78_2 super family ([cl24270](https://www.ncbi.nlm.nih.gov/Structure/cdd/cddsrv.cgi?ascbin=8&maxaln=10&seltype=2&uid=cl24270)) |
| 120 | + | 64,922..65,923 | 1002 | 333 | 36.6 | 4.5 | ATG | Hypothetical protein (Salmonella phage NINP13076) | 100 | 100 | 0 | [QXL90314.1](https://www.ncbi.nlm.nih.gov/protein/QXL90314.1?report=genbank&log$=prottop&blast_rank=1&RID=AYPSK7EB013) | - |
| 121 | + | 65,944..66,363 | 420 | 139 | 14.9 | 4.3 | ATG | Hypothetical protein (Salmonella phage NINP13076) | 100 | 100 | 7e-94 | [QXL90313.1](https://www.ncbi.nlm.nih.gov/protein/QXL90313.1?report=genbank&log$=prottop&blast_rank=1&RID=AYPZBEFE013) | - |
| 122 | + | 66,385..67,398 | 1014 | 337 | 38.5 | 5.2 | ATG | Major capsid protein (Salmonella phage NINP13076) | 100 | 100 | 0 | [QXL90312.1](https://www.ncbi.nlm.nih.gov/protein/QXL90312.1?report=genbank&log$=prottop&blast_rank=1&RID=AYRMA29H016) | Phage_cap_E super family ([cl20258](https://www.ncbi.nlm.nih.gov/Structure/cdd/cddsrv.cgi?ascbin=8&maxaln=10&seltype=2&uid=cl20258)),  [Major capsid protein GpE](https://www.ebi.ac.uk/interpro/entry/interpro/IPR005564)(IPR005564) |
| 123 | + | 67,530..68,063 | 534 | 177 | 19.4 | 6.3 | ATG | Hypothetical protein (Salmonella phage NINP13076) | 100 | 100 | 4e-124 | [QXL90311.1](https://www.ncbi.nlm.nih.gov/protein/QXL90311.1?report=genbank&log$=prottop&blast_rank=1&RID=AYRRVM1W013) | - |
| 124 | + | 68,073..70,829 | 2757 | 918 | 93.7 | 4.1 | ATG | Tail fiber protein (Salmonella phage PVPSE1) | 99.46 | 100 | 0 | [YP_004893875.1](https://www.ncbi.nlm.nih.gov/protein/YP_004893875.1?report=genbank&log$=prottop&blast_rank=2&RID=AYRWJ6AX013) | gly_rich_SclB super family ([cl45768](https://www.ncbi.nlm.nih.gov/Structure/cdd/cddsrv.cgi?ascbin=8&maxaln=10&seltype=2&uid=cl45768)) |
| 125 | + | 70,865..71,509 | 645 | 214 | 24.4 | 4.3 | ATG | Hypothetical protein (Salmonella phage PVPSE1) | 99.53 | 100 | 1e-55 | [YP_004893874.1](https://www.ncbi.nlm.nih.gov/protein/YP_004893874.1?report=genbank&log$=prottop&blast_rank=1&RID=AYS6ZG7V013) | - |
| 126 | + | 71,523..71,777 | 255 | 84 | 8.1 | 4.9 | ATG | Head fiber protein (Salmonella phage PVPSE1) | 100 | 100 | 5e-48 | [YP_004893873.1](https://www.ncbi.nlm.nih.gov/protein/YP_004893873.1?report=genbank&log$=prottop&blast_rank=1&RID=AYSAECYA016) | Phage_head_fibr super family ([cl12685](https://www.ncbi.nlm.nih.gov/Structure/cdd/cddsrv.cgi?ascbin=8&maxaln=10&seltype=2&uid=cl12685)), [Bacteriophage B103, Gp8, head fibre](https://www.ebi.ac.uk/interpro/entry/interpro/IPR022741)(IPR022741) |
| 127 | - | 71,799.. 72,203 | 405 | 134 | 14.7 | 9 | ATG | Membrane protein (Salmonella phage PVPSE1) | 100 | 100 | 2e-94 | [YP_004893872.1](https://www.ncbi.nlm.nih.gov/protein/YP_004893872.1?report=genbank&log$=prottop&blast_rank=1&RID=AYSZ5USY016) | - |
| 128 | - | 72,337..72,855 | 519 | 172 | 19.3 | 5.6 | ATG | Hypothetical protein (Salmonella phage PVPSE1) | 100 | 100 | 3e-122 | [YP_004893871.1](https://www.ncbi.nlm.nih.gov/protein/YP_004893871.1?report=genbank&log$=prottop&blast_rank=1&RID=AYT2XPZG013) | - |
| 129 | + | 72,921..73,397 | 477 | 158 | 17.6 | 9.6 | ATG | Tail completion or Neck1 protein (Salmonella phage PVPSE1) | 99.37 | 100 | 4e-113 | [YP_004893870.1](https://www.ncbi.nlm.nih.gov/protein/YP_004893870.1?report=genbank&log$=prottop&blast_rank=1&RID=AYTB4UKV016) | - |
| 130 | + | 73,397..73,831 | 435 | 144 | 16.1 | 5.8 | ATG | Minor head protein (Salmonella phage PVPSE1) | 100 | 100 | 2e-102 | [YP_004893869.1](https://www.ncbi.nlm.nih.gov/protein/YP_004893869.1?report=genbank&log$=prottop&blast_rank=1&RID=AYTDV4GT016) | - |
| 131 | + | 73,831..74,361 | 531 | 176 | 19.6 | 4.5 | ATG | Hypothetical protein (Salmonella phage PVPSE1) | 100 | 100 | 4e-128 | [YP_004893868.1](https://www.ncbi.nlm.nih.gov/protein/YP_004893868.1?report=genbank&log$=prottop&blast_rank=1&RID=AYTKHF19013) |  |
| 132 | + | 74,491..75,903 | 1413 | 470 | 50.3 | 4.8 | ATG | Tail sheath (Salmonella phage PVPSE1) | 100 | 100 | 0 | [YP_004893867.1](https://www.ncbi.nlm.nih.gov/protein/YP_004893867.1?report=genbank&log$=prottop&blast_rank=1&RID=AYTSHBEV013) | DUF3383 super family ([cl20432](https://www.ncbi.nlm.nih.gov/Structure/cdd/cddsrv.cgi?ascbin=8&maxaln=10&seltype=2&uid=cl20432)), [Protein of unknown function DUF3383](https://www.ebi.ac.uk/interpro/entry/interpro/IPR021808)(IPR021808) |
| 133 | + | 75,907..76,380 | 474 | 157 | 16.9 | 4.6 | ATG | Virion structural protein (Salmonella phage PVPSE1) | 100 | 100 | 7e-110 | [YP_004893866.1](https://www.ncbi.nlm.nih.gov/protein/YP_004893866.1?report=genbank&log$=prottop&blast_rank=1&RID=AYTYK7JE01N) | [Structural protein ORF10, bacteriophage KPP10](https://www.ebi.ac.uk/interpro/entry/interpro/IPR021695)(IPR021695), DUF3277 super family ([cl13181](https://www.ncbi.nlm.nih.gov/Structure/cdd/cddsrv.cgi?ascbin=8&maxaln=10&seltype=2&uid=cl13181)) |
| 134 | + | 76,455..76,928 | 474 | 157 | 17.2 | 4.6 | ATG | Tail assembly chaperone (Salmonella phage PVPSE1) | 100 | 100 | 3e-111 | [YP_004893865.1](https://www.ncbi.nlm.nih.gov/protein/YP_004893865.1?report=genbank&log$=prottop&blast_rank=1&RID=AYU5930H01N) | - |
| 135 | + | 76,964..77,206 | 243 | 80 | 9.4 | 6.5 | ATG | Hypothetical protein (Salmonella phage PVPSE1) | 100 | 100 | 3e-51 | [YP_004893864.1](https://www.ncbi.nlm.nih.gov/protein/YP_004893864.1?report=genbank&log$=prottop&blast_rank=1&RID=AYY0GTDA013) | - |
| 136 | + | 77,260..79,647 | 2388 | 795 | 87.46 | 9.7 | ATG | Tail length tape measure protein (Salmonella phage SSE121) | 100 | 100 | 0 | [YP_009148827.1](https://www.ncbi.nlm.nih.gov/protein/YP_009148827.1?report=genbank&log$=prottop&blast_rank=1&RID=AYY6P82U016) | TMP_3 super family ([cl45527](https://www.ncbi.nlm.nih.gov/Structure/cdd/cddsrv.cgi?ascbin=8&maxaln=10&seltype=2&uid=cl45527)), Tape measure protein N-terminal (IPR013491) |
| 137 | + | 79,729..80,601 | 873 | 290 | 32 | 5.4 | ATG | Hypothetical protein (Salmonella phage SSE121) | 100 | 100 | 0 | [YP_009148828.1](https://www.ncbi.nlm.nih.gov/protein/YP_009148828.1?report=genbank&log$=prottop&blast_rank=1&RID=AYYE1UZ801N) | - |
| 138 | + | 80,601..80,954 | 354 | 117 | 13.6 | 4.2 | ATG | Virion structural protein (Salmonella phage PVPSE1) | 100 | 100 |  |  |  |
| 139 | + | 80,958..81,947 | 990 | 329 | 36.3 | 9 | ATG | Baseplate hub (Salmonella phage PVPSE1) | 100 | 100 | 0 | [YP_004893860.1](https://www.ncbi.nlm.nih.gov/protein/YP_004893860.1?report=genbank&log$=prottop&blast_rank=1&RID=AYYT4JDD016) | - |
| 140 | + | 81,947..82,648 | 702 | 233 | 25.1 | 4.6 | ATG | Baseplate spike (Salmonella phage PVPSE1) | 100 | 100 | 2e-169 | [YP_004893859.1](https://www.ncbi.nlm.nih.gov/protein/YP_004893859.1?report=genbank&log$=prottop&blast_rank=1&RID=AYYZGFZH016) | Gp138_N super family ([cl39697](https://www.ncbi.nlm.nih.gov/Structure/cdd/cddsrv.cgi?ascbin=8&maxaln=10&seltype=2&uid=cl39697)), [Phage protein Gp138 N-terminal domain](https://www.ebi.ac.uk/interpro/entry/pfam/PF18352/) (IPR041599) |
| 141 | + | 82,658..83,275 | 618 | 205 | 23.8 | 4.6 | ATG | Hypothetical protein (Salmonella phage PVPSE1) | 100 | 100 | 5e-149 | [YP_004893858.1](https://www.ncbi.nlm.nih.gov/protein/YP_004893858.1?report=genbank&log$=prottop&blast_rank=1&RID=AYZ3N9ZE013) | - |
| 142 | + | 83,286..83,960 | 675 | 224 | 23.7 | 5 | ATG | Tail fiber protein (Salmonella phage PVPSE1) | 100 | 100 | 2e-160 | [YP_004893857.1](https://www.ncbi.nlm.nih.gov/protein/YP_004893857.1?report=genbank&log$=prottop&blast_rank=1&RID=AYZ926AD01N) | Tail_spike_N super family  ([cl39985](https://www.ncbi.nlm.nih.gov/Structure/cdd/cddsrv.cgi?ascbin=8&maxaln=10&seltype=2&uid=cl39985)), Tail spike TSP1/Gp66, N-terminal domain (IPR040775) |
| 143 | + | 83,960..86,749 | 2790 | 929 | 105.8 | 4.6 | ATG | Tail protein (Salmonella phage SSE121) | 100 | 100 | 0 | [YP_009148834.1](https://www.ncbi.nlm.nih.gov/protein/YP_009148834.1?report=genbank&log$=prottop&blast_rank=1&RID=AYZNVTDY016) | Six-hairpin glycosidase superfamily (IPR008928) |
| 144 | + | 86,759..88,897 | 2139 | 712 | 77.3 | 5.1 | ATG | Colanic acid degradation (Salmonella phage SSE121) | 99.86 | 100 | 0 | [YP_009148835.1](https://www.ncbi.nlm.nih.gov/protein/YP_009148835.1?report=genbank&log$=prottop&blast_rank=1&RID=AYZY2W9G016) | Tail spike TSP1/Gp66, N-terminal domain (IPR040775), wcaM super family ([cl22684](https://www.ncbi.nlm.nih.gov/Structure/cdd/cddsrv.cgi?ascbin=8&maxaln=10&seltype=2&uid=cl22684)), Tail_spike_N super family ([cl39985](https://www.ncbi.nlm.nih.gov/Structure/cdd/cddsrv.cgi?ascbin=8&maxaln=10&seltype=2&uid=cl39985)) |
| 145 | + | 89,006..90,499 | 1494 | 497 | 54.0 | 4.5 | GTG | Hypothetical protein (Salmonella phage NINP13076) | 99.2 | 100 | 0 | [QXL90288.1](https://www.ncbi.nlm.nih.gov/protein/QXL90288.1?report=genbank&log$=prottop&blast_rank=1&RID=AZ0P8B7B01N) | Baseplate protein J-like  (IPR006949), Baseplate_J super family ([cl01294](https://www.ncbi.nlm.nih.gov/Structure/cdd/cddsrv.cgi?ascbin=8&maxaln=10&seltype=2&uid=cl01294)) |
| 146 | + | 90,510..91,142 | 633 | 210 | 23.4 | 4.9 | ATG | Base plate protein (Salmonella phage PVPSE1) | 99.52 | 100 | 1e-151 | [YP_004893853.1](https://www.ncbi.nlm.nih.gov/protein/YP_004893853.1?report=genbank&log$=prottop&blast_rank=1&RID=AZ12MSAG013) | - |
| 147 | + | 91,154..92,275 | 1122 | 373 | 39.8 | 5.7 | ATG | Tail fiber protein (Salmonella phage SSE121) | 99.73 | 100 | 0 | [YP_009148838.1](https://www.ncbi.nlm.nih.gov/protein/YP_009148838.1?report=genbank&log$=prottop&blast_rank=1&RID=AZ1F9R2V013) | - |
| 148 | + | 92,285..92,839 | 555 | 184 | 20.5 | 4.6 | ATG | Tail assembly chaperone (Salmonella phage PVPSE1) | 100 | 100 | 1e-129 | [YP_004893851.1](https://www.ncbi.nlm.nih.gov/protein/YP_004893851.1?report=genbank&log$=prottop&blast_rank=1&RID=AZ1M5Z4S013) | Caudo_TAP super family ([cl17077](https://www.ncbi.nlm.nih.gov/Structure/cdd/cddsrv.cgi?ascbin=8&maxaln=10&seltype=2&uid=cl17077)), Bacteriophage T4, Gp38, tail fibre assembly (IPR003458) |
| 149 | + | 92,839..93,153 | 315 | 104 | 12.2 | 6.3 | ATG | Hypothetical protein (Salmonella phage PVPSE1) | 100 | 100 | 3e-64 | [YP_004893850.1](https://www.ncbi.nlm.nih.gov/protein/YP_004893850.1?report=genbank&log$=prottop&blast_rank=1&RID=AZ1XJB4W013) | - |
| 150 | + | 93,150..93,623 | 474 | 157 | 18 | 8.61 | ATG | Hypothetical membrane protein (Salmonella phage PVPSE1) | 100 | 100 | 2e-106 | [YP_004893849.1](https://www.ncbi.nlm.nih.gov/protein/YP_004893849.1?report=genbank&log$=prottop&blast_rank=1&RID=AZ2X1MY2016) | - |
| 151 | + | 93,639..93,908 | 270 | 89 | 10.2 | 9.4 | ATG | Membrane protein (Salmonella phage PVPSE1) | 100 | 100 | 1e-58 | [YP_004893848.1](https://www.ncbi.nlm.nih.gov/protein/YP_004893848.1?report=genbank&log$=prottop&blast_rank=1&RID=AZ30EEV0016) | - |
| 152 | + | 93,919..95,727 | 1809 | 602 | 62.2 | 3.9 | GTG | Tail fiber protein (Salmonella phage PVPSE1) | 99.83 | 100 | 0 | [YP_004893847.1](https://www.ncbi.nlm.nih.gov/protein/YP_004893847.1?report=genbank&log$=prottop&blast_rank=1&RID=AZ3CD5J5016) | 34 super family ([cl33689](https://www.ncbi.nlm.nih.gov/Structure/cdd/cddsrv.cgi?ascbin=8&maxaln=10&seltype=2&uid=cl33689)) |
| 153 | + | 95,773..97,587 | 1815 | 604 | 64.1 | 4.3 | ATG | Tail protein (Salmonella phage PVPSE1) | 99.5 | 100 | 0 | [YP_004893846.1](https://www.ncbi.nlm.nih.gov/protein/YP_004893846.1?report=genbank&log$=prottop&blast_rank=1&RID=AZ3H8P6101N) | Regulator of chromosome condensation 1/beta-lactamase-inhibitor protein II (IPROO9091)  Big_2 super family (  [cl02708](https://www.ncbi.nlm.nih.gov/Structure/cdd/cddsrv.cgi?ascbin=8&maxaln=10&seltype=2&uid=cl02708)) |
| 154 | - | 97,623..98,852 | 1230 | 409 | 43.4 | 5.5 | ATG | Hypothetical protein (Salmonella phage PVPSE1) | 99.76 | 100 | 0 | [YP_004893845.1](https://www.ncbi.nlm.nih.gov/protein/YP_004893845.1?report=genbank&log$=prottop&blast_rank=1&RID=AZ43JGB201N) | RPA_2b-aaRSs_OBF_like super family ([cl09930](https://www.ncbi.nlm.nih.gov/Structure/cdd/cddsrv.cgi?ascbin=8&maxaln=10&seltype=2&uid=cl09930)) |
| 155 | - | 98,864.. 99,217 | 354 | 117 | 13.0 | 4.4 | ATG | Hypothetical protein (Salmonella phage PVPSE1) | 100 | 100 | 9e-78 | [YP_004893844.1](https://www.ncbi.nlm.nih.gov/protein/YP_004893844.1?report=genbank&log$=prottop&blast_rank=1&RID=AZ476T2R013) | - |
| 156 | - | 99,277..99,489 | 213 | 70 | 7.8 | 10.4 | ATG | Hypothetical membrane protein (Salmonella phage PVPSE1) | 100 | 100 | 9e-38 | [YP_004893843.1](https://www.ncbi.nlm.nih.gov/protein/YP_004893843.1?report=genbank&log$=prottop&blast_rank=1&RID=AZ4D8N6E013) | small_Trp_rich super family ([cl22805](https://www.ncbi.nlm.nih.gov/Structure/cdd/cddsrv.cgi?ascbin=8&maxaln=10&seltype=2&uid=cl22805)) |
| 157 | - | 99,489.. 99,713 | 225 | 74 | 8.0 | 6.0 | TTG | Hypothetical protein (Salmonella phage PVPSE1) | 98.65 | 100 | 1e-46 | [YP_004893842.1](https://www.ncbi.nlm.nih.gov/protein/YP_004893842.1?report=genbank&log$=prottop&blast_rank=1&RID=AZ4S09MZ016) | - |
| 158 | - | 99,710..1,00,228 | 519 | 172 | 18.9 | 9.2 | ATG | Hypothetical protein (Salmonella phage PVPSE1) | 99.42 | 100 | 9e-121 | [YP_004893841.1](https://www.ncbi.nlm.nih.gov/protein/YP_004893841.1?report=genbank&log$=prottop&blast_rank=1&RID=AZ4UYED2013) | - |
| 159 | - | 1,00,221.. 1,00,499 | 279 | 92 | 10.8 | 4.6 | ATG | Hypothetical protein (Salmonella phage PVPSE1) | 100 | 100 | 8e-62 | [YP_004893840.1](https://www.ncbi.nlm.nih.gov/protein/YP_004893840.1?report=genbank&log$=prottop&blast_rank=1&RID=AZ60D2DZ016) | PHA02053 super family ([cl10356](https://www.ncbi.nlm.nih.gov/Structure/cdd/cddsrv.cgi?ascbin=8&maxaln=10&seltype=2&uid=cl10356)) |
| 160 | - | 1,00,490..1,00,861 | 372 | 123 | 14.4 | 7.7 | ATG | Hypothetical membrane protein (Salmonella phage PVPSE1) | 100 | 100 | 2e-86 | [YP_004893839.1](https://www.ncbi.nlm.nih.gov/protein/YP_004893839.1?report=genbank&log$=prottop&blast_rank=1&RID=AZ6VXE70016) | - |
| 161 | - | 1,00,861.. 1,01,571 | 711 | 236 | 26.6 | 8.7 | ATG | PnuC-like nicotinamide mononucleotide transport (Salmonella phage PVPSE1) | 98.73 | 100 | 1e-163 | [YP_004893838.1](https://www.ncbi.nlm.nih.gov/protein/YP_004893838.1?report=genbank&log$=prottop&blast_rank=1&RID=AZ70JUD3013) | NMN_transporter super family ([cl01256](https://www.ncbi.nlm.nih.gov/Structure/cdd/cddsrv.cgi?ascbin=8&maxaln=10&seltype=2&uid=cl01256)), Nicotinamide mononucleotide transporter PnuC (IPR006419) |
| 162 | - | 1,01,586.. 1,02,236 | 651 | 216 | 25.2 | 8.6 | ATG | HNH endonuclease (Salmonella phage PVPSE1) | 100 | 100 | 1e-160 | [YP_004893837.1](https://www.ncbi.nlm.nih.gov/protein/YP_004893837.1?report=genbank&log$=prottop&blast_rank=1&RID=AZ7WMKRN013) | - |
| 163 | - | 1,02,238.. 1,02,429 | 192 | 63 | 6.8 | 5.8 | ATG | Hypothetical protein (Salmonella phage PVPSE1) | 100 | 100 | 6e-38 | [YP_004893836.1](https://www.ncbi.nlm.nih.gov/protein/YP_004893836.1?report=genbank&log$=prottop&blast_rank=1&RID=AZ81G1TD013) | - |
| 164 | - | 1,02,426.. 1,02,752 | 327 | 108 | 12.3 | 9.11 | ATG | Hypothetical protein (Salmonella phage PVPSE1) | 100 | 81 | 4e-58 | [YP_004893835.1](https://www.ncbi.nlm.nih.gov/protein/YP_004893835.1?report=genbank&log$=prottop&blast_rank=1&RID=AZ85FEMX016) | DUF4326 super family ([cl16717](https://www.ncbi.nlm.nih.gov/Structure/cdd/cddsrv.cgi?ascbin=8&maxaln=10&seltype=2&uid=cl16717)), [Protein of unknown function DUF4326)](https://www.ebi.ac.uk/interpro/entry/interpro/IPR025475)(IPR025475) |
| 165 | - | 1,02,745.. 1,02,954 | 210 | 69 | 7.8 | 4.8 | ATG | Hypothetical protein (Salmonella phage PVPSE1) | 100 | 100 | 1e-42 | [YP_004893834.1](https://www.ncbi.nlm.nih.gov/protein/YP_004893834.1?report=genbank&log$=prottop&blast_rank=1&RID=AZ8PRKD8016) | - |
| 166 | - | 1,02,941.. 1,03,306 | 366 | 121 | 13.9 | 4.7 | ATG | Hypothetical protein (Salmonella phage PVPSE1) | 95.87 | 100 | 2e-82 | [YP_004893833.1](https://www.ncbi.nlm.nih.gov/protein/YP_004893833.1?report=genbank&log$=prottop&blast_rank=1&RID=AZ8ZH780016) | - |
| 167 | - | 1,03,308..1,04,378 | 1071 | 356 | 40.9 | 5.6 | ATG | Nicotinamide-nucleotide adenylyltransferase (Escherichia phage 4MG) | 81.51 | 99 | 0 | [YP_008857235.1](https://www.ncbi.nlm.nih.gov/protein/YP_008857235.1?report=genbank&log$=prottop&blast_rank=1&RID=AZ961JE9016) | nadR_NMN_Atrans super family ([cl36926](https://www.ncbi.nlm.nih.gov/Structure/cdd/cddsrv.cgi?ascbin=8&maxaln=10&seltype=2&uid=cl36926)), NadR/Ttd14, AAA domain (IPR038727) |
| 168 | - | 1,04,375.. 1,04,635 | 261 | 86 | 9.3 | 7.9 | ATG | Hypothetical protein (Salmonella phage NINP13076) | 100 | 100 | 8e-53 | [QXL90447.1](https://www.ncbi.nlm.nih.gov/protein/QXL90447.1?report=genbank&log$=prottop&blast_rank=1&RID=AZKCERDS016) | - |
| 169 | - | 1,04,669..1,04,875 | 207 | 68 | 8.9 | 6.7 | ATG | Hypothetical protein (Salmonella phage SSE121) | 100 | 100 | 2e-41 | [YP_009148861.1](https://www.ncbi.nlm.nih.gov/protein/YP_009148861.1?report=genbank&log$=prottop&blast_rank=1&RID=B05R391X016) | - |
| 170 | - | 1,04,946.. 1,05,305 | 360 | 119 | 13.7 | 5.9 | ATG | Hypothetical protein (Salmonella phage SSE121) | 100 | 100 | 2e-80 | [YP_009148862.1](https://www.ncbi.nlm.nih.gov/protein/YP_009148862.1?report=genbank&log$=prottop&blast_rank=1&RID=AZKR69WZ016) | - |
| 171 | - | 1,05,307.. 1,05,504 | 198 | 65 | 7.2 | 4.9 | ATG | Hypothetical protein (Salmonella phage PVPSE1) | 100 | 100 | 2e-39 | [YP_004893826.1](https://www.ncbi.nlm.nih.gov/protein/YP_004893826.1?report=genbank&log$=prottop&blast_rank=1&RID=AZKXZS48016) | DUF2158 super family ([cl02293](https://www.ncbi.nlm.nih.gov/Structure/cdd/cddsrv.cgi?ascbin=8&maxaln=10&seltype=2&uid=cl02293)), [Protein of unknown function DUF2158](https://www.ebi.ac.uk/interpro/entry/interpro/IPR019226)(IPR019226) |
| 172 | - | 1,05,524.. 1,06,366 | 843 | 280 | 27.5 | 7.6 | ATG | Hypothetical protein (Salmonella phage NINP13076) | 99.64 | 100 | 0 | [QXL90443.1](https://www.ncbi.nlm.nih.gov/protein/QXL90443.1?report=genbank&log$=prottop&blast_rank=1&RID=AZM6KATD013) | - |
| 173 | - | 1,06,406.. 1,09,111 | 2706 | 901 | 103.7 | 6.9 | TTG | DNA-directed DNA polymerase (Salmonella phage NINP13076) | 99.22 | 100 | 0 | [QXL90442.1](https://www.ncbi.nlm.nih.gov/protein/QXL90442.1?report=genbank&log$=prottop&blast_rank=1&RID=AZMGYMYW016) | DNA_pol_A super family  [(cl02626](https://www.ncbi.nlm.nih.gov/Structure/cdd/cddsrv.cgi?ascbin=8&maxaln=10&seltype=2&uid=cl02626)),  DNA-directed DNA polymerase, family A, palm domain (IPR001098) |
| 174 | - | 1,09,177.. 1,09,551 | 375 | 124 | 14.6 | 9.2 | ATG | Hypothetical protein (Salmonella phage NINP13076) | 99.9 | 100 | 1e-84 | [QXL90441.1](https://www.ncbi.nlm.nih.gov/protein/QXL90441.1?report=genbank&log$=prottop&blast_rank=1&RID=B045UZ0G01N) | - |
| 175 | - | 1,09,610.. 1,11,664 | 2055 | 684 | 76.5 | 5.5 | ATG | AAA family ATPase (Salmonella phage SSE121) | 99.55 | 97 | 0 | [YP_009148870.1](https://www.ncbi.nlm.nih.gov/protein/YP_009148870.1?report=genbank&log$=prottop&blast_rank=1&RID=B053BK4R016) | RecA-like_Gp4D_helicase ([cd19483](https://www.ncbi.nlm.nih.gov/Structure/cdd/cddsrv.cgi?ascbin=8&maxaln=10&seltype=2&uid=cd19483)), ABC_ATPase ([cd00267](https://www.ncbi.nlm.nih.gov/Structure/cdd/cddsrv.cgi?ascbin=8&maxaln=10&seltype=2&uid=cd00267)), DnaG super family ([cl43116](https://www.ncbi.nlm.nih.gov/Structure/cdd/cddsrv.cgi?ascbin=8&maxaln=10&seltype=2&uid=cl43116)), [DNA helicase, DnaB-like, C-terminal](https://www.ebi.ac.uk/interpro/entry/InterPro/IPR007694/) (IPR007694) |
| 176 | - | 1,11,661.. 1,12,191 | 531 | 176 | 19.8 | 9.8 | ATG | HNH ENdonuclease (Salmonella phage PVPSE1) | 99.43 | 100 | 6e-127 | [YP_004893819.1](https://www.ncbi.nlm.nih.gov/protein/YP_004893819.1?report=genbank&log$=prottop&blast_rank=1&RID=B05YEF5A016) | HNH nuclease (IPR003615), HNHc super family ([cl00083](https://www.ncbi.nlm.nih.gov/Structure/cdd/cddsrv.cgi?ascbin=8&maxaln=10&seltype=2&uid=cl00083)) |
| 177 | - | 1,12,201.. 1,12,347 | 147 | 48 | 5.4 | 8.3 | TTG | Hypothetical protein (Salmonella phage PVPSE1) | 97.92 | 100 | 1e-25 | [YP_004893818.1](https://www.ncbi.nlm.nih.gov/protein/YP_004893818.1?report=genbank&log$=prottop&blast_rank=1&RID=B06FKG9D013) | - |
| 178 | - | 1,12,356.. 1,12,862 | 507 | 168 | 18.4 | 6.7 | GTG | Hypothetical protein (Salmonella phage PVPSE1) | 99.4 | 100 | 1e-120 | [YP_004893817.1](https://www.ncbi.nlm.nih.gov/protein/YP_004893817.1?report=genbank&log$=prottop&blast_rank=1&RID=B06R4C9T016) | LamG-like jellyroll fold (IPR006558),  LamG super family ([cl22861](https://www.ncbi.nlm.nih.gov/Structure/cdd/cddsrv.cgi?ascbin=8&maxaln=10&seltype=2&uid=cl22861)) |
| 179 | - | 1,13,033.. 1,13,725 | 693 | 230 | 25.7 | 9.0 | ATG | DNA methytransferase (Salmonella phage SSE121) | 100 | 100 | 6e-171 | [YP_009148874.1](https://www.ncbi.nlm.nih.gov/protein/YP_009148874.1?report=genbank&log$=prottop&blast_rank=1&RID=B06TDWGX013) | - |
| 180 | - | 1,13,733.. 1,13,987 | 255 | 84 | 9.2 | 4.8 | ATG | DNA ligase (Salmonella phage PVPSE1) | 95.24 | 100 | 3e-51 | [YP_004893815.1](https://www.ncbi.nlm.nih.gov/protein/YP_004893815.1?report=genbank&log$=prottop&blast_rank=1&RID=B06WU58C013) | - |
| 181 | - | 1,13,987.. 1,14,268 | 282 | 93 | 11.1 | 4.8 | ATG | Hypothetical protein (Salmonella phage PVPSE1) | 100 | 100 | 1e-62 | [YP_004893814.1](https://www.ncbi.nlm.nih.gov/protein/YP_004893814.1?report=genbank&log$=prottop&blast_rank=1&RID=B078YZSX016) |  |
| 182 | - | 1,14,261.. 1,14,590 | 330 | 109 | 12.4 | 4.5 | ATG | DNA ligase (Salmonella phage PVPSE1) | 99.08 | 100 | 3e-73 | [YP_004893813.1](https://www.ncbi.nlm.nih.gov/protein/YP_004893813.1?report=genbank&log$=prottop&blast_rank=1&RID=B07BU295013) |  |
| 183 | - | 1,14,571.. 1,14,756 | 186 | 61 | 7.2 | 6.2 | ATG | Hypothetical protein (Salmonella phage PVPSE1) | 100 | 100 | 9e-35 | [YP_004893812.1](https://www.ncbi.nlm.nih.gov/protein/YP_004893812.1?report=genbank&log$=prottop&blast_rank=1&RID=B08JZ19D01N) | - |
| 184 | - | 1,14,746.. 1,15,180 | 435 | 144 | 16.5 | 9.4 | ATG | Hypothetical protein (Salmonella phage PVPSE1) | 100 | 100 | 5e-103 | [YP_004893811.1](https://www.ncbi.nlm.nih.gov/protein/YP_004893811.1?report=genbank&log$=prottop&blast_rank=1&RID=B08S9VJX016) | - |
| 185 | - | 1,15,192.. 1,16,496 | 1305 | 434 | 48.4 | 8.6 | ATG | Helicase (Salmonella phage PVPSE1) | 100 | 100 | 0 | [YP_004893810.1](https://www.ncbi.nlm.nih.gov/protein/YP_004893810.1?report=genbank&log$=prottop&blast_rank=1&RID=B08Z447Y013) | DEAD-like_helicase_N super family ([cl28899](https://www.ncbi.nlm.nih.gov/Structure/cdd/cddsrv.cgi?ascbin=8&maxaln=10&seltype=2&uid=cl28899)), RecD super family ([cl33920](https://www.ncbi.nlm.nih.gov/Structure/cdd/cddsrv.cgi?ascbin=8&maxaln=10&seltype=2&uid=cl33920)), [DNA helicase Pif1-like](https://www.ebi.ac.uk/interpro/entry/interpro/IPR010285)(IPR010285) |
| 186 | - | 1,16,496.. 1,17,734 | 1239 | 412 | 45.7 | 5.0 | ATG | rIIB lysis inhibitor (Salmonella phage NINP13076) | 99.75 | 97 | 0 | [QXL90426.1](https://www.ncbi.nlm.nih.gov/protein/QXL90426.1?report=genbank&log$=prottop&blast_rank=1&RID=B098N1W6016) | - |
| 187 | - | 1,17,731.. 1,19,725 | 1995 | 664 | 76.1 | 6.8 | ATG | rIIA lysis inhibitor (Salmonella phage NINP13076) | 100 | 100 | 0 | [QXL90427.1](https://www.ncbi.nlm.nih.gov/protein/QXL90427.1?report=genbank&log$=prottop&blast_rank=1&RID=B09CJE8M016) | [Histidine kinase/HSP90-like ATPase](https://www.ebi.ac.uk/interpro/entry/InterPro/IPR003594/) (IPR036890) |
| 188 | - | 1,19,737.. 1,19,910 | 174 | 57 | 6.6 | 9.7 | ATG | Hypothetical protein (Salmonella phage NINP13076) | 100 | 100 | 4e-33 | [QXL90428.1](https://www.ncbi.nlm.nih.gov/protein/QXL90428.1?report=genbank&log$=prottop&blast_rank=1&RID=B09RB6YA016) | - |
| 189 | - | 1,19,915.. 1,20,445 | 531 | 176 | 20.2 | 9.7 | ATG | cell wall hydrolase (Salmonella phage NINP13076) | 99.43 | 100 | 4e-129 | [QXL90429.1](https://www.ncbi.nlm.nih.gov/protein/QXL90429.1?report=genbank&log$=prottop&blast_rank=1&RID=B09YJKPX016) | Hydrolase_2 super family ([cl38231](https://www.ncbi.nlm.nih.gov/Structure/cdd/cddsrv.cgi?ascbin=8&maxaln=10&seltype=2&uid=cl38231)), Cell wall hydrolase, SleB (IPR011105), |
| 190 | - | 1,20,488.. 1,20,892 | 405 | 134 | 14.2 | 5.5 | ATG | Hypothetical protein (Salmonella phage NINP13076) | 100 | 100 | 4e-87 | [QXL90430.1](https://www.ncbi.nlm.nih.gov/protein/QXL90430.1?report=genbank&log$=prottop&blast_rank=1&RID=B0A80KJU013) | DUF4097 super family ([cl44625](https://www.ncbi.nlm.nih.gov/Structure/cdd/cddsrv.cgi?ascbin=8&maxaln=10&seltype=2&uid=cl44625)) |
| 191 | - | 1,20,879.. 1,21,076 | 198 | 65 | 7.2 | 4.7 | ATG | Hypothetical protein (Salmonella phage NINP13076) | 100 | 100 | 3e-39 | [QXL90431.1](https://www.ncbi.nlm.nih.gov/protein/QXL90431.1?report=genbank&log$=prottop&blast_rank=1&RID=B0AC1YEZ016) | - |
| 192 | - | 1,21,236.. 1,22,273 | 1038 | 345 | 38.4 | 5.4 | TTG | RNA ligase 2 (Salmonella phage NINP13076) | 99.71 | 100 | 0 | [QXL90433.1](https://www.ncbi.nlm.nih.gov/protein/QXL90433.1?report=genbank&log$=prottop&blast_rank=1&RID=B0AJAC3S016) | Adenylation_DNA_ligase_like super family ([cl12015](https://www.ncbi.nlm.nih.gov/Structure/cdd/cddsrv.cgi?ascbin=8&maxaln=10&seltype=2&uid=cl12015)), RNA ligase domain, REL/Rln2 (IPR021122) |
| 193 | - | 1,22,266.. 1,22,631 | 366 | 121 | 13.2 | 6.0 | ATG | Hypothetical protein (Salmonella phage NINP13076) | 100 | 100 | 8e-86 | [QXL90434.1](https://www.ncbi.nlm.nih.gov/protein/QXL90434.1?report=genbank&log$=prottop&blast_rank=1&RID=B0ANKD92016) | - |
| 194 | - | 1,22,618.. 1,22,848 | 231 | 76 | 8.5 | 8.7 | ATG | Hypothetical protein (Salmonella phage NINP13076) | 100 | 100 | 3e-47 | [QXL90435.1](https://www.ncbi.nlm.nih.gov/protein/QXL90435.1?report=genbank&log$=prottop&blast_rank=1&RID=B0B28ST7013) | - |
| 195 | - | 1,22,892.. 1,23,041 | 150 | 49 | 5.4 | 6.5 | ATG | Hypothetical protein (Salmonella phage NINP13076) | 100 | 100 | 3e-28 | [QXL90436.1](https://www.ncbi.nlm.nih.gov/protein/QXL90436.1?report=genbank&log$=prottop&blast_rank=1&RID=B0B74GU5013) | - |
| 196 | - | 1,23,041.. 1,23,253 | 213 | 70 | 7.9 | 4.6 | ATG | Hypothetical protein (Salmonella phage NINP13076) | 100 | 100 | 7e-44 | [QXL90437.1](https://www.ncbi.nlm.nih.gov/protein/QXL90437.1?report=genbank&log$=prottop&blast_rank=1&RID=B0B944DC013) | - |
| 197 | - | 1,23,250.. 1,23,438 | 189 | 62 | 7.3 | 7.9 | ATG | Membrane protein (Salmonella phage PVPSE1) | 100 | 100 | 7e-36 | [YP_004894040.1](https://www.ncbi.nlm.nih.gov/protein/YP_004894040.1?report=genbank&log$=prottop&blast_rank=1&RID=B0BFVHS501N) | - |
| 198 | - | 1,23,435.. 1,23,929 | 495 | 164 | 18 | 8.2 | ATG | Hypothetical protein (Salmonella phage 40) | 97.55 | 99 | 6e-114 | [AKJ73532.1](https://www.ncbi.nlm.nih.gov/protein/AKJ73532.1?report=genbank&log$=prottop&blast_rank=1&RID=B0BK2S4E01N) | Macro_SF super family ([cl00019](https://www.ncbi.nlm.nih.gov/Structure/cdd/cddsrv.cgi?ascbin=8&maxaln=10&seltype=2&uid=cl00019)), Macro domain  (IPR002589) |
| 199 | - | 1,23,926.. 1,24,435 | 510 | 169 | 19.2 | 7.8 | ATG | Hypothetical protein (Salmonella phage NINP13076) | 100 | 100 | 4e-118 | [QXL90549.1](https://www.ncbi.nlm.nih.gov/protein/QXL90549.1?report=genbank&log$=prottop&blast_rank=1&RID=B0BWXWKX016) | - |
| 200 | - | 1,24,435.. 1,24,611 | 177 | 58 | 7.0 | 9.3 | ATG | Hypothetical protein (Salmonella phage NINP13076) | 100 | 100 | 2e-35 | [QXL90548.1](https://www.ncbi.nlm.nih.gov/protein/QXL90548.1?report=genbank&log$=prottop&blast_rank=1&RID=B0C3PG16013) | - |
| 201 | - | 1,24,611.. 1,24,814 | 204 | 67 | 7.3 | 6.6 | ATG | Hypothetical protein (Salmonella phage NINP13076) | 100 | 100 | 1e-42 | [QXL90547.1](https://www.ncbi.nlm.nih.gov/protein/QXL90547.1?report=genbank&log$=prottop&blast_rank=1&RID=B0E06G8U016) | - |
| 202 | - | 1,24,811.. 1,24,996 | 186 | 61 | 6.6 | 8.5 | ATG | Hypothetical protein (Salmonella phage NINP13076) | 100 | 100 | 4e-35 | [QXL90546.1](https://www.ncbi.nlm.nih.gov/protein/QXL90546.1?report=genbank&log$=prottop&blast_rank=1&RID=B0E47SAJ016) | - |
| 203 | - | 1,24,989.. 1,25,186 | 198 | 65 | 7.7 | 5.0 | ATG | Hypothetical protein (Salmonella phage NINP13076) | 100 | 100 | 1e-40 | [QXL90545.1](https://www.ncbi.nlm.nih.gov/protein/QXL90545.1?report=genbank&log$=prottop&blast_rank=1&RID=B0E6MBC8013) | - |
| 204 | - | 1,25,251.. 1,26,459 | 1209 | 402 | 45.1 | 5.1 | ATG | Hypothetical protein (Salmonella phage NINP13076) | 100 | 92 | 0 | [QXL90544.1](https://www.ncbi.nlm.nih.gov/protein/QXL90544.1?report=genbank&log$=prottop&blast_rank=1&RID=B0EGCZH7013) | P-loop_NTPase super family ([cl38936](https://www.ncbi.nlm.nih.gov/Structure/cdd/cddsrv.cgi?ascbin=8&maxaln=10&seltype=2&uid=cl38936)), P-loop containing nucleoside triphosphate hydrolase (IPR027417) |
| 205 | - | 1,26,446.. 1,26,631 | 186 | 61 | 7.1 | 6.2 | ATG | Hypothetical protein (Salmonella phage NINP13076) | 99.46 | 98.36 | 3e-35 | [QXL90558.1](https://www.ncbi.nlm.nih.gov/protein/QXL90558.1?report=genbank&log$=prottop&blast_rank=1&RID=B0ENEVZZ013) | - |
| 206 | - | 1,26,621.. 1,26,860 | 240 | 79 | 9.2 | 5.2 | GTG | Hypothetical protein (Salmonella phage NINP13076) | 99.53 | 90.79 | 2e-43 | [QXL90557.1](https://www.ncbi.nlm.nih.gov/protein/QXL90557.1?report=genbank&log$=prottop&blast_rank=1&RID=B0EV8PMG013) | - |
| 207 | - | 1,26,857.. 1,27,237 | 381 | 126 | 15.1 | 4.8 | ATG | Hypothetical protein (Cronobacter phage EspYZU15) | 98.41 | 100 | 1e-87 | [WAK43713.1](https://www.ncbi.nlm.nih.gov/protein/WAK43713.1?report=genbank&log$=prottop&blast_rank=1&RID=B0F1WHP4013) | - |
| 208 | - | 1,27,230.. 1,27,739 | 510 | 169 | 19.7 | 9.5 | ATG | Hypothetical protein (Salmonella phage NINP13076) | 100 | 100 | 2e-122 | [QXL90561.1](https://www.ncbi.nlm.nih.gov/protein/QXL90561.1?report=genbank&log$=prottop&blast_rank=1&RID=B0F7NX1T013) | CcmH_N super family ([cl01179](https://www.ncbi.nlm.nih.gov/Structure/cdd/cddsrv.cgi?ascbin=8&maxaln=10&seltype=2&uid=cl01179)) |
| 209 | - | 1,27,736..1,28,242 | 507 | 168 | 19.0 | 7.9 | ATG | Hypothetical protein (Salmonella phage 40) | 85.89 | 97 | 5e-98 | [AKJ73549.1](https://www.ncbi.nlm.nih.gov/protein/AKJ73549.1?report=genbank&log$=prottop&blast_rank=1&RID=B0FWYC6501N) | GUANOSINE-3',5'-BIS(DIPHOSPHATE) 3'-PYROPHOSPHOHYDROLASE MESH1 (PTHR46246), NT_Pol-beta-like super family ([cl11966](https://www.ncbi.nlm.nih.gov/Structure/cdd/cddsrv.cgi?ascbin=8&maxaln=10&seltype=2&uid=cl11966)) |
| 210 | - | 1,28,206.. 1,29,237 | 1032 | 343 | 40.6 | 5.9 | ATG | Hypothetical protein (Salmonella phage NINP13076) | 100 | 100 | 0 | [QXL90449.1](https://www.ncbi.nlm.nih.gov/protein/QXL90449.1?report=genbank&log$=prottop&blast_rank=1&RID=B1A5XV7E016) | - |
| 211 | + | 1,29,363..1,30,220 | 858 | 285 | 32.5 | 6.1 | ATG | Hypothetical protein (Salmonella phage NINP13076) | 100 | 100 | 0 | [QXL90450.1](https://www.ncbi.nlm.nih.gov/protein/QXL90450.1?report=genbank&log$=prottop&blast_rank=1&RID=B1AB533W013) | - |
| 212 | + | 1,30,220.. 1,30,477 | 258 | 85 | 9.7 | 5.6 | ATG | Hypothetical protein (Salmonella phage NINP13076) | 100 | 100 | 9e-55 | [QXL90451.1](https://www.ncbi.nlm.nih.gov/protein/QXL90451.1?report=genbank&log$=prottop&blast_rank=1&RID=B1AG0PX2013) | - |
| 213 | + | 1,30,474..1,31,964 | 1491 | 496 | 56.9 | 8.8 | ATG | Hypothetical protein (Salmonella phage NINP13076) | 99.80 | 100 | 0 | [QXL90452.1](https://www.ncbi.nlm.nih.gov/protein/QXL90452.1?report=genbank&log$=prottop&blast_rank=1&RID=B1AKXA02013) | Putative metallopeptidase domain (IPR025154)  DUF2201_N super family ([cl37600](https://www.ncbi.nlm.nih.gov/Structure/cdd/cddsrv.cgi?ascbin=8&maxaln=10&seltype=2&uid=cl37600)) |
| 214 | + | 1,31,948.. 1,32,334 | 387 | 128 | 14.9 | 5.2 | ATG | Hypothetical protein (Salmonella phage GEC_vB_MG) | 99.22 | 100 | 4e-99 | [QPI14800.1](https://www.ncbi.nlm.nih.gov/protein/QPI14800.1?report=genbank&log$=prottop&blast_rank=1&RID=B1BU9Z9P016) | - |
| 215 | + | 1,32,334..1,32,630.. | 297 | 98 | 11.4 | 9.2 | ATG | Hypothetical protein (Salmonella phage NINP13076) | 100 | 100 | 6e-66 | [QXL90454.1](https://www.ncbi.nlm.nih.gov/protein/QXL90454.1?report=genbank&log$=prottop&blast_rank=1&RID=B1BYXECH016) | HTH super family ([cl21459](https://www.ncbi.nlm.nih.gov/Structure/cdd/cddsrv.cgi?ascbin=8&maxaln=10&seltype=2&uid=cl21459)), Winged helix-like DNA-binding domain superfamily  (IPR036388) |
| 216 | + | 1,32,905..1,33,273 | 369 | 122 | 13.4 | 5.7 | ATG | Hypothetical protein (Cronobacter phage EspYZU15) | 83.87 | 100 | 6e-74 | [UGV21654.1](https://www.ncbi.nlm.nih.gov/protein/UGV21654.1?report=genbank&log$=prottop&blast_rank=1&RID=B1C71CFC016) | - |
| 217 | + | 1,33,482..1,33,270 | 213 | 70 | 7.9 | 7.6 | ATG | Hypothetical protein (Salmonella phage NINP13076) | 100 | 100 | 1e-43 | [QXL90458.1](https://www.ncbi.nlm.nih.gov/protein/QXL90458.1?report=genbank&log$=prottop&blast_rank=1&RID=B1C9PUPV013) | - |
| 218 | + | 1,33,494..1,34,135 | 642 | 213 | 23.9 | 5.5 | ATG | Hypothetical protein (Salmonella phage NINP13076) | 100 | 88 | 1e-135 | [QXL90579.1](https://www.ncbi.nlm.nih.gov/protein/QXL90579.1?report=genbank&log$=prottop&blast_rank=1&RID=B1CHJJ2P013) | - |
| 219 | + | 1,34,132..1,34,494 | 363 | 120 | 13.5 | 5.0 | ATG | Hypothetical protein (Salmonella phage NINP13076) | 100 | 100 | 2e-82 | [QXL90578.1](https://www.ncbi.nlm.nih.gov/protein/QXL90578.1?report=genbank&log$=prottop&blast_rank=1&RID=B1CNM38A013) | NTP-PPase super family ([cl16941](https://www.ncbi.nlm.nih.gov/Structure/cdd/cddsrv.cgi?ascbin=8&maxaln=10&seltype=2&uid=cl16941)), all-alpha NTP pyrophosphatases (SSF101386) |
| 220 | + | 1,34,481..1,34,870 | 390 | 129 | 14.9 | 4.9 | GTG | Putative carbohydrate binding domain protein (Cronobacter phage EspYZU15) | 89.15 | 100 | 1e-80 | [WAK43698.1](https://www.ncbi.nlm.nih.gov/protein/WAK43698.1?report=genbank&log$=prottop&blast_rank=1&RID=B1D0PG5R01N) | - |
| 221 | + | 1,34,830..1,35,243 | 414 | 137 | 15.9 | 8.8 | ATG | Hypothetical protein (Salmonella phage NINP13076) | 100 | 100 | 9e-98 | [QXL90593.1](https://www.ncbi.nlm.nih.gov/protein/QXL90593.1?report=genbank&log$=prottop&blast_rank=1&RID=B1D89ZPK01N) | - |
| 222 | + | 1,35,240..1,35,389 | 150 | 49 | 6.1 | 9.4 | ATG | Hypothetical protein (Salmonella phage 40) | 87.76 | 100 | 1e-22 | [AKJ73566.1](https://www.ncbi.nlm.nih.gov/protein/AKJ73566.1?report=genbank&log$=prottop&blast_rank=1&RID=B1DAZTT1013) | - |
| 223 | + | 1,35,509..1,36,351 | 843 | 280 | 32.2 | 4.6 | ATG | Hypothetical protein (Salmonella phage NINP13076) | 100 | 100 | 0 | [QXL90424.1](https://www.ncbi.nlm.nih.gov/protein/QXL90424.1?report=genbank&log$=prottop&blast_rank=1&RID=B1GV4R71013)1 | - |
| 224 | - | 1,36,640..1,36,759 | 120 | 39 | 4.9 | 6.5 | TTG | Hypothetical protein | - | - | - | - | - |
| 225 | - | 1,37,255..1,37,464 | 210 | 69 | 7.7 | 9.5 | ATG | Hypothetical protein (Salmonella phage NINP13076) | 100 | 100 | 1e-42 | [QXL90418.1](https://www.ncbi.nlm.nih.gov/protein/QXL90418.1?report=genbank&log$=prottop&blast_rank=1&RID=B1H6BN1Y016) | - |
| 226 | + | 1,37,654..1,38,184 | 531 | 176 | 19.2 | 4.1 | ATG | Hypothetical protein (Salmonella phage NINP13076) | 100 | 100 | 5e-125 | [QXL90416.1](https://www.ncbi.nlm.nih.gov/protein/QXL90416.1?report=genbank&log$=prottop&blast_rank=1&RID=B1HAYZPJ016) | - |
| 227 | + | 1,38,257..1,38,496 | 240 | 79 | 9.0 | 9.6 | ATG | Hypothetical protein (Salmonella phage NINP13076) | 100 | 100 | 8e-51 | [QXL90415.1](https://www.ncbi.nlm.nih.gov/protein/QXL90415.1?report=genbank&log$=prottop&blast_rank=1&RID=B1HSZY2D016) | - |
| 228 | + | 1,38,497..1,38,913 | 417 | 138 | 15.0 | 10.3 | TTG | Hypothetical protein (Salmonella phage NINP13076) | 100 | 79 | 8e-73 | [QXL90414.1](https://www.ncbi.nlm.nih.gov/protein/QXL90414.1?report=genbank&log$=prottop&blast_rank=1&RID=B40MPGC1013) | - |
| 229 | + | 1,38,922..1,39,332 | 411 | 136 | 15.7 | 9.0 | TTG | Hypothetical protein (Cronobacter phage EspYZU15) | 94.16 | 100 | 4e-88 | [WAK43691.1](https://www.ncbi.nlm.nih.gov/protein/WAK43691.1?report=genbank&log$=prottop&blast_rank=1&RID=B40U1JJS01N) | - |
| 230 | + | 1,39,411..1,39,809 | 399 | 132 | 15.0 | 9.5 | ATG | Hypothetical protein (Salmonella phage NINP13076) | 100 | 100 | 5e-93 | [QXL90412.1](https://www.ncbi.nlm.nih.gov/protein/QXL90412.1?report=genbank&log$=prottop&blast_rank=1&RID=B1HVHSGE016) | - |
| 231 | + | 1,39,811..1,39,996 | 186 | 61 | 6.8 | 6.0 | ATG | Hypothetical protein (Salmonella phage NINP13076) | 98.36 | 100 | 4e-34 | [QXL90411.1](https://www.ncbi.nlm.nih.gov/protein/QXL90411.1?report=genbank&log$=prottop&blast_rank=1&RID=B1J1VVEB01N) | - |
| 232 | + | 1,40,092..1,40,658 | 567 | 188 | 21.3 | 5.1 | ATG | Hypothetical protein (Salmonella phage NINP13076) | 100 | 100 | 8e-138 | [QXL90409.1](https://www.ncbi.nlm.nih.gov/protein/QXL90409.1?report=genbank&log$=prottop&blast_rank=1&RID=B1JFSE56013) | - |
| 233 | + | 1,40,661..1,41,008 | 348 | 115 | 13.7 | 9.6 | TTG | Hypothetical protein (Cronobacter phage EspYZU15) | 98.26 | 100 | 8e-77 | [UGV21659.1](https://www.ncbi.nlm.nih.gov/protein/UGV21659.1?report=genbank&log$=prottop&blast_rank=1&RID=B1JRYTGY013) | - |
| 234 | + | 1,41,025..1,41,228 | 204 | 67 | 8.1 | 10.0 | ATG | Hypothetical protein (Salmonella phage PVPSE1) | 97.01 | 100 | 1e-38 | [YP_004893999.1](https://www.ncbi.nlm.nih.gov/protein/YP_004893999.1?report=genbank&log$=prottop&blast_rank=1&RID=B1K0BM66013) | [Bacteriophage T7, Gp1.1](https://www.ebi.ac.uk/interpro/entry/interpro/IPR013232)(IPR013232) |
| 235 | + | 1,41,570..1,41,797 | 228 | 75 | 8.4 | 4.0 | ATG | Hypothetical protein (Salmonella phage NINP13076) | 100 | 100 | 1e-47 | [QXL90404.1](https://www.ncbi.nlm.nih.gov/protein/QXL90404.1?report=genbank&log$=prottop&blast_rank=1&RID=B1KA3W42013) | - |
| 236 | + | 1,41,880..1,42,164 | 285 | 94 | 11 | 10 | ATG | Hypothetical protein (Salmonella phage NINP13076) | 98.94 | 100 | 5e-62 | [QXL90403.1](https://www.ncbi.nlm.nih.gov/protein/QXL90403.1?report=genbank&log$=prottop&blast_rank=1&RID=B20S8AHP013) | - |
| 237 | + | 1,42,211..1,42,342 | 132 | 43 | 4.8 | 4.5 | ATG | Hypothetical protein (Salmonella phage NINP13076) | 100 | 100 | 1e-21 | [QXL90402.1](https://www.ncbi.nlm.nih.gov/protein/QXL90402.1?report=genbank&log$=prottop&blast_rank=1&RID=B418X0N0013) | - |
| 238 | + | 1,42,345..1,42,563 | 219 | 72 | 8.2 | 6.7 | ATG | Hypothetical protein (Salmonella phage NINP13076) | 100 | 100 | 5e-45 | [QXL90401.1](https://www.ncbi.nlm.nih.gov/protein/QXL90401.1?report=genbank&log$=prottop&blast_rank=1&RID=B20X8EHT013) | - |
| 239 | + | 1,42,572..1,42,844 | 273 | 90 | 10.4 | 4.0 | ATG | Hypothetical protein (Salmonella phage NINP13076) | 98.89 | 100 | 2e-57 | [QXL90400.1](https://www.ncbi.nlm.nih.gov/protein/QXL90400.1?report=genbank&log$=prottop&blast_rank=1&RID=B253XKNG013) | - |
| 240 | + | 1,42,854..1,43,039 | 186 | 61 | 6.7 | 3.6 | ATG | Hypothetical protein (Salmonella phage NINP13076) | 100 | 100 | 6e-36 | [QXL90399.1](https://www.ncbi.nlm.nih.gov/protein/QXL90399.1?report=genbank&log$=prottop&blast_rank=1&RID=B411KCUM01N) | - |
| 241 | + | 1,43,125..1,43,241 | 117 | 38 | 4.5 | 4.8 | ATG | Hypothetical protein (Salmonella phage NINP13076) | 100 | 100 | 7e-19 | [QXL90398.1](https://www.ncbi.nlm.nih.gov/protein/QXL90398.1?report=genbank&log$=prottop&blast_rank=1&RID=B25BEK9J016) | - |
| 242 | + | 1,43,257..1,43,598 | 342 | 113 | 12.7 | 5.0 | ATG | Hypothetical protein (Salmonella phage NINP13076) | 100 | 100 | 4e-77 | [QXL90397.1](https://www.ncbi.nlm.nih.gov/protein/QXL90397.1?report=genbank&log$=prottop&blast_rank=1&RID=B25UEKGP013) | - |
| 243 | + | 1,43,650..1,43,832 | 183 | 60 | 6.8 | 9.3 | ATG | Hypothetical protein (Salmonella phage NINP13076) | 100 | 100 | 1e-34 | [QXL90396.1](https://www.ncbi.nlm.nih.gov/protein/QXL90396.1?report=genbank&log$=prottop&blast_rank=1&RID=B2623KS4013) | - |
| 244 | + | 1,43,845..1,44,078 | 234 | 77 | 8.6 | 10.0 | ATG | Hypothetical protein (Salmonella phage NINP13076) | 100 | 100 | 1e-48 | [QXL90395.1](https://www.ncbi.nlm.nih.gov/protein/QXL90395.1?report=genbank&log$=prottop&blast_rank=1&RID=B267Z9Y1013) | - |
| 245 | + | 1,44,081..1,44,413 | 333 | 110 | 12.6 | 4.06 | ATG | Hypothetical protein (Salmonella phage NINP13076) | 100 | 100 | 2e-75 | [QXL90394.1](https://www.ncbi.nlm.nih.gov/protein/QXL90394.1?report=genbank&log$=prottop&blast_rank=1&RID=B26D83X1016) | - |
| 246 | + | 1,44,500..1,44,760 | 261 | 86 | 9.6 | 8.7 | ATG | Hypothetical protein (Salmonella phage NINP13076) | 100 | 100 | 4e-54 | [QXL90393.1](https://www.ncbi.nlm.nih.gov/protein/QXL90393.1?report=genbank&log$=prottop&blast_rank=1&RID=B280Z2MM013) | - |
| 247 | + | 1,44,799..1,45,029 | 231 | 76 | 8.5 | 9.7 | ATG | Hypothetical protein (Salmonella phage NINP13076) | 100 | 100 | 2e-48 | [QXL90392.1](https://www.ncbi.nlm.nih.gov/protein/QXL90392.1?report=genbank&log$=prottop&blast_rank=1&RID=B285WRRH016) | - |
| 248 | + | 1,45,026..1,45,253 | 228 | 75 | 8.7 | 4.6 | ATG | Hypothetical protein (Salmonella phage NINP13076) | 100 | 100 | 8e-47 | [QXL90391.1](https://www.ncbi.nlm.nih.gov/protein/QXL90391.1?report=genbank&log$=prottop&blast_rank=1&RID=B28ERM0K01N) | - |
| 249 | + | 1,45,358..1,45,807 | 450 | 149 | 17.3 | 5.6 | ATG | Hypothetical protein (Salmonella phage NINP13076) | 100 | 100 | 3e-108 | [QXL90390.1](https://www.ncbi.nlm.nih.gov/protein/QXL90390.1?report=genbank&log$=prottop&blast_rank=1&RID=B3ZP94M701N) | - |
| 250 | + | 1,46,004..1,46,273 | 270 | 89 | 9.6 | 7.7 | ATG | Hypothetical protein (Salmonella phage NINP13076) | 100 | 100 | 4e-56 | [QXL90388.1](https://www.ncbi.nlm.nih.gov/protein/QXL90388.1?report=genbank&log$=prottop&blast_rank=1&RID=B3ZVVVBK01N) | - |
