# Supplementary material for: A novel lytic phage infecting MDR Salmonella enterica and its application as effective food biocontrol
Source: Front Microbiol. 2024 Aug 15;15:1387830. doi: 10.3389/fmicb.2024.1387830 (PMC11358711; doi:10.3389/fmicb.2024.1387830)
Supplement: Supplementary file 5 [file Table_3.docx]

**Supplementary Table 3: genomic location and other information of tRNAs present in Salmonella phage phiSalP219**

| **Sr. no.** | **tRNAs** | **Anti-codon** | **Genomic location** | **Length (bp)** | **Sequence** |
| --- | --- | --- | --- | --- | --- |
| 1 | Ile | CAT | 50472-50548 | 77 | GGCTCTGTAGCTTAGTTGGTaAGAGCAGGCGGCTCATAACCGCCAGGtCGCAGGTTCAATCCCTGCCAGAGCCACCA |
| 2 | Lys | CTT | 51379-51454 | 76 | GGGGTATTAACACAGCGGTtAGTGTAGCGGCCTCTTAAGCCGAAGGtCGAAGGTTCGAATCCTTCATGCCCCACCA |
| 3 | Thr | TGT | 53673-53748 | 76 | GCTCGTATAGCTCAGTTGGTAGAGCAACTCCCTTGTAAGGAGAAGGtCGTGGGTTCGAAGCCTACTGCGAGCACCA |
| 4 | Ile | GAT | 53755-53829 | 75 | TCCTCGTTAGCTCAGTTGGTAGAGCAGTCCCACGATTAGGGATCAGCACAGGTTCGATTCCTGTACGAGGAGCCA |
| 5 | Leu | TAA | 54618-54703 | 86 | GCAGGTGTGGTGGAAATGGAGATACACAGGAGACTTAAAATCTCCCGCTTAAATGATTACGAGTTCGAGTCTCGTCACCTGCACCA |
| 6 | Arg | TCT | 54711-54785 | 75 | CGGGGTGTAGTCTAAGGGATAGGCAGCGGTCTTCTAAACCGCTTAATGCTGGTTCGAATCCAGTCACCTCGACCA |
| 7 | Cys | GCA | 54904-54977 | 74 | GCACGGTTGCTGGAGTGGCAACAGCTTCGGCTGCAAACCGAATGTCAGGAGTTCGATTCTTCTACCGTGCTCCA |
| 8 | Tyr | GTA | 55630-55713 | 84 | GGCTCATTGGCAGAACGGTGATTGCAGCGGATTGTAAATCCGTGCCCTTCGGGGTTCCTGGTTCGAATCCAGGGTGGGCCACCA |
| 9 | Ile | GAT | 55721-55796 | 76 | GGGAGTATAGCTCAGTTAGTAGAGCGCTCGACCGATAATCGAGAGGTCGCAGGAGCAAAGCCTGCTACTCCCACCA |
| 10 | Gly | TCC | 55804-55877 | 74 | GCGTCATTAGTGTTAGCGGTCCAGCATCCCGTCCTTCCAAGTCGGTGGCATCGGTTCGAATCCGATATGACGCT |
| 11 | Glu | TTC | 55885-55961 | 77 | GTCCTGTTAGACAAACTGGTAAAGTCACCACCCTTTCAAGGTGGGGTTTGCGGGTTCGATCCCCGCACAGGACGCCA |
| 12 | Gln | TTG | 56051-56127 | 77 | AGGGGGTTAGCATAGCTGGCCTAATGCATCGGGCTTTGAACTCGACATCGGAGGTTCGAATCCTCCACCCCCTGCCA |
| 13 | Ser | GGA | 56241-56332 | 92 | GGTGGAATGGTCGAGCGGTTTAAGACAGCATCTTGGAAAGGTGTCGGCCCCTTAACAGGGGTCCGTAGGTTCAAATCCTACTTCCACCGCCA |
| 14 | Ser | GCT | 56627-56717 | 91 | GGAAGATTAACCCTAATCAGGTAAGGGATCTCTTTGCTAAAGAGACAGTAGCCCCGAAAGGGGTGTGTCAGTTCAAGTCTGACATCTTCCT |
| 15 | Ser | TGA | 56720-56808 | 89 | GGAGAGCAGGACGCATGGTGCGTAATCCGGTTTGAACCCGGACCCATCGTAGCGATACGGTGACAGTTCGATTCTGTTGCTCTCCTCCA |
| 16 | Lys | TTT | 56814-56900 | 87 | GGGTTGGTAGCCAAGCGGTGACGTATGTCGAAGGCACTCGACTTTTAATCGAGAGATCGTTGGGTTCGAATCCCACCCGACCCACCA |
| 17 | Asn | GTT | 56961-57042 | 82 | GACGAGTTGGCCTAGTGGTTGGGCGGCGGCCTGTTAAGCCGTGAGTGAAAACTCAAGGAAGGTTCAAATCCTTCACTCGTCG |
| 18 | Asp | GTC | 57502-57577 | 76 | GGGGTTATAGTTTAGTTGGGCAAAATACTGGCCTGTCACGCCGGAGCCGAGAGTTCGATTCTCTCTAACCTCGCCA |
| 19 | Pro | TGG | 57720-57795 | 76 | CAGTCCGTAGCGCAGTTGGTAGCGTGGGAGCCTTGGATGCTTCGGGTCGCAGGTTCGAGTCCTGCCGGGCTGACCA |
| 20 | Arg | ACG | 57983-58059 | 77 | GCAGGTGTAGCTTAATTGAATAGAGCTTCGTCATACGAAGGCGAAAGATCGGGGTTTGAGTCCCTGCACTTGCTCCA |
| 21 | Ala | TGC | 58124-58202 | 79 | GGGGAATGGGTCTGCTTGGAGTGGACACCTCGCTTGCACCGAGGATAACAGAAGAGTTCGAATCTCTTATTCTCCACCA |
| 22 | Leu | TAG | 58369-58445 | 77 | GGGCGATTGATGGAATTGGTATACGTGCCGTCCTTAGAAGTCGGATTTTGGGGGTTCGAGTCCCCTGTCGCCCACCA |
| 23 | Val | TAC | 58451-58525 | 75 | GCTTCCTTAGTTCAATGGTAGAACGTTGTCTTTACACGGCAAGCGTCGGTGGTTCGATTCCATCAGGAAGTACCA |
| 24 | Phe | GAA | 58532-58607 | 76 | GGGATTGTAACTCAGTTGGTAGAGTGCCTGCCTGAAGAGCAGATGGTCGGTGGTTCGAGTCCACCCGGTCCCGCCA |
| 25 | Met | CAT | 58614-58689 | 76 | TGCGAGCTAGAATTCTGGTGAATTCACGAGTCTCATAAGCTTGTTCAGAGGGGTTCGATTCCGCTGCTCGCAACCA |
