# Supplementary material for: A novel lytic phage infecting MDR Salmonella enterica and its application as effective food biocontrol
Source: Front Microbiol. 2024 Aug 15;15:1387830. doi: 10.3389/fmicb.2024.1387830 (PMC11358711; doi:10.3389/fmicb.2024.1387830)
Supplement: Supplementary file 6 [file Table_4.docx]

Supplementary table 4a: Intergenomic similarity of phage phiSalP219 with 20 other phages

| genome | KR296694.1 | NC_048647.1 | NC_048682.1 | OR352954.1 | KY652726.1 | NC_048656.1 | ON602729.1 | OP296941.1 | NC_022968.1 | NC_019400.1 | PP681140.1 | MW006477.1 | OR413583.1 | MW882934.1 | OP850600.1 | OP819284.1 | OP866730.1 | MZ326168.1 | phiSalP219 | NC_016071.1 | NC_027351.1 |
| --- | --- | --- | --- | --- | --- | --- | --- | --- | --- | --- | --- | --- | --- | --- | --- | --- | --- | --- | --- | --- | --- |
| KR296694.1 | 100 | 26.976 | 25.204 | 25.908 | 26.106 | 26.106 | 26.574 | 26.111 | 37.001 | 36.571 | 37.694 | 38.073 | 37.33 | 37.841 | 38.067 | 38.003 | 38.083 | 38.453 | 41.163 | 40.112 | 40.204 |
| NC_048647.1 | 26.976 | 86.526 | 69.064 | 69.591 | 77.059 | 77.059 | 71.434 | 73.598 | 46.57 | 44.413 | 45.986 | 44.158 | 44.109 | 44.594 | 44.801 | 44.937 | 45.062 | 41.34 | 43.436 | 43.756 | 43.045 |
| NC_048682.1 | 25.204 | 69.064 | 100 | 75.682 | 76.785 | 76.785 | 73.956 | 75.57 | 47.454 | 46.272 | 47.281 | 45.379 | 45.777 | 45.479 | 46.072 | 45.837 | 45.783 | 42.597 | 45.122 | 45.98 | 45.449 |
| OR352954.1 | 25.908 | 69.591 | 75.682 | 100 | 76.17 | 76.17 | 77.513 | 77.347 | 47.099 | 47.267 | 47.402 | 47.594 | 47.577 | 47.481 | 48.17 | 47.912 | 47.984 | 45.267 | 47.123 | 47.824 | 48.032 |
| KY652726.1 | 26.106 | 77.059 | 76.785 | 76.17 | 100 | 100 | 80.623 | 84.487 | 49.887 | 48.001 | 49.199 | 46.024 | 46.633 | 46.949 | 47.096 | 47.314 | 47.428 | 43.758 | 45.85 | 45.703 | 45.459 |
| NC_048656.1 | 26.106 | 77.059 | 76.785 | 76.17 | 100 | 100 | 80.623 | 84.487 | 49.887 | 48.001 | 49.199 | 46.024 | 46.633 | 46.949 | 47.096 | 47.314 | 47.428 | 43.758 | 45.85 | 45.703 | 45.459 |
| ON602729.1 | 26.574 | 71.434 | 73.956 | 77.513 | 80.623 | 80.623 | 100 | 86.635 | 51.521 | 49.898 | 51.047 | 47.331 | 48.782 | 48.683 | 48.993 | 49.213 | 49.142 | 44.545 | 46.649 | 46.857 | 46.6 |
| OP296941.1 | 26.111 | 73.598 | 75.57 | 77.347 | 84.487 | 84.487 | 86.635 | 100 | 50.672 | 49.128 | 49.683 | 46.396 | 47.494 | 47.612 | 48.136 | 48.273 | 48.182 | 43.759 | 46.179 | 46.424 | 46.133 |
| NC_022968.1 | 37.001 | 46.57 | 47.454 | 47.099 | 49.887 | 49.887 | 51.521 | 50.672 | 100 | 81.552 | 82.662 | 64.177 | 67.26 | 66.78 | 66.665 | 66.938 | 66.948 | 59.209 | 62.25 | 64.176 | 63.137 |
| NC_019400.1 | 36.571 | 44.413 | 46.272 | 47.267 | 48.001 | 48.001 | 49.898 | 49.128 | 81.552 | 100 | 91.48 | 64.227 | 66.832 | 66.554 | 66.81 | 67.015 | 67.202 | 59.744 | 61.737 | 64.093 | 62.822 |
| PP681140.1 | 37.694 | 45.986 | 47.281 | 47.402 | 49.199 | 49.199 | 51.047 | 49.683 | 82.662 | 91.48 | 100 | 65.496 | 68.008 | 67.092 | 67.726 | 67.89 | 67.83 | 59.903 | 62.512 | 64.993 | 63.79 |
| MW006477.1 | 38.073 | 44.158 | 45.379 | 47.594 | 46.024 | 46.024 | 47.331 | 46.396 | 64.177 | 64.227 | 65.496 | 100 | 83.33 | 83.194 | 83.083 | 83.565 | 83.317 | 67.397 | 70.329 | 84.979 | 81.939 |
| OR413583.1 | 37.33 | 44.109 | 45.777 | 47.577 | 46.633 | 46.633 | 48.782 | 47.494 | 67.26 | 66.832 | 68.008 | 83.33 | 100 | 88.578 | 87.679 | 87.756 | 87.993 | 66.858 | 68.171 | 77.55 | 74.727 |
| MW882934.1 | 37.841 | 44.594 | 45.479 | 47.481 | 46.949 | 46.949 | 48.683 | 47.612 | 66.78 | 66.554 | 67.092 | 83.194 | 88.578 | 100 | 95.555 | 96.09 | 96.385 | 67.311 | 69.036 | 77.905 | 75.341 |
| OP850600.1 | 38.067 | 44.801 | 46.072 | 48.17 | 47.096 | 47.096 | 48.993 | 48.136 | 66.665 | 66.81 | 67.726 | 83.083 | 87.679 | 95.555 | 100 | 99.375 | 98.953 | 67.41 | 69.009 | 78.058 | 75.516 |
| OP819284.1 | 38.003 | 44.937 | 45.837 | 47.912 | 47.314 | 47.314 | 49.213 | 48.273 | 66.938 | 67.015 | 67.89 | 83.565 | 87.756 | 96.09 | 99.375 | 100 | 99.546 | 67.696 | 69.235 | 78.315 | 75.779 |
| OP866730.1 | 38.083 | 45.062 | 45.783 | 47.984 | 47.428 | 47.428 | 49.142 | 48.182 | 66.948 | 67.202 | 67.83 | 83.317 | 87.993 | 96.385 | 98.953 | 99.546 | 100 | 67.765 | 69.338 | 78.326 | 75.782 |
| MZ326168.1 | 38.453 | 41.34 | 42.597 | 45.267 | 43.758 | 43.758 | 44.545 | 43.759 | 59.209 | 59.744 | 59.903 | 67.397 | 66.858 | 67.311 | 67.41 | 67.696 | 67.765 | 100 | 93.698 | 76.485 | 79.769 |
| phiSalP219 | 41.163 | 43.436 | 45.122 | 47.123 | 45.85 | 45.85 | 46.649 | 46.179 | 62.25 | 61.737 | 62.512 | 70.329 | 68.171 | 69.036 | 69.009 | 69.235 | 69.338 | 93.698 | 100 | 79.991 | 82.411 |
| NC_016071.1 | 40.112 | 43.756 | 45.98 | 47.824 | 45.703 | 45.703 | 46.857 | 46.424 | 64.176 | 64.093 | 64.993 | 84.979 | 77.55 | 77.905 | 78.058 | 78.315 | 78.326 | 76.485 | 79.991 | 100 | 93.676 |
| NC_027351.1 | 40.204 | 43.045 | 45.449 | 48.032 | 45.459 | 45.459 | 46.6 | 46.133 | 63.137 | 62.822 | 63.79 | 81.939 | 74.727 | 75.341 | 75.516 | 75.779 | 75.782 | 79.769 | 82.411 | 93.676 | 100 |

Supplementary Table 4b: Genus and species cluster

| genome | KR296694.1 | NC_048647.1 | NC_048682.1 | OR352954.1 | KY652726.1 | NC_048656.1 | ON602729.1 | OP296941.1 | NC_022968.1 | NC_019400.1 | PP681140.1 | MW006477.1 | OR413583.1 | MW882934.1 | OP850600.1 | OP819284.1 | OP866730.1 | MZ326168.1 | phiSalP219 | NC_016071.1 | NC_027351.1 |
| --- | --- | --- | --- | --- | --- | --- | --- | --- | --- | --- | --- | --- | --- | --- | --- | --- | --- | --- | --- | --- | --- |
| KR296694.1 | 100 | 26.976 | 25.204 | 25.908 | 26.106 | 26.106 | 26.574 | 26.111 | 37.001 | 36.571 | 37.694 | 38.073 | 37.33 | 37.841 | 38.067 | 38.003 | 38.083 | 38.453 | 41.163 | 40.112 | 40.204 |
| NC_048647.1 | 26.976 | 86.526 | 69.064 | 69.591 | 77.059 | 77.059 | 71.434 | 73.598 | 46.57 | 44.413 | 45.986 | 44.158 | 44.109 | 44.594 | 44.801 | 44.937 | 45.062 | 41.34 | 43.436 | 43.756 | 43.045 |
| NC_048682.1 | 25.204 | 69.064 | 100 | 75.682 | 76.785 | 76.785 | 73.956 | 75.57 | 47.454 | 46.272 | 47.281 | 45.379 | 45.777 | 45.479 | 46.072 | 45.837 | 45.783 | 42.597 | 45.122 | 45.98 | 45.449 |
| OR352954.1 | 25.908 | 69.591 | 75.682 | 100 | 76.17 | 76.17 | 77.513 | 77.347 | 47.099 | 47.267 | 47.402 | 47.594 | 47.577 | 47.481 | 48.17 | 47.912 | 47.984 | 45.267 | 47.123 | 47.824 | 48.032 |
| KY652726.1 | 26.106 | 77.059 | 76.785 | 76.17 | 100 | 100 | 80.623 | 84.487 | 49.887 | 48.001 | 49.199 | 46.024 | 46.633 | 46.949 | 47.096 | 47.314 | 47.428 | 43.758 | 45.85 | 45.703 | 45.459 |
| NC_048656.1 | 26.106 | 77.059 | 76.785 | 76.17 | 100 | 100 | 80.623 | 84.487 | 49.887 | 48.001 | 49.199 | 46.024 | 46.633 | 46.949 | 47.096 | 47.314 | 47.428 | 43.758 | 45.85 | 45.703 | 45.459 |
| ON602729.1 | 26.574 | 71.434 | 73.956 | 77.513 | 80.623 | 80.623 | 100 | 86.635 | 51.521 | 49.898 | 51.047 | 47.331 | 48.782 | 48.683 | 48.993 | 49.213 | 49.142 | 44.545 | 46.649 | 46.857 | 46.6 |
| OP296941.1 | 26.111 | 73.598 | 75.57 | 77.347 | 84.487 | 84.487 | 86.635 | 100 | 50.672 | 49.128 | 49.683 | 46.396 | 47.494 | 47.612 | 48.136 | 48.273 | 48.182 | 43.759 | 46.179 | 46.424 | 46.133 |
| NC_022968.1 | 37.001 | 46.57 | 47.454 | 47.099 | 49.887 | 49.887 | 51.521 | 50.672 | 100 | 81.552 | 82.662 | 64.177 | 67.26 | 66.78 | 66.665 | 66.938 | 66.948 | 59.209 | 62.25 | 64.176 | 63.137 |
| NC_019400.1 | 36.571 | 44.413 | 46.272 | 47.267 | 48.001 | 48.001 | 49.898 | 49.128 | 81.552 | 100 | 91.48 | 64.227 | 66.832 | 66.554 | 66.81 | 67.015 | 67.202 | 59.744 | 61.737 | 64.093 | 62.822 |
| PP681140.1 | 37.694 | 45.986 | 47.281 | 47.402 | 49.199 | 49.199 | 51.047 | 49.683 | 82.662 | 91.48 | 100 | 65.496 | 68.008 | 67.092 | 67.726 | 67.89 | 67.83 | 59.903 | 62.512 | 64.993 | 63.79 |
| MW006477.1 | 38.073 | 44.158 | 45.379 | 47.594 | 46.024 | 46.024 | 47.331 | 46.396 | 64.177 | 64.227 | 65.496 | 100 | 83.33 | 83.194 | 83.083 | 83.565 | 83.317 | 67.397 | 70.329 | 84.979 | 81.939 |
| OR413583.1 | 37.33 | 44.109 | 45.777 | 47.577 | 46.633 | 46.633 | 48.782 | 47.494 | 67.26 | 66.832 | 68.008 | 83.33 | 100 | 88.578 | 87.679 | 87.756 | 87.993 | 66.858 | 68.171 | 77.55 | 74.727 |
| MW882934.1 | 37.841 | 44.594 | 45.479 | 47.481 | 46.949 | 46.949 | 48.683 | 47.612 | 66.78 | 66.554 | 67.092 | 83.194 | 88.578 | 100 | 95.555 | 96.09 | 96.385 | 67.311 | 69.036 | 77.905 | 75.341 |
| OP850600.1 | 38.067 | 44.801 | 46.072 | 48.17 | 47.096 | 47.096 | 48.993 | 48.136 | 66.665 | 66.81 | 67.726 | 83.083 | 87.679 | 95.555 | 100 | 99.375 | 98.953 | 67.41 | 69.009 | 78.058 | 75.516 |
| OP819284.1 | 38.003 | 44.937 | 45.837 | 47.912 | 47.314 | 47.314 | 49.213 | 48.273 | 66.938 | 67.015 | 67.89 | 83.565 | 87.756 | 96.09 | 99.375 | 100 | 99.546 | 67.696 | 69.235 | 78.315 | 75.779 |
| OP866730.1 | 38.083 | 45.062 | 45.783 | 47.984 | 47.428 | 47.428 | 49.142 | 48.182 | 66.948 | 67.202 | 67.83 | 83.317 | 87.993 | 96.385 | 98.953 | 99.546 | 100 | 67.765 | 69.338 | 78.326 | 75.782 |
| MZ326168.1 | 38.453 | 41.34 | 42.597 | 45.267 | 43.758 | 43.758 | 44.545 | 43.759 | 59.209 | 59.744 | 59.903 | 67.397 | 66.858 | 67.311 | 67.41 | 67.696 | 67.765 | 100 | 93.698 | 76.485 | 79.769 |
| phiSalP219 | 41.163 | 43.436 | 45.122 | 47.123 | 45.85 | 45.85 | 46.649 | 46.179 | 62.25 | 61.737 | 62.512 | 70.329 | 68.171 | 69.036 | 69.009 | 69.235 | 69.338 | 93.698 | 100 | 79.991 | 82.411 |
| NC_016071.1 | 40.112 | 43.756 | 45.98 | 47.824 | 45.703 | 45.703 | 46.857 | 46.424 | 64.176 | 64.093 | 64.993 | 84.979 | 77.55 | 77.905 | 78.058 | 78.315 | 78.326 | 76.485 | 79.991 | 100 | 93.676 |
| NC_027351.1 | 40.204 | 43.045 | 45.449 | 48.032 | 45.459 | 45.459 | 46.6 | 46.133 | 63.137 | 62.822 | 63.79 | 81.939 | 74.727 | 75.341 | 75.516 | 75.779 | 75.782 | 79.769 | 82.411 | 93.676 | 100 |
|  |  |  |  |  |  |  |  |  |  |  |  |  |  |  |  |  |  |  |  |  |  |
